# Non-Decarboxylative Ruthenium-Catalyzed Rearrangement of 4-Alkylidene-isoxazol-5-ones to Pyrazole- and Isoxazole-4-carboxylic Acids

**DOI:** 10.1021/acs.orglett.2c01135

**Published:** 2022-04-19

**Authors:** Camilla Loro, Letizia Molteni, Marta Papis, Leonardo Lo Presti, Francesca Foschi, Egle M. Beccalli, Gianluigi Broggini

**Affiliations:** † Dipartimento di Scienza e Alta Tecnologia, Università degli Studi dell’Insubria, Via Valleggio 9, 22100, Como, Italy; ‡ DISFARM, Sezione di Chimica Generale e Organica “A. Marchesini”, Università degli Studi di Milano, Via Venezian 21, 20133, Milano, Italy; § Dipartimento di Chimica, Università degli Studi di Milano, via Golgi 19, 20133 Milano, Italy

## Abstract

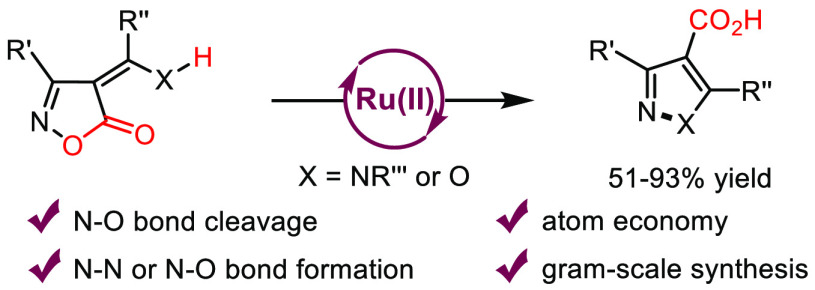

Treatment of 4-(2-hydroaminoalkylidenyl)- and 4-(2-hydroxyalkylidenyl)-substituted isoxazol-5(4*H*)-ones with catalytic amounts of [RuCl_2_(*p*-cymene)]_2_, without any additive, afforded pyrazole- and isoxazole-4-carboxylic acids, respectively. The presence of an intramolecular H-bond in these substrates was the key to divert the classical mechanism toward a ring-opening non-decarboxylative path that is expected to generate a vinyl Ru-nitrenoid intermediate, the cyclization of which affords the rearranged products. A gram scale protocol demonstrated the synthetic applicability of this transformation.

Isoxazol-5-ones have found considerable interest in organic synthesis as building blocks to access acyclic and heterocyclic compounds due to their stability and easy ring opening at the N–O bond.^1^ The different type of the possible transformations mainly depends on the reaction conditions as well as on the structural and electronic properties of the substituent C4 of the ring. Although the manipulation of isoxazol-5-ones has long been used for the preparation of nitrogen-containing five- and six-membered heterocyclic rings,^2^ the utility of transition metal complexes in promoting ring-opening/decarboxylation/cyclization processes has recently emerged. The treatment of these substrates with catalytic amounts of palladium, iridium, iron, rhodium, cobalt, and ruthenium complexes paved the way to useful alternative procedures for the synthesis of pyridine, 2*H*-pyrrole, 2*H*-azirine, and piperidine derivatives.^3^

As a part of our ongoing interest in the synthesis of nitrogen-containing heterocycles,^4^ we have recently focused our attention on ruthenium-catalyzed transformation of isoxazol-5-ones.^5^ In this context, Ru-catalysis is known to allow the conversion of properly 4-substituted isoxazol-5-ones into pyridines, azirines, or benzo[*f*]indole-4,9-diones, as summarized in Scheme 1. In 2016, the group of Okamoto and Ohe performed the conversion of 4-allyl-isoxazol-5-ones into pyridines using [RuCl_2_(*p*-cymene)]_2_ as catalyst in the presence of 5,5′-dimethyl-2,2′-bipyridine as ligand in toluene at 100 °C (Scheme 1, eq 1).^3g^ Later, Jurberg’s group proposed an alternative method for accessing 2,3-disubstituted pyridines by treating 4-benzyl- or 4-alkyl-isoxazolones with acrolein and a catalytic system composed of RuCl_3_/PPh_3_ in ethanol at 80 °C (Scheme 1, eq 2).^3a^ In 2017, Peters and co-workers reported the Ru-catalyzed {[RuCl_2_(*p*-cymene)]_2_ or Ru_3_(CO)_12_ in the presence of 2,2′-bipyridine} preparation of 2*H*-azirines starting from 4-(γ-oxoalkyl)-substituted isoxazol-5-ones (Scheme 1, eq 3).^3e^ In 2020, our group developed the Ru-catalyzed {[RuCl_2_(*p*-cymene)]_2_ in DMSO at 100 °C} divergent conversion of 4-substituted isoxazol-5-ones with a 1,4-naphthoquinone moiety into benzo-fused indole derivatives through a C–H functionalization of the naphthoquinone nucleus (Scheme 1, eq 4).^5^

**Scheme 1:**
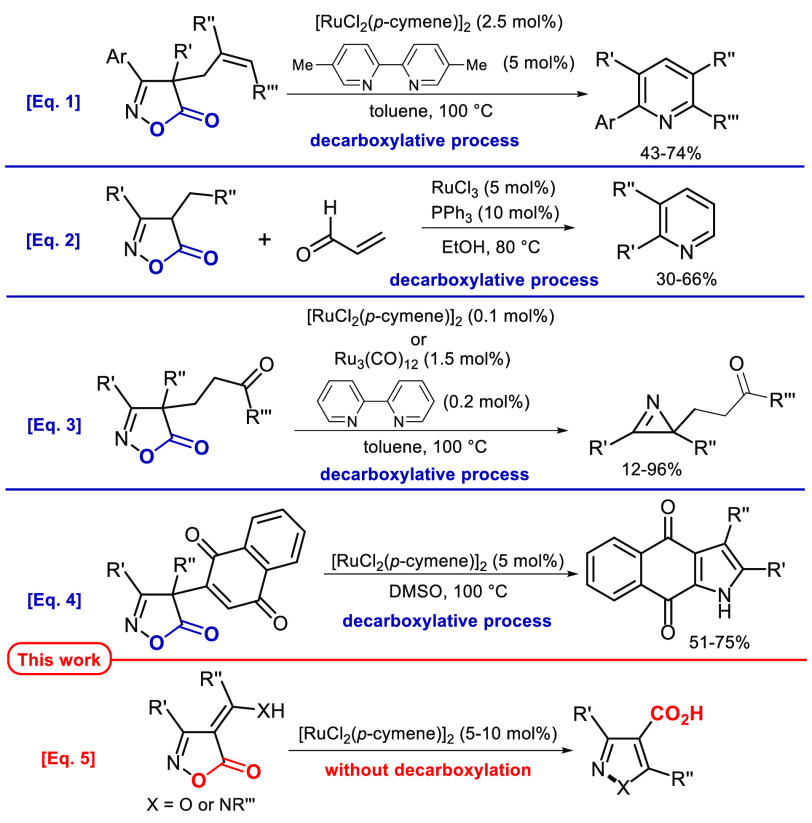
Previous Studies of Ruthenium-Catalyzed Conversion of Isoxazol-5-ones

It is worth noting that all these reactions involve a decarboxylation process that takes place on a ruthenium iminocarboxylic complex, to generate a Ru-vinyl nitrenoid species as a key intermediate. In this context, we decided to investigate the behavior of isoxazol-5-ones bearing 2-hydroaminoalkylidenyl- as well as 2-hydroxyalkylidenyl groups at position 4, as we anticipated that the likely intramolecular H-bond present in such substrates might deflect the classical Ru-catalyzed decarboxylative rearrangement toward a non-decarboxylative concerted path (Figure 1).

**Figure 1:**
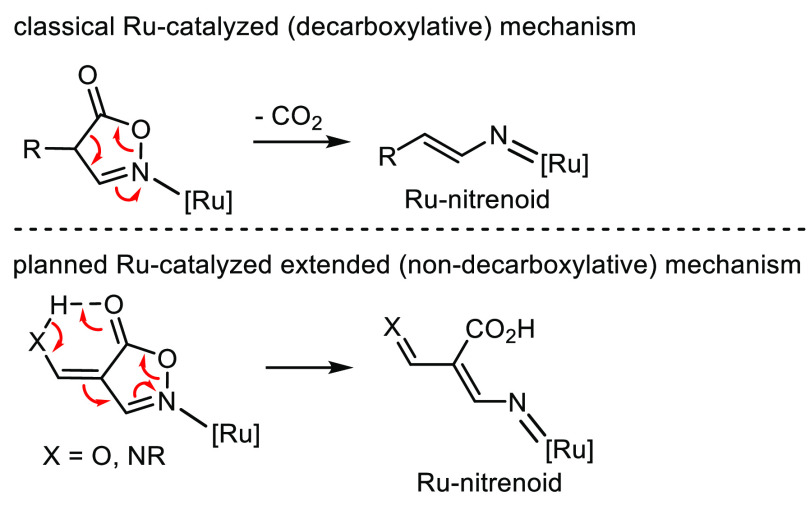
Conception of a Ru(II)-catalyzed deflected mechanism for isoxazol-5-ones.

Herein we report our investigation aimed at the preparation of pyrazole- and isoxazole-4-carboxylic acids (Scheme 1, eq 5).

The pyrazole and isoxazole heterocycles are found incorporated in several molecules of interest in medicinal, crop, and material chemistry.^6^ Consequently, the search for new syntheses of these 1,2-diheteroatom five-membered rings is the object of constant research. In particular, the reported syntheses of pyrazole- and isoxazole-4-carboxylic acids are limited to the oxidation of functional groups already present on the heterocycle, or the hydrolysis of the corresponding esters, in turn not always easily accessible.^7,8^

After preparation of a number of 4-aminoalkylidene-isoxazol-5-ones, following the known procedure,^9^ the isoxazolone **2a** was chosen as our model substrate for the catalytic study, testing first the same reaction conditions used in our previous work {[RuCl_2_(*p*-cymene)]_2_ in DMSO at 100 °C}. Gladly, under these conditions, **2a** afforded the 4-pyrazole carboxylic acid **3a** in 49% isolated yield (Scheme 2). Such a non-decarboxylative process thus validated our initial hypothesis. Changing the solvent to acetonitrile, at 70 °C, raised the yield of **3a** to 68%. Conversely, the use of a Ru(0) species as catalyst, such as Ru_3_(CO)_12_, did not allow the transformation. A higher loading of the catalyst [RuCl_2_(*p*-cymene)]_2_ (i.e., 10 mol %), as well as the presence of a base in the reaction medium (Na_2_CO_3_ or TEA), did not increase the yield of the product.^10^ To substantiate the scalability of this protocol, a gram-scale experiment conducted on a 3.0 mmol scale, performing the reaction in DMSO as solvent and extending the reaction time at 72 h, afforded **3a** in a 71% yield.

**Scheme 2:**
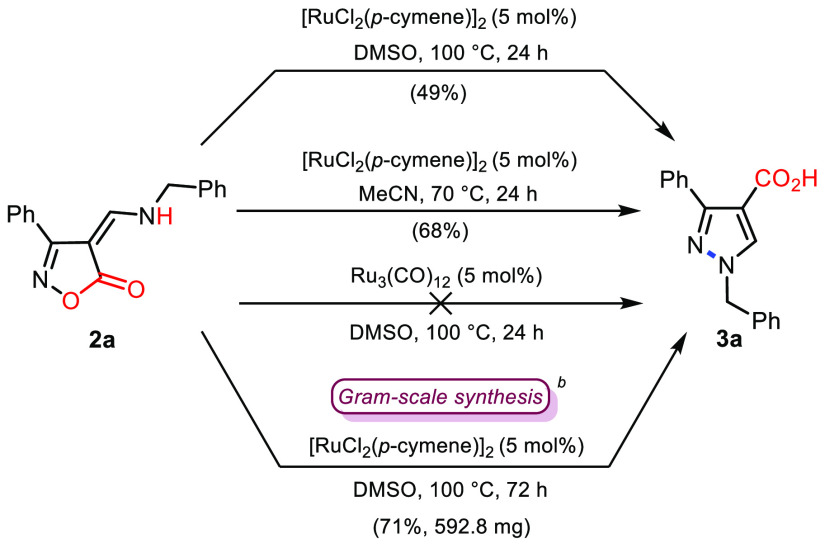
Ru(II)-Catalyzed Conversion of the 4*H*-Isoxazol-5-one **2a** into the Pyrazole-4-carboxylic Acid **3a** Reaction conditions: **2a** (1.0 mmol), catalyst (0.05 mmol), solvent (3 mL), 24 h. Reaction conditions for gram-scale synthesis: **2a** (3.0 mmol), [RuCl_2_(*p*-cymene)]_2_ (0.15 mmol), DMSO (9 mL), 100 °C in oil bath, 72 h.

Although a detailed mechanism for this transformation must await for further studies, a plausible simplified path is proposed in Figure 2. The catalytic cycle is expected to start with the oxidative addition of the metal to the substrate **2a** with generation of the intermediate **A**, the ring opening of which results in the Ru-nitrenoid intermediate **B**.^11^ This latter evolves to **C** or **C′** (directly - *path a* - or by prior addition to the metal - *path b*), affording the final product **3a** by deligandation or reductive elimination of the metal, respectively. The presence of an intramolecular H-bond between the NH group and the oxygen of the carboxylic group was the driving force to hamper the decarboxylative pathway during the ring opening step.^12^

**Figure 2:**
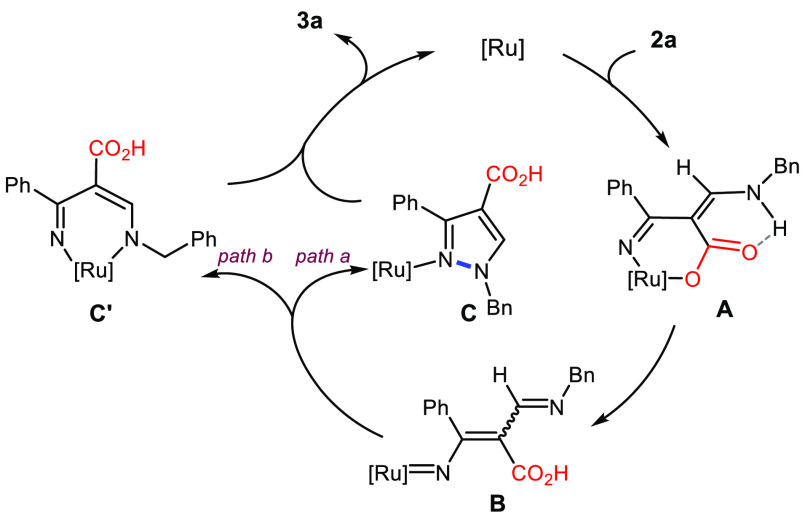
Proposed mechanism for the conversion of the 4*H*-isoxazol-5-one **2a** into **3a**.

We then moved on to test the scope of this rearrangement (Scheme 3). Treatment of isoxazolones incorporating α-unsubstituted secondary enamines with [RuCl_2_(*p*-cymene)]_2_ in MeCN at 70 °C or DMSO 120 °C gave the corresponding 1,3-disubstituted pyrazole-4-carboxylic acids **3b**–**k** in fair to excellent yields. While all these substrates showed full conversion, aryl enamines gave better yields than alkyl enamines. Isoxazolones bearing α-substituted secondary enamines rearranged, too, giving the corresponding 1,3,5-trisubstituted pyrazole-4-carboxylic acids **3l**–**o**. Once again, the rearrangements of *N*-benzyl enamines required longer reaction times and afforded lower yields than those of *N*-aryl enamines. The X-ray crystal structure analysis of compound **3n** provided unambiguous proof for the formation of the pyrazole-4-carboxylic acid.

**Scheme 3:**
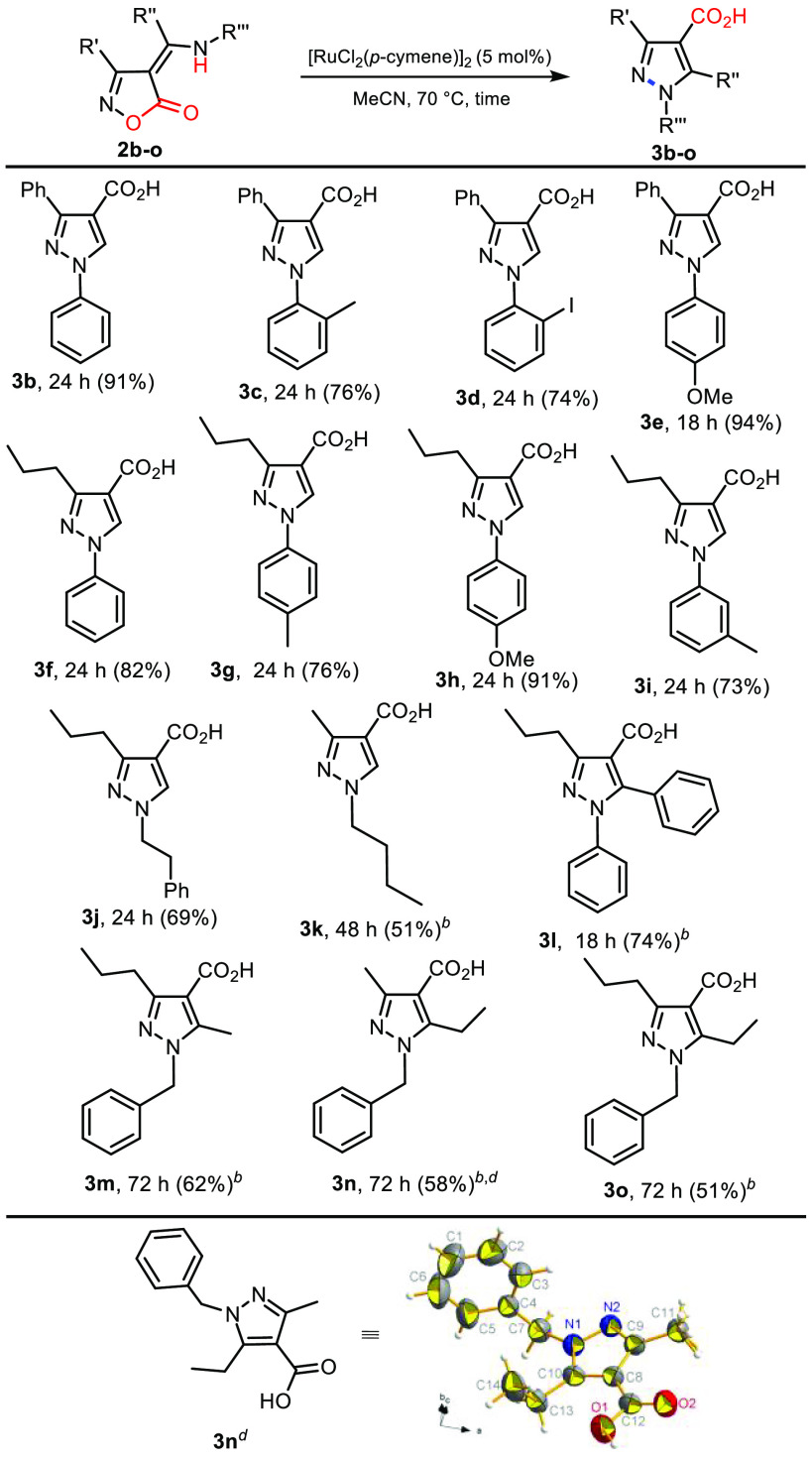
Ru(II)-Catalyzed Conversion of the 4*H*-Isoxazol-5-ones **2a**–**o** into Pyrazole-4-carboxylic Acids^*–*^ Reaction conditions: substrate **2b**–**j** (1.0 mmol), [RuCl_2_(*p*-cymene)]_2_ (0.05 mmol), MeCN (3 mL), 70 °C in oil bath. Reaction conditions: substrate **2k**–**o** (1.0 mmol), [RuCl_2_(*p*-cymene)]_2_ (0.10 mmol), DMSO (3 mL), 120 °C in oil bath. Isolated yields. The molecular structure was determined experimentally through accurate single-crystal X-ray diffraction experiments at room temperature (methyl C11 is rotationally disordered). Thermal ellipsoids are shown at the 50% probability level. Full details in the Supporting Information (SI) (CCDC 2154188).

Worthy of note, isoxazolones bearing primary enamines did not afford the corresponding rearranged product. On one hand, treatment of isoxazolone **2p** with [RuCl_2_(*p*-cymene)]_2_ at 5 mol % in DMSO at 120 °C, or in MeCN at 70 °C, gave a complex mixture of degradation products (Scheme 4). On the other hand, treatment of isoxazolone **2q** under the same reaction conditions led to (*Z*)-1-amino-1-phenyl-1-buten-3-one (**4**) in high yield, thus confirming the inability of the primary aminoalkylidene derivatives to rearrange to pyrazoles.

**Scheme 4:**
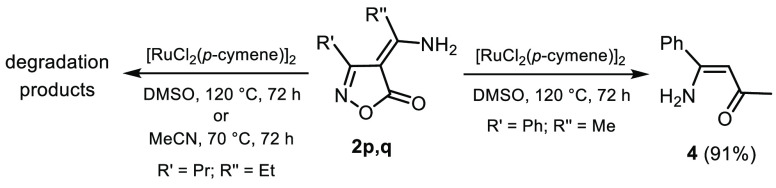
Ru(II)-Catalyzed Reactions of 4*H*-Isoxazol-5-ones Bearing Primary Enamine Groups^,^ Reaction conditions: substrate **2p,q** (1.0 mmol), [RuCl_2_(*p*-cymene)]_2_ (0.05 mmol), solvent (3 mL). Isolated yields.

We subsequently reasoned that 2-hydroxyalkylidenyl moieties linked at position 4 of isoxazol-5-ones—by virtue of the likely intramolecular H-bond between the enol hydrogen atom and the heterocycle carboxyl oxygen—could have analogously deflected the Ru-catalyzed rearrangement, thereby leading to isoxazole-4-carboxylic acids through an intramolecular O–N bond formation. Gratifyingly, treatment of the 2-hydroxyalkylidenyl isoxazole-5-ones **5a**–**f** with catalytic [RuCl_2_(*p*-cymene)]_2_ in MeCN at 70 °C or DMSO at 120 °C generated the expected isoxazole-4-carboxylic acids **6a**–**f** in good to excellent yields (Scheme 5).

**Scheme 5:**
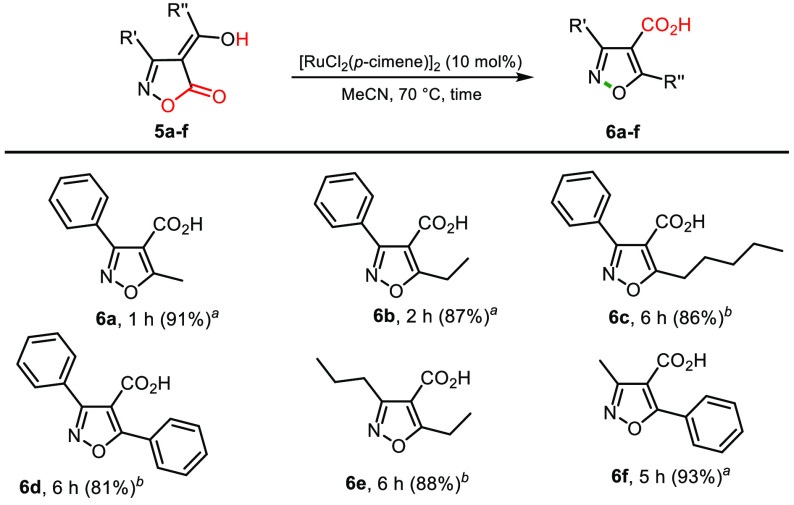
Ru(II)-Catalyzed Conversion of the 4*H*-Isoxazol-5-ones **5a**–**f** into Isoxazole-4-carboxylic Acids^*–*^ Reaction conditions: substrate **5a,b,f** (1.0 mmol), [RuCl_2_(*p*-cymene)]_2_ (0.10 mmol) in MeCN (3 mL), 70 °C in oil bath. Reaction conditions: substrate **5c**–**e** (1.0 mmol), [RuCl_2_(*p*-cymene)]_2_ (0.10 mmol), DMSO (3 mL), 120 °C in oil bath. Isolated yields.

In conclusion, we have successfully developed a ruthenium(II)-catalyzed rearrangement of 4-(2-hydroaminoalkylidenyl)- and 4-(2-hydroxyalkylidenyl)-substituted isoxazol-5(4*H*)-ones, which provided pyrazole and isoxazole-4-carboxylic acids, respectively, in fair to excellent yields. *N*-Aryl and *N*-alkyl secondary enamines were compatible with this reaction, although the reaction on the latter resulted in lower yields. This non-decarboxylative rearrangement corresponds to a detour from the classical reactivity, obtained thanks to the stabilization of the incipient carboxylate anion by the H-bond. The synthetic protocol developed in this work proved to be scalable and contributes to the advancement of the synthesis of pyrazole- and isoxazole-4-carboxylic acids, thus allowing a wider access to these molecules and their application in the research of new material and in medicine.

## Supporting Information Available

Experimental procedures, compound characterization data including copies of ^1^H and ^13^C NMR spectra, and crystallographic data for compound **3n** (PDF)

## Accession Codes

CCDC 2154188 contains the supplementary crystallographic data for this paper. These data can be obtained free of charge via www.ccdc.cam.ac.uk/data_request/cif, or by emailing data_request@ccdc.cam.ac.uk, or by contacting The Cambridge Crystallographic Data Centre, 12 Union Road, Cambridge CB2 1EZ, UK; fax: +44 1223 336033.
