# Supplementary material for: Non-Decarboxylative Ruthenium-Catalyzed Rearrangement of 4-Alkylidene-isoxazol-5-ones to Pyrazole- and Isoxazole-4-carboxylic Acids
Source: Org Lett. 2022 Apr 19;24(16):3092–6. doi: 10.1021/acs.orglett.2c01135 (PMC9062882; doi:10.1021/acs.orglett.2c01135)

Non-Decarboxylative Ruthenium-Catalyzed Rearrangement of 4-Alkylidene-isoxazol-5-ones to Pyrazole- and Isoxazole-4-carboxylic Acids

Camilla Loro,[a] Letizia Molteni,[b] Marta Papis,[a] Leonardo Lo Presti,[c] Francesca Foschi,[a] Egle M. Beccalli[b*] and Gianluigi Broggini[a*]

[a] Dipartimento di Scienza e Alta Tecnologia, Università dell'Insubria, via Valleggio 9, 22100, Como, Italy Phone: (+39)-031-238-6443; e-mail: gianluigi.broggini@uninsubria.it

[b] DISFARM, Sezione di Chimica Generale e Organica "A. Marchesini" Università di Milano, via Venezian 21, 20133 Milano, Italy; e-mail: egle.beccalli@unimi.it

[c] Dipartimento di Chimica, Università degli Studi di Milano, via Golgi 19, 20133 Milano, Italy

Table of Contents

General information S-2
Optimization of the reaction conditions S-3
Preparation of 3-substituted isoxazol-5(4*H*)-ones (1a-c) S-4
General procedure for the preparation of 4-aminoalkylidene-isoxazol-5(4*H*)-ones (2a-q) S-4
General procedure for the preparation of 4-hydroxyalkylidene-isoxazol-5-ones (5a-f) S-9
Synthesis of pyrazole-4-carboxylic acids (3a-o) and isoxazole-4-carboxylic acids (6a-f) S-11
Synthesis of (*Z*)-1-amino-1-phenyl-1-buten-3-one (4) S-16
Synthesis of ethyl (*E*)-3-(4-methyl-5-oxo-3-phenyl-4,5-dihydroisoxazol-4-yl) acrylate (7) S-17
Synthesis of ethyl 4-methyl-5-phenyl-1*H*-pyrrole-2-carboxylate (8) S-17
Synthesis of 3-phenyl-4-(3-phenylallylidene)isoxazol-5(4*H*)-one (9) S-18
Gram-scale synthesis of 1-benzyl-3-phenyl-1*H*-pyrazole-4-carboxylic acid (3a) S-18
Single crystal X-ray diffraction experiment of 3n S-19
^{1}H NMR Spectra of known compounds S-22
^{1}H and ^{13}C NMR Spectra of unknown compounds S-27

General informations: All available chemicals and solvents were purchased from commercial sources and were used without any further purification. Thin layer chromatography (TLC) was performed using 0.25 mm silica gel precoated plates Si 60-F254 (Merck, Darmstadt, Germany) visualized by UV-254 light and CAM staining. Purification by flash column chromatography (FCC) was conducted by using silica gel Si 60, 230-400 mesh, 0.040-0.063 mm (Merck). Melting points were determined on a Stuart Scientific SMP3 and are corrected. ^{1}H and ^{13}C NMR spectra were recorded on a Bruker Avance 400 (400 and 101 MHz, respectively) or Bruker Avance 300 (300 and 75 MHz, respectively); chemical shifts are indicated in parts per million downfield from $SiMe_4$, using the residual proton ($CHCl_3$ = 7.26 ppm) and carbon ($CDCl_3$ = 77.0 ppm) solvent resonances as internal reference. Coupling constants values J are given in Hz. FT-IR spectra were recorded on a Tensor 27 (ATR Diamond) Bruker infrared spectrophotometer and are reported in frequency of absorption (cm^{-1}). Elemental analyses were executed on Perkin-Elmer CHN Analyzer Series II 2400.

Optimization of the reaction conditions:[a]

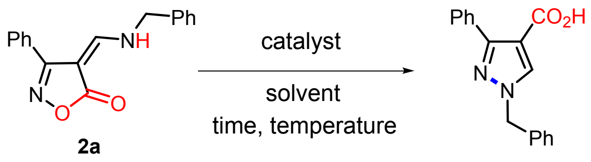

Entry	Catalyst (% mol)	Base (equiv.)	Solvent	Time (h)	Product(s)[b] (% yield)
**1**	$[RuCl_2(p\text{-cymene})]_2$ (5)	-	DMSO	100	**3a** (49)
**2**	$[RuCl_2(p\text{-cymene})]_2$ (5)	-	MeCN	70	**3a** (68)
**3**	$Ru_3(CO)_{12}$ (5)	-	DMSO	100	S.M.
**4**	$[RuCl_2(p\text{-cymene})]_2$ (5)	TEA (1.1)	MeCN	70	S.M.
**5**	$[RuCl_2(p\text{-cymene})]_2$ (5)	Na_2CO_3 (1.1)	MeCN	70	S.M.
**6**	$Pd(OAc)_2$ (10)/ PPh_3 (40)	-	toluene	80	degradation products
**7**	$Pd(PPh_3)_4$ (10)	-	toluene	80	degradation products
**8**	$[Ir(1,5\text{-cod})Cl]_2$ (10)	-	DMSO	80	S.M.
**9**	$[Ir(1,5\text{-cod})Cl]_2$ (10)	-	DMSO	100	S.M.
**10**	$[Ir(1,5\text{-cod})Cl]_2$ (10)	-	DMSO	120	S.M.
**11**[c]	$FeCl_2$ (10)	-	MeCN	70	degradation products

[a] Reaction conditions: (*Z*)-4-((Benzylamino)methylene)-3-phenylisoxazol-5(4*H*)-one **2a** (1.0 mmol), catalyst (5-10 mol%), solvent (3.0 mL), heating in oil bath, 24 hours. [b] Isolated yields. [c] The reaction was carried out at 70 °C in oil bath for 6 hours.

Preparation of 3-substituted isoxazol-5(4*H*)-ones (1a-c):

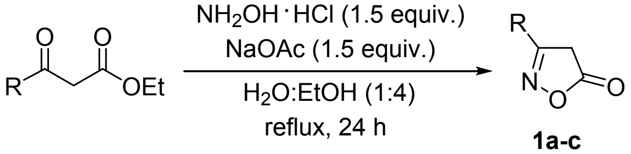

1a-c

A solution of the appropriate β–ketoester (1.0 mmol, 1.0 equiv.) in EtOH (14.6 mL) was added dropwise to a solution of hydroxylamine hydrochloride (104.2 mg, 1.5 mmol, 1.5 equiv.) and sodium acetate (123.0 mg, 1.5 mmol, 1.5 equiv.) in H_2O (3.7 mL). The resulted mixture was stirred at reflux in oil bath for 24 hours. The solvent was evaporated under reduced pressure and the aqueous phase was extracted with AcOEt (15 mL x 3). The organic phase was dried over Na_2SO_4 and the solvent was evaporated under reduced pressure to afford the corresponding 3-substituted isoxazol-5(4*H*)-one **1a-c**. Starting materials, physical data and yields are as follows.

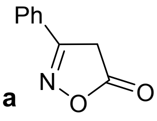

1a

3-Phenylisoxazol-5(4*H*)-one (1a): Starting from ethyl benzoylacetate (192.2 mg) **1a** was obtained as pink solid (157.9 mg, 98%). ^{1}H NMR (400 MHz, $CDCl_3$): δ 7.59 (d, 2H, *J* = 7.4 Hz), 7.48-3.38 (m, 3H), 3.72 (s, 2H). The characterization of product **1a** was consistent with that reported in the literature.[1]

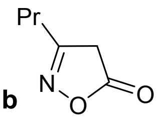

1b

3-Propylisoxazol-5(4*H*)-one (1b): Starting from ethyl 3-oxohexanoate (158.2 mg) **1b** was obtained as red oil (116.9 mg, 92%). ^{1}H NMR (400 MHz, $CDCl_3$): δ 3.39 (s, 2H), 2.42 (t, 2H, *J* = 2.5 Hz), 1.68-1.58 (m, 2H), 0.98 (t, 3H, *J* = 7.4 Hz). The characterization of product **1b** was consistent with that reported in the literature.[1]

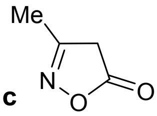

1c

3-Methylisoxazol-5(4*H*)-one (1c): Starting from ethyl acetoacetate (130.1 mg) **1c** was obtained as white oil (94.1 mg, 95%). ^{1}H NMR (400 MHz, $CDCl_3$): δ 3.39 (s, 2H), 2.09 (s, 3H). The characterization of product **1c** was consistent with that reported in the literature.[1]

General procedure for the preparation of 4-aminoalkylidene-isoxazol-5(4*H*)-ones (2a-q):

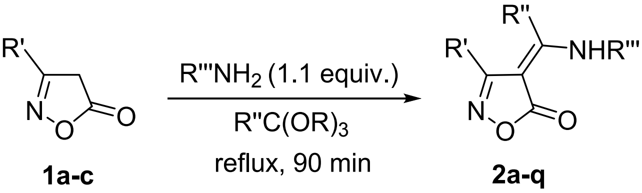

1a-c **2a-q**

---

[1] Torán, R.; Vila, C.; Sanz-Marco, A.; Muñoz, M. C.; Pedro, J. R.; Blay, G. Organocatalytic Enantioselective 1,6-*aza*-Michael Addition of Isoxazolin-5-ones to *p*-Quinone Methides. *Eur. J. Org. Chem.* **2020**, *5*, 627-630.

The appropriate isoxazol-5(4*H*)-one **1a-b** (1.0 mmol, 1.0 equiv.) was dissolved in the suitable orthoester (2 mL) after gentle heating. Then the appropriate primary amine (1.1 mmol, 1.1 equiv.) was added, and the reaction was stirred at reflux in an oil bath.

. After 90 minutes, the solvent was evaporated under reduced pressure. The crude product was purified by silica gel flash column chromatography to afford the corresponding 4-aminoalkylidene-isoxazol-5-one **2a-o**. Starting materials, products, yield, chromatographic eluent and physical data are as follows.

(*Z*)-4-((Benzylamino)methylene)-3-phenylisoxazol-5(4*H*)-one (2a): Starting from **1a** (161.2 mg), benzylamine (120 µL) and trimethyl orthoformate, **2a** was obtained as yellow solid (200.4 mg, 72%), FCC – AcOEt/DCM (1:4). M.p.: 131-133 °C. ^{1}H NMR (400 MHz, $CDCl_3$): δ 9.50 (br s, 1H), 7.67 (s, 1H), 7.57-7.54 (m, 2H), 7.49-7.40 (m, 3H), 7.38-7.35 (m, 3H), 7.31-7.26 (m, 2H), 4.63 (s, 2H). $^{13}C\{^1H\}$ NMR (101 MHz, $CDCl_3$) δ 174.9, 161.4, 153.5, 135.0, 130.4, 129.3, 129.2, 128.7, 128.6, 127.8, 127.6, 90.1, 53.7. IR (neat) *v*: 3091, 1678 cm^{-1}. Anal. Calcd. for $C_{17}H_{14}N_2O_2$: C, 73.37; H, 5.07; N, 10.07. Found: C, 73.21; H, 5.31; N, 9.89.

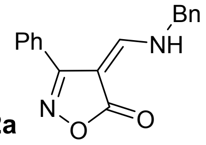

2a

(*Z*)-3-Phenyl-4-((phenylamino)methylene)isoxazol-5(4*H*)-one (2b): Starting from **1a** (161.2 mg), aniline (100 µL) and trimethyl orthoformate, **2b** was obtained as yellow solid (214.0 mg, 81%), FCC – AcOEt/DCM (1:4). ^{1}H NMR (400 MHz, $CDCl_3$): δ 10.85 (d, 1H, *J* = 12.5 Hz), 8.05 (d, 1H, *J* = 13.6 Hz), 7.72 (t, 1H, *J* = 7.6 Hz), 7.66-7.63 (m, 2H), 7.55-7.53 (m, 2H), 7.46-7.42 (m, 2H), 7.29-7.27 (m, 1H), 7.21-7.19 (m, 2H), The characterization of product **2b** was consistent with that reported in the literature.[2]

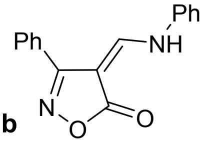

2b

(*Z*)-3-Phenyl-4-((2-tolylamino)methylene)isoxazol-5(4*H*)-one (2c): Starting from **1a** (161.2 mg), *o*-toluidine (117 µL) and trimethyl orthoformate, **2c** was obtained as orange wax (189.2 mg, 68%), FCC – hexane/methanol (9:1). ^{1}H NMR (400 MHz, $CDCl_3$): δ 11.04 (d, 1H, *J* = 12.7 Hz), 8.09 (d, 1H, *J* = 12.7 Hz), 7.66-7.63 (m, 2H), 7.57-7.53 (m, 3H), 7.32-7.26 (m, 2H), 7.22-7.18 (m, 2H), 2.46 (s, 3H). $^{13}C\{^1H\}$ NMR (101 MHz, $CDCl_3$) δ 174.8, 161.1, 146.1, 136.4, 131.6, 130.6, 129.2, 128.4, 128.3, 127.7, 127.6, 126.7, 116.0, 92.9, 17.2. IR (neat) *v*: 3087, 1675 cm^{-1}. Anal. Calcd. for $C_{17}H_{14}N_2O_2$: C, 73.37; H, 5.07; N, 10.07. Found: C, 73.19; H, 4.98; N, 10.21.

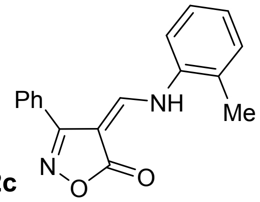

2c

[2] Wentrup, C.; Briehl, H.; Lorenčak, P.; Vogelbacher, U. J.; Winter, H.-W.; Maquestiau, A.; Flammang, R. Primary Ethynamines (HC≡CNH_2, PhC≡ CNH_2), Aminopropadienone (H_2NCH=C=C=O), and Imidoylketene (HN=CHCH=C=O). Preparation and Identification of Molecules of Cosmochemical Interest. *J. Am. Chem. Soc.* **1988**, *110*, 1337-1343.

(*Z*)-4-(((2-Iodophenyl)amino)methylene)-3-phenylisoxazol-5(4*H*)-one (2d): Starting from **1a** (161.2 mg), 2-iodoaniline (240.9 mg) and trimethyl orthoformate, **2d** was obtained as orange wax (226,3 mg, 58%), FCC – hexane/methanol (18:1). ^{1}H NMR (400 MHz, $CDCl_3$): δ 11.00 (d, 1H, *J* = 12.3 Hz), 7.98 (d, 1H, *J* = 12.3 Hz), 7.90 (d, 1H, *J* = 7.9 Hz), 7.67-7.60 (m, 2H), 7.57-7.50 (m, 3H), 7.42 (t, 1H, *J* = 7.6 Hz), 7.22 (d, 1H, *J* = 8.1 Hz), 7.00 (t 1H, *J* = 7.8 Hz). $^{13}C\{^1H\}$ NMR (101 MHz, $CDCl_3$) δ 173.9, 161.2, 145.4, 140.4, 139.2, 130.7, 129.8, 129.3, 128.2, 127.8, 127.7, 116.9, 94.2, 89.7. IR (neat) *v*: 3113, 1684 cm^{-1}. Anal. Calcd. for $C_{16}H_{11}IN_2O_2$: C, 49.25; H, 2.84; N, 7.18. Found: C, 49.38; H, 2.61; N, 7.42.

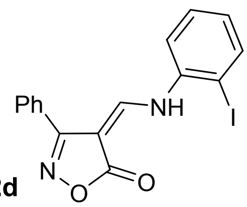

2d

(*Z*)-4-(((4-Methoxyphenyl)amino)methylene)-3-phenylisoxazol-5(4*H*)-one (2e): Starting from **1a** (161.2 mg), *p*-anisidine (128 μL) and trimethyl orthoformate, **2e** was obtained as orange wax (173.6 mg, 59%), FCC – AcOEt/DCM (1:4). ^{1}H NMR (400 MHz, $CDCl_3$): δ 7.84 (br s, 1H), 7.56-7.48 (m, 2H), 7.42-7.38 (m, 3H), 7.28-7.23 (m, 1H), 7.04 (d, 2H, *J* = 8.9 Hz), 6.82 (d, 2H, *J* = 8.9 Hz), 3.69 (s, 3H). $^{13}C\{^1H\}$ NMR (101 MHz, $CDCl_3$) δ 174.7, 161.3, 158.5, 146.2, 131.1, 130.6, 129.4, 129.3, 128.5, 128.4, 127.8, 126.3, 119.6, 115.3, 91.7, 55.6. IR (neat) *v*: 3099, 1680 cm^{-1}. Anal. Calcd. for $C_{17}H_{14}N_2O_3$: C, 69.38; H, 4.79; N, 9.52. Found: C, 69.64; H, 4.60; N, 9.73.

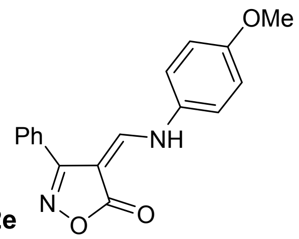

2e

(*Z*)-4-((Phenylamino)methylene)-3-propylisoxazol-5(4*H*)-one (2f): Starting from **1b** (222.1 mg), aniline (100 μL) and triethyl orthoformate, **2f** was obtained as pale brown solid (188.8 mg, 82%), FCC – AcOEt/DCM (1:4). M.p.: 122-123 °C. ^{1}H NMR (400 MHz, $CDCl_3$): δ 7.92 (s, 1H), 7.3 (t, 2H, *J* = 7.8 Hz), 7.19-7.12 (m, 3H), 2.49 (t, 2H, *J* = 7.4 Hz), 1.69-1.59 (m, 2H), 0.91 (t, 3H, *J* = 7.3 Hz). $^{13}C\{^1H\}$ NMR (101 MHz, $CDCl_3$) δ 174.0, 162.1, 144.4, 137.6, 129.8, 126.2, 117.4, 93.0, 27.4, 20.3, 13.6. IR (neat) *v*: 3085, 1679 cm^{-1}. Anal. Calcd. for $C_{13}H_{14}N_2O_2$: C, 67.81; H, 6.13; N, 12.17. Found: C, 68.08; H, 5.93; N, 12.34.

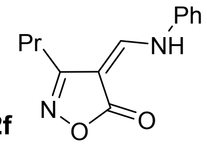

2f

(*Z*)-3-Propyl-4-((4-tolylamino)methylene)isoxazol-5(4*H*)-one (2g): Starting from **1b** (122.1 mg), *p*-toluidine (121 μL) and trimethyl orthoformate, **2g** was obtained as orange solid (188.1 mg, 77%), FCC – AcOEt/hexane (2:3). M.p.: 122-123 °C. ^{1}H NMR (400 MHz, $CDCl_3$): δ 7.88 (s, 1H), 7.23 (d, 2H, *J* = 8.2 Hz), 7.11 (d, 2H, *J* = 8.3 Hz), 2.58 (t, 2H, *J* = 7.4 Hz), 2.37 (s, 3H), 1.80-1.71 (m, 2H), 1.04 (t, 3H, *J* = 7.4 Hz). $^{13}C\{^1H\}$ NMR (101 MHz, $CDCl_3$) δ 174.3, 162.0, 144.1, 136.5, 135.4, 130.5, 117.5, 93.0, 27.7, 20.8, 20.6, 13.8. IR (neat) *v*: 3105, 1687 cm^{-1}. Anal. Calcd. for $C_{14}H_{16}N_2O_2$: C, 68.83; H, 6.60; N, 11.47. Found: C, 69.11; H, 6.38; N, 11.62.

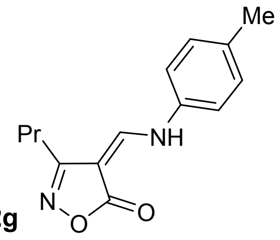

2g

(*Z*)-4-(((4-Methoxyphenyl)amino)methylene)-3-propylisoxazol-5(4*H*)-one (2h): Starting from **1b** (122.1 mg), *p*-anisidine (128 μL) and triethyl orthoformate, **2h** was obtained as red wax (195.2 mg, 75%), FCC – AcOEt/hexane (3:2). ^{1}H NMR (400 MHz, $CDCl_3$): δ 7.75 (s, 1H), 7.08 (d, 2H, *J* = 8.8 Hz), 6.85 (d, 2H, *J* = 8.8 Hz), 3.73 (s, 3H), 2.48 (t, 2H, *J* = 7.4 Hz), 1.70-1.60 (m, 2H), 0.93 (t, 3H, *J* = 7.3 Hz). $^{13}C\{^1H\}$ NMR (101 MHz, $CDCl_3$) δ 174.3, 162.0, 158.2, 144.6, 131.1, 119.3, 115.1, 92.4, 55.5, 27.6, 20.5, 13.8. IR (neat) *v*: 3086, 1673 cm^{-1}. Anal. Calcd. for $C_{14}H_{16}N_2O_3$: C, 64.60; H, 6.20; N, 10.76. Found: C, 64.86; H, 5.94; N, 10.60.

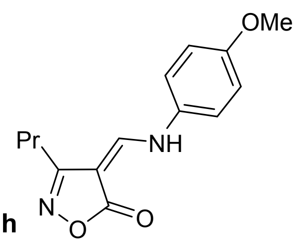

2h

(*Z*)-3-Propyl-4-((3-tolylamino)methylene)isoxazol-5(4*H*)-one (2i): Starting from **1b** (122-1 mg), *m*-toluidine (118 μL) and trimethyl orthoformate, **2i** was obtained as orange wax (195.4 mg, 80%), FCC – AcOEt/hexane (1:1). ^{1}H NMR (400 MHz, $CDCl_3$): δ 10.45 (br s, 1H), 7.85 (s, 1H), 7.24-7.20 (m, 1H), 6.98-6.92 (m, 3H), 2.50 (t, 2H, *J* = 7.5 Hz), 2.30 (s, 3H), 1.71-1.62 (m, 2H), 0.95 (t, 3H, *J* = 7.3 Hz). $^{13}C\{^1H\}$ NMR (101 MHz, $CDCl_3$) δ 174.3, 162.2, 144.2, 140.4, 137.8, 129.9, 127.3, 118.3, 114.7, 93.3, 27.8, 21.4, 20.7, 13.9. IR (neat) *v*: 3097, 1682 cm^{-1}. Anal. Calcd. for $C_{14}H_{16}N_2O_2$: C, 68.83; H, 6.60; N, 11.47. Found: C, 68.57; H, 6.83; N, 11.14.

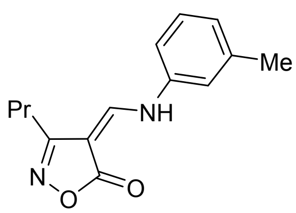

2i

(*Z*)-4-((Phenethylamino)methylene)-3-propylisoxazol-5(4*H*)-one (2j): Starting from **1b** (122.1 mg), phenethylamine (139 μL) and triethyl orthoformate, **2j** was obtained brown wax (162.7 mg, 63%), FCC – AcOEt/hexane (1:1). ^{1}H NMR (400 MHz, $CDCl_3$): δ 8.97 (br s, 1H), 7.24-7.01 (m, 5H), 3.60 (q, 2H, *J* = 6.4 Hz), 2.87 (t, 2H, *J* = 6.7 Hz), 2.26 (t, 2H, *J* = 7.5 Hz), 1.48-1.37 (m, 2H), 0.83 (t, 3H, *J* = 7.4 Hz). $^{13}C\{^1H\}$ NMR (101 MHz, $CDCl_3$) δ 173.7, 161.3, 151.5, 136.0, 128.1, 127.9, 126.0, 88.7, 50.5, 35.9, 26.6, 19.8, 12.8. IR (neat) *v*: 3105, 1686 cm^{-1}. Anal. Calcd. for $C_{15}H_{18}N_2O_2$: C, 69.74; H, 7.02; N, 10.84. Found: C, 69.52; H, 7.23; N, 10.60.

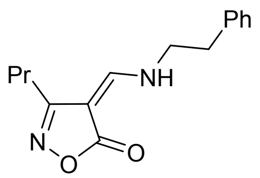

2j

4-((Butylamino)methylene)-3-methylisoxazol-5(4*H*)-one (2k): Starting from **1c** (99.1 mg), butylamine (109 μL) and trimethyl orthoformate, **2k** was obtained as yellow wax (143.9 mg, 79%), FCC – AcOEt/hexane (3:2). ^{1}H NMR (400 MHz, $CDCl_3$): δ 8.86 (br s, 1H), 7.41-7.35 (m, 1H), 3.43 (q, 2H, *J* = 6.7 Hz), 2.13 (s, 3H), 1.69-1.60 (m, 2H), 1.44-1.33 (m, 2H), 0.97-0.89 (m, 3H). $^{13}C\{^1H\}$ NMR (101 MHz, $CDCl_3$) δ 174.7, 158.7, 158.6, 152.2, 49.9, 32.3, 19.6, 13.5, 10.8. IR (neat) *v*: 3270, 1694 cm^{-1}. Anal. Calcd. for $C_9H_{14}N_2O_2$: C, 59.32; H, 7.74; N, 15.37. Found: C, 59.51; H, 7.96; N, 15.59.

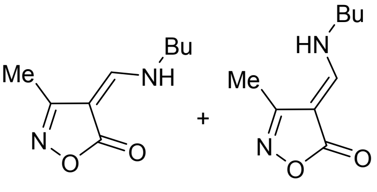

2k

(*Z*)-4-(Phenyl(phenylamino)methylene)-3-propylisoxazol-5(4*H*)-one (2l): Starting from **1b** (122.1 mg), aniline (100 μL) and triethyl orthobenzoate, **2l** was obtained as yellow wax (217.5 mg, 71%), FCC – AcOEt/hexane (2:3). ^{1}H NMR (400 MHz, $CDCl_3$): δ 11.9 (br s, 1H), 7.53-7.48 (m, 1H), 7.44 (t, 2H, *J* = 7.8 Hz), 7.32-7.28 (m, 2H), 7.19-7.09 (m, 3H), 6.81 (d, 2H, *J* = 7.7 Hz), 1.84 (t, 2H, *J* = 7.6 Hz), 1.25-1.15 (m, 2H), 0.60 (t, 3H, *J* = 7.3 Hz). ^{13}C{^{1}H} NMR (101 MHz, $CDCl_3$) δ 163.0, 162.0, 136.8, 131.0, 130.6, 129.1, 128.9, 128.5, 126.7, 124.4, 91.9, 29.8, 20.3, 13.7. IR (neat) *v*: 2994, 1632 cm^{-1}. Anal. Calcd. for $C_{19}H_{18}N_2O_2$: C, 74.49; H, 5.92; N, 9.14. Found: C, 74.67; H, 5.69; N, 9.37.

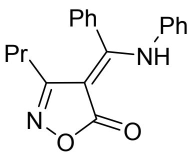

2l

(*Z*)-4-(1-(Benzylamino)ethylidene)-3-propylisoxazol-5(4*H*)-one (2m): Starting from **1b** (122.1 mg), benzylamine (120 μL) and triethyl orthoacetate, **2m** was obtained as yellow wax (191.2 mg, 74%), FCC – AcOEt/hexane (3:2). ^{1}H NMR (400 MHz, $CDCl_3$): δ 10.73 (br s, 1H), 7.33-7.24 (m, 3H), 7.19 (d, 2H, *J* = 8.8 Hz), 4.56 (d, 2H, *J* = 6.2 Hz), 2.53 (t, 2H, *J* = 7.4 Hz), 2.26 (s, 3H), 1.69-1.59 (m, 2H), 0.95 (t, 3H, *J* = 7.4 Hz). ^{13}C{^{1}H} NMR (101 MHz, $CDCl_3$) δ 175.7, 165.3, 161.2, 135.4, 129.3, 128.3, 126.9, 89.6, 47.3, 30.9, 20.5, 15.8, 13.9. IR (neat) *v*: 3090, 1675 cm^{-1}. Anal. Calcd. for $C_{15}H_{18}N_2O_2$: C, 69.74; H, 7.02; N, 10.84. Found: C, 69.90; H, 6.86; N, 10.62.

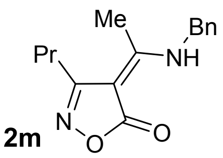

2m

(*Z*)-4-(1-(Benzylamino)propylidene)-3-methylisoxazol-5(4*H*)-one (2n): Starting from **1c** (99.1 mg), benzylamine (120 μL) and triethyl orthopropionate, **2n** was obtained as yellow wax (195.4 mg, 80%), FCC – AcOEt/hexane (3:2). ^{1}H NMR (400 MHz, $CDCl_3$): δ 10.6 (br s, 1H), 7.33-7.25 (m, 3H), 7.21 (d, 2H, *J* = 7.1 Hz), 4.58 (d, 2H), 2.62 (q, 2H, *J* = 7.7 Hz), 2.23 (s, 3H), 1.19 (t, 3H, *J* = 7.7 Hz). ^{13}C{^{1}H} NMR (101 MHz, $CDCl_3$) δ 175.9, 170.5, 157.5, 135.5, 129.2, 128.4, 127.0, 89.1, 46.9, 22.1, 14.6, 12.6. IR (neat) *v*: 3291, 1679 cm^{-1}. Anal. Calcd. for $C_{14}H_{16}N_2O_2$: C, 68.83; H, 6.60; N, 11.47. Found: C, 69.05; H, 6.42; N, 11.19.

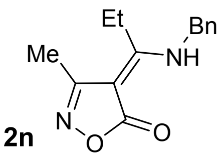

2n

(*Z*)-4-(1-(Benzylamino)propylidene)-3-propylisoxazol-5(4*H*)-one (2o): Starting from **1b** (122.1 mg), benzylamine (120 μL) and triethyl orthopropionate, **2o** was obtained as orange oil (166.1 mg, 61%), FCC – AcOEt/hexane (2:3). ^{1}H NMR (400 MHz, $CDCl_3$): δ 10.7 (br s, 1H), 7.30-7.17 (m, 5H), 4.56 (d, 2H, *J* = 6.1 Hz), 2.59 (q, 2H, *J* = 7.7 Hz), 2.50 (t, 2H, *J* = 7.4 Hz), 1.71-1.61 (m, 2H), 1.17 (t, 3H, *J* = 7.7 Hz), 0.93 (t, 3H, *J* = 7.4 Hz). ^{13}C{^{1}H} NMR (101 MHz, $CDCl_3$) δ 176.1, 170.5, 160.9, 135.6, 129.2, 128.3, 127.0, 88.2, 46.9, 30.6, 22.2, 20.3, 14.0, 12.6. IR (neat) *v*: 3091, 1673 cm^{-1}. Anal. Calcd. for $C_{16}H_{20}N_2O_2$: C, 70.56; H, 7.40; N, 10.29. Found: C, 70.37; H, 7.41; N, 10.58.

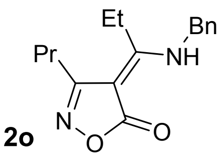

2o

(*Z*)-4-(1-Aminopropylidene)-3-propylisoxazol-5(4*H*)-one (2p): Starting from **1b** (122.1 mg), ammonia (26 µL) and triethyl orthopropionate, **2p** was obtained as orange oil (129.4 mg, 71%), FCC – AcOEt/hexane (1:1). ^{1}H NMR (400 MHz, $CDCl_3$): δ 9.80 (br s, 1H), 6.51 (br s, 1H), 2.70 (q, 2H, J = 7.6 Hz), 2.60 (t, 2H, J = 7.4 Hz), 1.80-1.70 (m, 2H), 1.35 (t, 3H, J = 7.5 Hz), 1.04 (t, 3H, J = 7.4 Hz). ^{13}C{^{1}H} NMR (101 MHz, $CDCl_3$) δ 175.6, 170.4, 161.2, 89.0, 30.8, 26.3, 20.3, 14.0, 11.6. IR (neat) v: 3098, 1664 cm^{-1}. Anal. Calcd. for $C_9H_{14}N_2O_2$: C, 59.32; H, 7.74; N, 15.37. Found: C, 59.55; H, 7.58; N, 15.11.

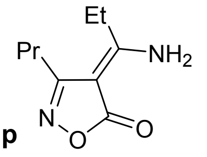

2p

4-(1-Aminoethylidene)-3-phenylisoxazol-5(4*H*)-one (2q): Starting from **1a** (161.2 mg), ammonia (26 µL) and triethyl orthoacetate, **2q** was obtained as orange solid (127.4 mg, 63%), FCC – AcOEt/hexane (1:1). M.p.: 201-203 °C. ^{1}H NMR (400 MHz, DMSO-d$_6$): δ 9.45 (br s, 2H), 7.55-7.46 (m, 7H), 7.34-7.28 (m, 3H), 7.12 (br s, 2H), 2.18 (s, 3H), 1.86 (s, 3H). ^{13}C{^{1}H} NMR (101 MHz, DMSO-d$_6$) δ 188.4, 176.4, 173.8, 167.9, 162.6, 162.4, 133.4, 131.0, 129.6, 128.9, 128.7, 128.5, 127.8, 127.0, 90.2, 87.8, 27.4, 20.0. IR (neat) v: 3102, 1671 cm^{-1}. Anal. Calcd. for $C_{11}H_{10}N_2O_2$: C, 65.34; H, 4.98; N, 13.85. Found: C, 65.52; H, 4.68; N, 13.60.

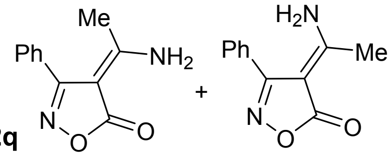

2q

General procedure for the preparation of 4-hydroxyalkylidene-isoxazol-5-ones (5a-f):

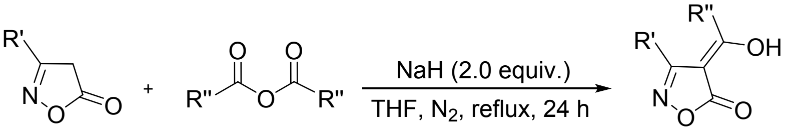

2a-c (2.0 equiv.) 5a-f

The appropriate isoxazol-5(4*H*)-one **1a-c** (1.0 mmol, 1.0 equiv.) was dissolved in dry THF (10 mL) and stirred at 0 °C for 10 minutes. Then NaH (48 mg, 2.0 mmol, 2.0 equiv.) and the appropriate anhydride (2.0 mmol, 2.0 equiv.) were added under N_2 atmosphere.

The resulted solution was stirred for 30 minutes at 0 °C and then allowed to reach room temperature. After, it was stirred at reflux in an oil bath for 24 hours. The solvent was evaporated under reduced pressure, the crude was quenched with an aqueous HCl (2 M, 5 mL) and extracted with DCM (5 mL x 3). The organic phase was dried over Na_2SO_4 and the solvent was evaporated under reduced pressure. The crude product was purified by silica gel column chromatography to afford the corresponding 4-hydroxyalkylidene-isoxazol-5-ones **5a-c**. Starting materials, products, yield, chromatographic eluent and physical data are as follows.

(*Z*)-4-(1-Hydroxyethylidene)-3-phenylisoxazol-5(4*H*)-one (5a): Starting from **1a** (161.2 mg) and acetic anhydride (189 µL), **5a** was obtained as a red solid (158.5 mg, 78%), FCC – AcOEt/hexane (2:3). M.p.: 98-101 °C. ^{1}H NMR (400 MHz, $CDCl_3$): δ 9.38 (br s, 1H), 7.68-7.63 (m, 1H), 7.55-7.48 (m, 4H), 2.11 (s, 3H). ^{13}C{^{1}H} NMR (101 MHz, $CDCl_3$) δ

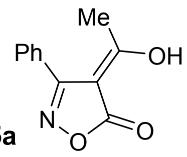

5a

174.8, 163.2, 161.5, 132.2, 130.7, 129.2, 128.9, 128.7, 128.6, 126.6, 98.0, 34.0. IR (neat) *v*: 3198, 1712 cm^{-1}. Anal. Calcd. for $C_{11}H_9NO_3$: C, 65.02; H, 4.46; N, 6.89. Found: C, 65.27; H, 4.29; N, 7.11.

(*Z*)-4-(1-Hydroxypropylidene)-3-phenylisoxazol-5(4*H*)-one (5b): Starting from **1a** (161.2 mg), propionic anhydride (256 μL), **5b** was obtained as a red oil (193.3 mg, 89%). ^{1}H NMR (400 MHz, $CDCl_3$): δ 7.76-7.77 (m, 2H), 7.47-7.37 (m, 3H), 2.59-2.53 (m, 2H), 1.26 (t, 3H, *J* = 7.6 Hz). The characterization of product **5b** was consistent with that reported in the literature.[3]

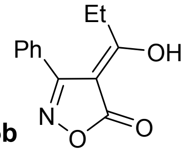

5b

(*Z*)-4-(1-Hydroxyhexylidene)-3-phenylisoxazol-5(4*H*)-one (5c): Starting from **1a** (161.2 mg) and hexanoic anhydride (462 μL), **5c** was obtained as a yellow wax (184.1 mg, 71%), FCC – AcOEt/hexane (2:3). ^{1}H NMR (400 MHz, CD_3OD): δ 7.50-7.38 (m, 2H), 7.33-7.15 (m, 3H), 2.57-2.53 (m, 2H), 1.45-1.31 (m, 2H), 1.25-1.11 (m, 4H), 0.78 (t, 3H, *J* = 6.6 Hz). $^{13}C\{^1H\}$ NMR (101 MHz, CD_3OD) δ 197.5, 179.0, 165.9, 133.9, 130.8, 130.0, 129.8, 128.7, 127.7, 93.2, 40.6, 32.9, 26.4, 23.6, 14.4. IR (neat) *v*: 3214, 1661 cm^{-1}. Anal. Calcd. for $C_{15}H_{17}NO_3$: C, 69.48; H, 6.61; N, 5.40. Found: C, 69.36; H, 6.80; N, 5.57.

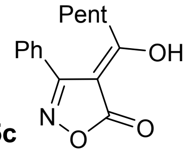

5c

(*Z*)-4-(Hydroxy(phenyl)methylene)-3-phenylisoxazol-5(4*H*)-one (5d): Starting from **1a** (161.2 mg) and benzoic anhydride (377 μL), **5d** was obtained as an orange solid (183.0 mg, 69%), FCC – AcOEt/hexane (2:3). M.p.: 145-147 °C. ^{1}H NMR (400 MHz, $CDCl_3$): δ 10.33 (br s, 1H), 7.48-7.38 (m, 1H), 7.35-7.28 (m, 3H), 7.20-7.11 (m, 6H). $^{13}C\{^1H\}$ NMR (101 MHz, $CDCl_3$) δ 180.3, 177.9, 161.2, 133.1, 130.8, 130.1, 129.6, 128.5, 128.4, 128.3, 128.0, 95.9. IR (neat) *v*: 3062, 1633 cm^{-1}. Anal. Calcd. for $C_{16}H_{11}NO_3$: C, 72.45; H, 4.18; N, 5.28. Found: C, 72.66; H, 3.92; N, 4.96.

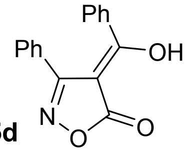

5d

(*Z*)-4-(1-Hydroxypropylidene)-3-propylisoxazol-5(4*H*)-one (5e): Starting from **1b** (122.1 mg) and propionic anhydride (256 μL), **5e** was obtained as a orange solid (168.6 mg, 92%), FCC – AcOEt/hexane (2:3). M.p.: 99-103 °C. ^{1}H NMR (400 MHz, $CDCl_3$): δ 13.3 (br s, 1H), 2.85-2.79 (m, 4H), 1.75-1.65 (m, 2H), 1.15 (t, 3H, *J* = 7.5 Hz), 0.97 (t, 3H, *J* = 7.5 Hz). $^{13}C\{^1H\}$ NMR (101 MHz, $CDCl_3$) δ 173.0, 172.8, 164.1, 95.8, 31.2, 29.3, 19.9, 13.6, 8.8. IR (neat) *v*: 3107, 1666 cm^{-1}. Anal. Calcd. for $C_9H_{13}NO_3$: C, 59.00; H, 7.15; N, 7.65. Found: C, 59.16; H, 7.03; N, 7.92.

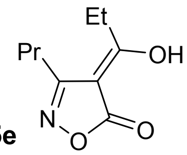

5e

[3] Biju, S.; Reddy, M. L. P.; Cowley, A. H.; Vasudevan, K. V. 3-Phenyl-4-acyl-5-isoxazolonate complex of Tb3+ doped into poly-β-hydroxybutyrate matrix as a promising light-conversion molecular device. *J. Mater. Chem.*, **2009**, *19*, 5179-5187.

(*Z*)-4-(Hydroxy(phenyl)methylene)-3-methylisoxazol-5(4*H*)-one (5f): Starting from **1c** (99.1 mg), and benzoic anhydride (377 μL), **5f** was obtained as an orange oil (164.6 mg, 81%), FCC – AcOEt/hexane (3:7). ^{1}H NMR (400 MHz, $CDCl_3$): δ 11.0 (br s, 1H), 7.69-7.60 (m, 3H), 7.58-7.54 (m, 2H), 2.03 (s, 3H). $^{13}C\{^1H\}$ NMR (101 MHz, $CDCl_3$) δ 179.8, 177.5, 157.7, 133.3, 131.5, 128.8, 128.7, 97.9, 14.4. IR (neat) *v*: 3068, 1654 cm^{-1}. Anal. Calcd. for $C_{11}H_9NO_3$: C, 65.02; H, 4.46; N, 6.89. Found: C, 65.20; H, 4.25; N, 7.12.

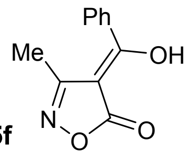

5f

Synthesis of pyrazole-4-carboxylic acids (3a-o) and isoxazole-4-carboxylic acids (6a-f):

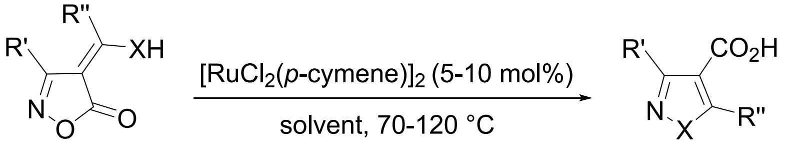

2a-o 5a-f X=NR''', O **3a-o, 6a-f**

In a sealed tube, $[RuCl_2(p\text{-cymene})]_2$ (5 or 10 mol%) was added to a solution of the appropriate 4-alkylidene-isoxazol-5-one **2a-o** or **5a-f** (1.0 mmol, 1.0 equiv.) in the solvent (3 mL). The reaction was stirred at reflux in an oil bath. The mixture was washed with brine (10 mL x 3) and extracted with AcOEt (5 mL x 3). The organic phase was dried over Na_2SO_4 and the solvent was evaporated under reduced pressure. The crude product was purified by silica gel column chromatography to afford the corresponding pyrazole-4-carboxylic acid **3a-o** or isoxazole-4-carboxylic acid **6a-f.** Starting materials, products, yield, chromatographic eluent, reaction time and physical data are as follows.

1-Benzyl-3-phenyl-1*H*-pyrazole-4-carboxylic acid (3a): Starting from **2a** (278.3 mg) and $[RuCl_2(p\text{-cymene})]_2$ (5 mol%, 30.6 mg) in acetonitrile at 70°C for 18 hours, **3a** was obtained as brown solid (189.2 mg, 68%), FCC – AcOEt/hexane (1:1). M.p.: 150-153 °C. ^{1}H NMR (400 MHz, $CDCl_3$): δ 7.97 (s, 1H), 7.79 (d, 2H, *J* = 9.3 Hz), 7.47-7.32 (m, 8H), 5.35 (s, 2H). $^{13}C\{^1H\}$ NMR (101 MHz, $CDCl_3$) δ 167.0, 153.7, 135.9, 134.9, 132.0, 129.3, 129.1, 128.6, 128.5, 128.2, 127.9, 111.0, 56.6. IR (neat) ν 3125, 1724 cm^{-1}. Anal. Calcd. for $C_{17}H_{14}N_2O_2$: C, 73.37; H, 5.07; N, 10.07. Found: C, 73.69; H, 4.86; N, 10.41.

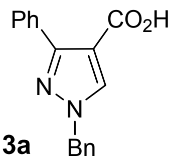

3a

1,3-Diphenyl-1*H*-pyrazole-4-carboxylic acid (3b): Starting from **2b** (264.3 mg) and $[RuCl_2(p\text{-cymene})]_2$ (5 mol%, 30.6 mg) in acetonitrile at 70°C for 24 hours, **3b** was obtained as orange solid (240.5 mg, 91%), FCC – AcOEt/hexane (2:3). M.p.: 204-206 °C. ^{1}H NMR (400 MHz, $CDCl_3$): δ 8.58 (s, 1H), 7.90-7.83 (m, 2H), 7.80-7.74 (m, 2H), 7.54-7.40 (m, 6H). $^{13}C\{^1H\}$ NMR (101 MHz, $CDCl_3$) δ 167.6, 154.5, 139.1, 133.4, 131.8, 129.6, 129.4, 128.8, 128.0, 127.7, 119.7, 119.6, 112.7. IR (neat) *v*: 3156, 1664 cm^{-1}. Anal. Calcd. for $C_{16}H_{12}N_2O_2$: C, 72.72; H, 4.58; N, 10.60. Found: C, 72.66; H, 4.79; N, 10.38.

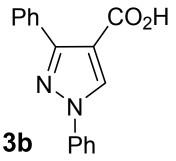

3b

3-Phenyl-1-(2-tolyl)-1*H*-pyrazole-4-carboxylic acid (3c): Starting from **2c** (278.3 mg) and [RuCl$_2$(*p*-cymene)]$_2$ (5 mol%, 30.6 mg) in acetonitrile at 70°C for 24 hours, **3c** was obtained as brown solid (211.4 mg, 76%,), FCC – AcOEt/hexane (2:3). M.p.: 128-132 °C. ^{1}H NMR (400 MHz, CDCl$_3$): δ 8.28 (s, 1H), 7.90 (d, 2H, *J* = 9.3 Hz), 7.48-7.31 (m, 7H), 2.36 (s, 3H). ^{13}C{^{1}H} NMR (101 MHz, CDCl$_3$) δ 168.3, 153.9, 138.8, 137.4, 133.6, 131.8, 131.5, 129.4, 129.2, 128.7, 127.9, 126.8, 126.0, 111.6, 18.1. IR (neat) ν 3181, 1674 cm^{-1}. Anal. Calcd. for $C_{17}H_{14}N_2O_2$: C, 73.37; H, 5.07; N, 10.07. Found: C, 73.52; H, 4.78; N, 10.32.

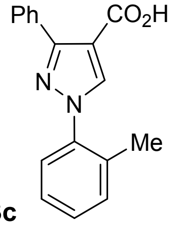

3c

1-(2-Iodophenyl)-3-phenyl-1*H*-pyrazole-4-carboxylic acid (3d): Starting from **2d** (390.2 mg) and [RuCl$_2$(*p*-cymene)]$_2$ (5 mol%, 30.6 mg) in acetonitrile at 70°C for 24 hours, **3d** was obtained as grey solid (288.7 mg, 74%), FCC – AcOEt/hexane (2:3). M.p.: 169-172 °C. ^{1}H NMR (400 MHz, CDCl$_3$): δ 8.39 (s, 1H), 8.00 (d, 1H, *J* = 7.9 Hz), 7.90 (d, 2H, *J* = 7.5 Hz), 7.54-7.43 (m, 5H), 7.23-7.19 (m, 1H). ^{13}C{^{1}H} NMR (101 MHz, CDCl$_3$) δ 167.8, 154.3, 142.3, 140.3, 138.0, 131.6, 130.9, 129.5, 129.2, 128.9, 127.9, 127.8, 111.8, 93.9. IR (neat) ν 3190, 1677 cm^{-1}. Anal. Calcd. for $C_{16}H_{11}IN_2O_2$: C, 49.25; H, 2.84; N, 7.18. Found: C, 49.28; H, 2.58; N, 7.32.

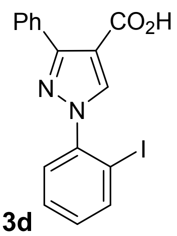

3d

1-(4-Methoxyphenyl)-3-phenyl-1*H*-pyrazole-4-carboxylic acid (3e): Starting from **2e** (294.3 mg) and [RuCl$_2$(*p*-cymene)]$_2$ (5 mol%, 30.6 mg) in acetonitrile at 70°C for 18 hours, **3e** was obtained as yellow solid (276.7 mg, 94%), FCC – AcOEt/hexane (2:3). M.p.: 165-167 °C. ^{1}H NMR (400 MHz, CDCl$_3$): δ 8.48 (s, 1H), 7.87 (d, 2H, *J* = 9.4 Hz), 7.68 (d, 2H, *J* = 9.0 Hz), 7.46-7.40 (m, 3H), 7.01 (d, 2H, *J* = 9.0 Hz), 3.87 (s, 3H). ^{13}C{^{1}H} NMR (101 MHz, CDCl$_3$) δ 167.1, 159.1, 154.2, 133.3, 132.8, 131.9, 129.4, 128.8, 128.0, 121.3, 114.7, 112.1, 55.6. IR (neat) ν 3204, 1700 cm^{-1}. Anal. Calcd. for $C_{17}H_{14}N_2O_3$: C, 69.38; H, 4.79; N, 9.52. Found: C, 69.12; H, 4.91; N, 9.76.

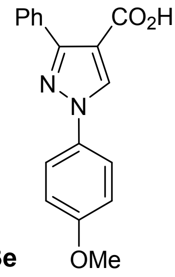

3e

1-Phenyl-3-propyl-1*H*-pyrazole-4-carboxylic acid (3f): Starting from **2f** (230.3 mg) and [RuCl$_2$(*p*-cymene)]$_2$ (5 mol%, 30.6 mg) in acetonitrile at 70°C for 24 hours, **3f** was obtained as brown solid (188.8 mg, 82%), FCC – AcOEt/hexane (1:1). M.p.: 137-138 °C. ^{1}H NMR (400 MHz, CDCl$_3$): δ 8.43 (s, 1H), 7.69 (d, 2H, *J* = 7.9 Hz), 7.47 (t, 2H, *J* = 7.6 Hz), 7.33 (t, 1H, *J* = 7.4 Hz), 2.98 (t, 2H, *J* = 7.5 Hz), 1.86-1.76 (m, 2H), 1.04 (t, 3H, *J* = 7.4 Hz). ^{13}C{^{1}H} NMR (101 MHz, CDCl$_3$) δ 168.9, 157.0, 139.2, 132.3, 129.5, 127.3, 119.5, 112.9, 29.6, 22.3, 14.0. IR (neat) ν 3162, 1682 cm^{-1}. Anal. Calcd. for $C_{13}H_{14}N_2O_2$: C, 67.81; H, 6.13; N, 12.17. Found: C, 67.53; H, 6.25; N, 12.36.

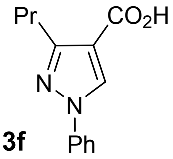

3f

3-Propyl-1-(4-tolyl)-1*H*-pyrazole-4-carboxylic acid (3g): Starting from **2g** (244.1 g) and [$RuCl_2$(*p*-cymene)]$_2$ (5 mol%, 30.6 mg) in acetonitrile at 70°C for 24 hours, **3g** was obtained as grey solid (185.5 mg, 76%), FCC – AcOEt/hexane (2:3). M.p.: 141-143 °C. ^{1}H NMR (400 MHz, $CDCl_3$): δ 8.29 (s, 1H), 7.48 (d, 2H, *J* = 8.4 Hz), 7.17 (d, 2H, *J* = 8.2 Hz), 2.88 (t, 2H, *J* = 7.5 Hz), 2.30 (s, 3H), 1.76-1.67 (m, 2H), 0.95 (t, 3H, *J* = 7.3 Hz). $^{13}C\{^1H\}$ NMR (101 MHz, $CDCl_3$) δ 168.8, 156.8, 137.2, 137.0, 132.1, 130.0, 119.4, 112.8, 29.6, 22.3, 20.9, 14.0. IR (neat) ν 3158, 1634 cm^{-1}. Anal. Calcd. for $C_{14}H_{16}N_2O_2$: C, 68.83; H, 6.60; N, 11.47. Found: C, 68.60; H, 6.81; N, 11.19.

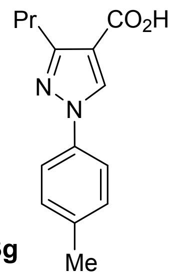

3g

1-(4-Methoxyphenyl)-3-propyl-1*H*-pyrazole-4-carboxylic acid (3h): Starting from **2h** (260.3 mg) and [$RuCl_2$(*p*-cymene)]$_2$ (5 mol%, 30.6 mg) in acetonitrile at 70°C for 24 hours, **3h** was obtained as brown solid (236.9 mg, 91%), FCC – AcOEt/hexane (1:1). M.p.: 126-128 °C. ^{1}H NMR (400 MHz, $CDCl_3$) δ 8.33 (s, 1H), 7.59 (d, 2H, *J* = 8.8 Hz), 6.98 (d, 2H, *J* = 8.8 Hz), 3.85 (s, 3H), 2.96 (t, 2H, *J* = 7.5 Hz), 1.85-1.76 (m, 2H), 1.03 (t, 3H, *J* = 7.3 Hz). $^{13}C\{^1H\}$ NMR (101 MHz, $CDCl_3$) δ 168.7, 158.9, 156.8, 132.9, 132.2, 121.2, 114.6, 112.4, 55.6, 29.6, 22.3, 14.0. IR (neat) ν 3016, 1682 cm^{-1}. Anal. Calcd. for $C_{14}H_{16}N_2O_3$: C, 64.60; H, 6.20; N, 10.76. Found: C, 64.78; H, 5.94; N, 10.51.

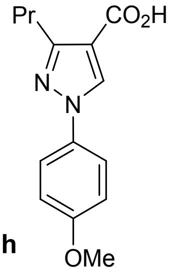

3h

3-Propyl-1-(3-tolyl)-1*H*-pyrazole-4-carboxylic acid (3i): Starting from **2i** (244.3 mg) and [$RuCl_2$(*p*-cymene)]$_2$ (5 mol%, 30.6 mg) in acetonitrile at 70°C for 24 hours, **3i** was obtained as yellow wax (178.3 mg, 73%), FCC – AcOEt/hexane (9:1). ^{1}H NMR (400 MHz, $CDCl_3$): δ 8.42 (s, 1H), 7.55 (s, 1H), 7.47 (d, 2H, *J* = 8.0 Hz), 7.36 (t, 1H, *J* = 7.7 Hz), 7.16 (d, 1H, *J* = 7.7 Hz), 2.98 (t, 2H, *J* = 7.6 Hz), 2.44 (s, 3H), 1.86-1.77 (m, 2H), 1.04 (t, 3H, *J* = 7.3 Hz). $^{13}C\{^1H\}$ NMR (101 MHz, $CDCl_3$) δ 168.7, 157.0, 139.8, 139.2, 132.3, 129.3, 128.2, 120.3, 116.6, 112.7, 29.6, 22.4, 21.5, 14.0. IR (neat) ν 3175, 1682 cm^{-1}. Anal. Calcd. for $C_{14}H_{16}N_2O_2$: C, 68.83; H, 6.60; N, 11.47. Found: C, 68.96; H, 6.48; N, 11.59.

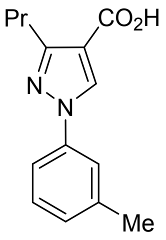

3i

1-Phenethyl-3-propyl-1*H*-pyrazole-4-carboxylic acid (3j): Starting from **2j** (258.3 mg) and [$RuCl_2$(*p*-cymene)]$_2$ (5 mol%, 30.6 mg) in acetonitrile at 70°C for 24 hours, **3j** was obtained as brown solid (178.2 mg, 69%), FCC – AcOEt/hexane (1:1). M.p.: 147-148 °C. ^{1}H NMR (400 MHz, $CDCl_3$): δ 7.66 (s, 1H), 7.33-7.23 (m, 3H), 7.08 (d, 2H, *J* = 8.1 Hz), 4.30 (t, 2H, *J* = 7.0 Hz), 3.18 (t, 2H, *J* = 7.1 Hz), 2.90 (t, 2H, *J* = 6.8 Hz), 1.80-1.69 (m, 2H), 1.00 (t, 3H, *J* = 7.4 Hz). $^{13}C\{^1H\}$ NMR (101 MHz, $CDCl_3$) δ 168.2, 156.2, 137.5, 135.1, 128.7, 128.6, 126.9, 110.4, 53.9, 36.5, 29.5, 22.5, 13.9. IR (neat) ν 3182, 1694 cm^{-1}. Anal. Calcd. for $C_{15}H_{18}N_2O_2$: C, 69.74; H, 7.02; N, 10.84. Found: C, 69.96; H, 6.86; N, 10.51.

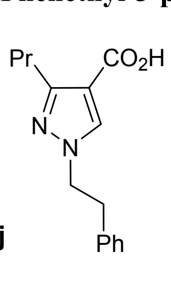

3j

1-Butyl-3-methyl-1*H*-pyrazole-4-carboxylic acid (3k): Starting from **2k** (182.2 mg) and [$RuCl_2$(*p*-cymene)]$_2$ (10 mol%, 61.2 mg) in DMSO at 120°C for 48 hours, **3k** was obtained as yellow oil (92.9 mg, 51%), FCC – AcOEt/hexane (1:1). ^{1}H NMR (400 MHz, $CDCl_3$): δ 7.84 (s, 1H), 4.05 (t, 2H, *J* = 7.6 Hz), 2.30 (s, 3H), 1.90-1.81 (m, 2H), 1.40-1.29 (m, 2H), 0.96 (t, 3H, *J* = 7.4 Hz). ^{13}C{^{1}H} NMR (101 MHz, $CDCl_3$) δ 176.2, 160.6, 133.8, 106.3, 52.2, 32.0, 29.7, 15.5, 14.1. IR (neat) ν 3295, 1717 cm^{-1}. Anal. Calcd. for $C_9H_{14}N_2O_2$: C, 59.32; H, 7.74; N, 15.37. Found: C, 59.47; H, 7.56; N, 15.18.

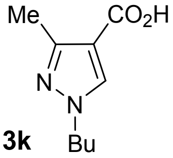

3k

1,5-Diphenyl-3-propyl-1*H*-pyrazole-4-carboxylic acid (3l): Starting from **2l** (306.4 mg) and [$RuCl_2$(*p*-cymene)]$_2$ (10 mol%, 61.2 mg) in DMSO at 120°C for 18 hours, **2l** was obtained as green solid (226.7 mg, 74%), FCC – AcOEt/hexane (2:3). M.p.: 177-180 °C. ^{1}H NMR (400 MHz, $CDCl_3$): δ 7.38-7.29 (m, 3H), 7.27-7.24 (m, 5H), 7.17-7.15 (m, 2H), 2.99 (t, 2H, *J* = 7.6 Hz), 1.87-1.78 (m, 2H), 1.06 (t, 3H, *J* = 7.4 Hz). ^{13}C{^{1}H} NMR (101 MHz, $CDCl_3$) δ 168.0, 156.5, 147.0, 139.2, 130.5, 129.6, 129.0, 128.7, 128.0, 127.7, 125.4, 120.3, 110.2, 30.2, 22.4, 14.2. IR (neat) ν 3167, 1671 cm^{-1}. Anal. Calcd. for $C_{19}H_{18}N_2O_2$: C, 74.49; H, 5.92; N, 9.14. Found: C, 74.71; H, 5.79; N, 9.31.

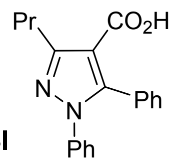

3l

1-Benzyl-5-methyl-3-propyl-1*H*-pyrazole-4-carboxylic acid (3m): Starting from **2m** (258.1 mg) and [$RuCl_2$(*p*-cymene)]$_2$ (10 mol%, 61.2 mg) in DMSO at 120°C for 72 hours, **3m** was obtained as yellow wax (160.0 mg, 62%), FCC – AcOEt/hexane (2:3). ^{1}H NMR (400 MHz, $CDCl_3$): δ 7.27-7.19 (m, 3H), 7.02 (d, 2H, *J* = 8.6 Hz), 5.22 (s, 2H), 2.80 (t, 2H, *J* = 7.5 Hz), 2.39 (s, 3H), 1.70-1.61 (m, 3H), 0.91 (t, 3H, *J* = 7.3 Hz). ^{13}C{^{1}H} NMR (101 MHz, $CDCl_3$) δ 168.5, 154.6, 144.4, 135.0, 127.8, 126.8, 125.6, 107.7, 51.9, 29.0, 21.4, 12.9, 10.5. IR (neat) ν 3186, 1671 cm^{-1}. Anal. Calcd. for $C_{15}H_{18}N_2O_2$: C, 69.74; H, 7.02; N, 10.84. Found: C, 69.96; H, 6.87; N, 10.63.

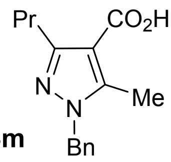

3m

1-Benzyl-5-ethyl-3-methyl-1*H*-pyrazole-4-carboxylic acid (3n): Starting from **2n** (244.3 mg) and [$RuCl_2$(*p*-cymene)]$_2$ (10 mol%, 61.2 mg) in DMSO at 120°C for 72 hours, **3n** was obtained as orange wax (141.7 mg, 58%), FCC – AcOEt/hexane (2:3). ^{1}H NMR (400 MHz, $CDCl_3$): δ 7.27-7.17 (m, 3H), 7.04 (d, 2H, *J* = 7.7 Hz), 5.21 (s, 2H), 2.84 (q, 2H, *J* = 7.5 Hz), 2.41 (s, 3H), 0.99 (t, 3H, *J* = 7.5 Hz). ^{13}C{^{1}H} NMR (101 MHz, $CDCl_3$) δ 169.5, 151.8, 150.9, 136.4, 128.8, 127.9, 126.7, 108.5, 52.7, 18.9, 14.3, 13.2. IR (neat) ν 3063, 1690 cm^{-1}. Anal. Calcd. for $C_{14}H_{16}N_2O_2$: C, 68.83; H, 6.60; N, 11.47. Found: C, 68.95; H, 6.42; N, 11.61.

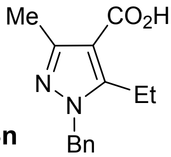

3n

1-Benzyl-5-ethyl-3-propyl-1*H*-pyrazole-4-carboxylic acid (3o): Starting from **2o** (272.4 mg) and [$RuCl_2$(*p*-cymene)]$_2$ (10 mol%, 61.2 mg) in DMSO at 120°C for 72 hours, **2o** was obtained as yellow wax (138.9 mg, 51%), FCC – AcOEt/hexane (2:3). ^{1}H NMR (400 MHz, $CDCl_3$): δ 7.33-7.27 (m, 3H), 7.10 (d, 2H, *J* = 8.6 Hz), 5.29 (s, 2H), 2.93-2.85 (m,

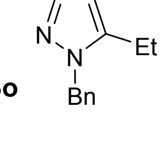

3o

4H), 1.78-1.69 (m, 2H), 1.06 (t, 3H, *J* = 7.4 Hz), 0.98 (t, 3H, *J* = 7.4 Hz). $^{13}C\{^1H\}$ NMR (101 MHz, $CDCl_3$) δ 168.6, 155.7, 150.9, 136.6, 128.8, 127.8, 126.6, 107.7, 52.8, 30.1, 22.4, 18.9, 14.0, 13.2. IR (neat) ν 3173, 1679 cm^{-1}. Anal. Calcd. for $C_{16}H_{20}N_2O_2$: C, 70.56; H, 7.40; N, 10.29. Found: C, 70.82; H, 7.15; N, 10.44.

5-Methyl-3-phenylisoxazole-4-carboxylic acid (6a): Starting from **5a** (203.2 g) and $[RuCl_2(p\text{-cymene})]_2$

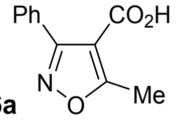

6a

(10 mol%, 61.2 mg) in acetonitrile at 70°C for 1 hour. FCC – AcOEt/hexane (2:3). **6a** White solid (184.9 mg, 91%). ^1H NMR (400 MHz, $CDCl_3$): δ 7.65-7.63 (m, 2H), 7.48-7.43 (m, 3H), 2.77 (s, 3H). The characterization of product **6a** was consistent with that reported in the literature.[4]

5-Ethyl-3-phenylisoxazole-4-carboxylic acid (6b): Starting from **5b** (217.2 mg) and $[RuCl_2(p\text{-cymene})]_2$

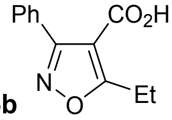

6b

(10 mol%, 61.2 mg) in acetonitrile at 70°C for 2 hours, **6b** was obtained as a grey solid (188.9 mg, 87%), FCC – AcOEt/hexane (2:3). M.p.: 149-151 °C. ^1H NMR (400 MHz, $CDCl_3$): δ 7.67-7.60 (m, 2H), 7.52-7.43 (m, 3H), 3.20 (q, 2H, *J* = 7.6 Hz), 1.40 (t, 3H, *J* = 7.6 Hz). $^{13}C\{^1H\}$ NMR (101 MHz, $CDCl_3$) δ 182.1, 167.4, 162.8, 129.9, 129.4, 128.2, 128.1, 106.6, 21.5, 11.4. IR (neat) ν 2984, 1680 cm^{-1}. Anal. Calcd. for $C_{12}H_{11}NO_3$: C, 66.35; H, 5.10; N, 6.45. Found: C, 66.57; H, 4.97; N, 6.67.

5-Pentyl-3-phenylisoxazole-4-carboxylic acid (6c): Starting from **5c** (259.3 mg) and $[RuCl_2(p\text{-cymene})]_2$

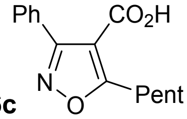

6c

(10 mol%, 61.2 mg) in DMSO at 120°C for 6 hours, **6c** was obtained as yellow wax (222.9 mg, 86%), FCC – AcOEt/hexane (3:7). ^1H NMR (400 MHz, $CDCl_3$): δ 7.65-7.62 (m, 2H), 7.48-7.43 (m, 3H), 3.15 (t, 2H, *J* = 7.6 Hz), 1.85-1.78 (m, 2H), 1.42-1.35 (m, 4H), 0.92 (t, 3H, *J* = 9.9 Hz). $^{13}C\{^1H\}$ NMR (101 MHz, $CDCl_3$) δ 180.2, 165.0, 161.7, 128.9, 128.4, 127.1, 127.0, 105.8, 30.3, 26.6, 25.9, 21.2, 12.8. IR (neat) ν 2960, 1682 cm^{-1}. Anal. Calcd. for $C_{13}H_{17}NO_3$: C, 69.48; H, 6.61; N, 5.40. Found: C, 69.27; H, 6.76; N, 5.65.

3,5-Diphenylisoxazole-4-carboxylic acid (6d): Starting from **5d** (265.3 mg) and $[RuCl_2(p\text{-cymene})]_2$ (10

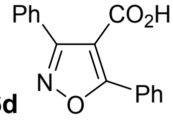

6d

mol%, 61.2 mg) in DMSO at 120°C for 6 hours, **6d** was obtained as a yellow oil (214.9 mg, 81%), FCC – AcOEt/hexane (2:3). ^1H NMR (400 MHz, $CDCl_3$): δ 8.12 (d, 4H, *J* = 7.6 Hz), 7.67-7.58 (m, 2H), 7.53-7.42 (m, 4H). The characterization of product **6d** was consistent with that reported in the literature.[5]

---

[4] Kalin, J. H.; Zhang, H.; Gaudrel-Grosay, S.; Vistoli, G.; Kozikowski, A. P. Chiral Mercaptoacetamides Display Enantioselective Inhibition of Histone Deacetylase 6 and Exhibit Neuroprotection in Cortical Neuron Models of Oxidative Stress. *ChemMedChem* **2012**, *7*, 425-439.

[5] Vitale, P.; Tacconelli, S.; Perrone, M. G.; Malerba, P.; Simone, L.; Scilimati, A.; Lavecchia, A.; Dovizio, M.; Marcantoni, E.; Bruno, A.; Patrignani, P. Synthesis, Pharmacological Characterization, and Docking Analysis of a Novel Family of Diarylisoxazoles as Highly Selective Cyclooxygenase-1 (COX-1) Inhibitors. *J. Med. Chem.* **2013**, *56*, 4277-4299.

5-Ethyl-3-propylisoxazole-4-carboxylic acid (6e): Starting from **5e** (183.2 mg) and [$RuCl_2$(*p*-cymene)]$_2$ (10 mol%, 61.2 mg) in DMSO at 120°C for 6 hours, **6e** was obtained as yellow oil (161.2 mg, 88%), FCC – AcOEt/hexane (3:7). ^{1}H NMR (400 MHz, $CDCl_3$): δ 3.13 (q, 2H, *J* = 7.6, Hz), 2.86 (t, 2H, *J* = 7.5 Hz), 1.83-1.71 (m, 2H), 1.34 (t, 3H, *J* = 7.6 Hz), 1.00 (t, 3H, *J* = 7.5 Hz). $^{13}C\{^1H\}$ NMR (101 MHz, $CDCl_3$) δ 181.3, 166.5, 163.7, 106.4, 27.9, 21.2, 20.9, 13.9, 11.3. IR (neat) ν 2929, 1666 cm^{-1}. Anal. Calcd. for $C_9H_{13}NO_3$: C, 59.00; H, 7.15; N, 7.65. Found: C, 58.73; H, 7.33; N, 7.83.

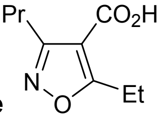

6e

3-Methyl-5-phenylisoxazole-4-carboxylic acid (6f): Starting from **5f** (203.2 mg) and [$RuCl_2$(*p*-cymene)]$_2$ (10 mol%, 61.2 mg) in acetonitrile at 70°C for 5 hours, **6f** was obtained as white solid (188.9 mg, 93%), FCC – AcOEt/hexane (3:7). M.p.: 151-153 °C. ^{1}H NMR (400 MHz, $CDCl_3$): δ 7.93-7.90 (m, 2H), 7.57-7.48 (m, 3H), 2.55 (s, 3H). $^{13}C\{^1H\}$ NMR (101 MHz, $CDCl_3$) δ 174.4, 166.9, 161.3, 131.5, 129.4, 128.4, 126.7, 107.3, 12.3. IR (neat) ν 3136, 1714 cm^{-1}. Anal. Calcd. for $C_{11}H_9NO_3$: C, 65.02; H, 4.46; N, 6.89. Found: C, 65.29; H, 4.28; N, 6.51.

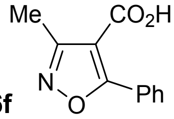

6f

Synthesis of (*Z*)-1-amino-1-phenyl-1-buten-3-one (4):

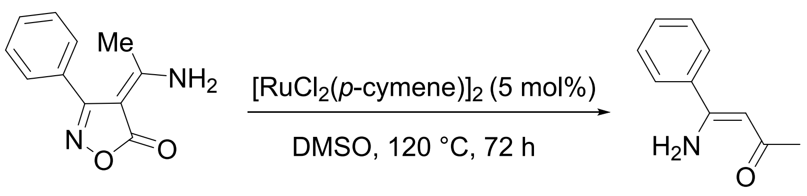

2q **4**

In a sealed tube, [$RuCl_2$(*p*-cymene)]$_2$ (5 mol%, 30.6 mg) was added to a solution of 4-(1-aminoethylidene)-3-phenylisoxazol-5(4*H*)-one **2q** (1.0 mmol, 1.0 equiv., 202.2 mg) in DMSO (3 mL). The reaction was stirred at 120°C for 72 hours in an oil bath. The mixture was washed with brine (10 mL x 3) and extracted with AcOEt (5 mL x 3). The organic phase was dried over Na_2SO_4 and the solvent was evaporated under reduced pressure. Starting materials, products, yield, chromatographic eluent, reaction time and physical data are as follows. Compound **4** was afforded as a yellow oil wax (146.7 mg, 91%) after FCC – AcOEt/hexane (3:2). ^{1}H NMR (400 MHz, $CDCl_3$): δ 9.86 (br s, 1H), 7.51-7.45 (m, 2H), 7.43-7.33 (m, 3H), 5.38 (s, 1H), 5.17 (br s, 1H), 2.08 (s, 3H). The characterization of product **4** was consistent with that reported in the literature.[6]

[6] Di Nunno, L.; Scilimati, A.; Vitale, P. Reaction of 3-phenylisoxazole with alkyllithiums. *Tetrahedron* **2005**, *61*, 2623-2630.

Synthesis of ethyl (*E*)-3-(4-methyl-5-oxo-3-phenyl-4,5-dihydroisoxazol-4-yl) acrylate (7):

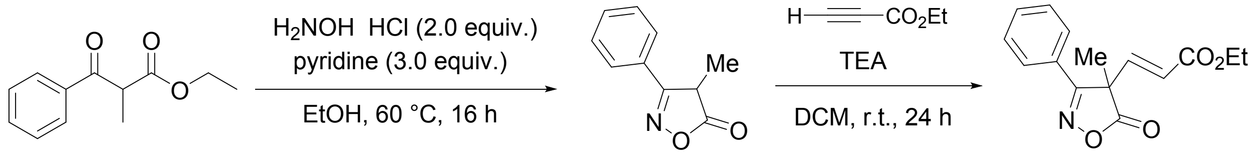

7

To a solution of ethyl 2-methylbenzoylacetate (5.6 mmol, 1.0 equiv.) and pyridine (16.7 mmol, 3.0 equiv., 1.3 mL) in ethanol (1M) was added hydroxykamine hydrochloride (11.1 mmol, 2.0 equiv., 1.30 g) and the reaction mixture was stirred for 16 hours at 60 °C in an oil bath. The mixture was washed with brine (15 mL x 3) and extracted with AcOEt (10 mL x 3). The organic phase was dried over Na_2SO_4 and the solvent was evaporated under reduced pressure. The compound was afforded as a colorless solid (76.5 mg, 41%); m. p. 111- 113 °C and the characterization was consistent with that reported in literature.[7]

In a solution of the previously prepared 4*H*-isoxazol-5-one (1.0 mmol, 1.0 equiv., 175.2 mg), triethylamine (100 µL, 0.72 mmol) in DCM (10 mL), the ethyl propiolate (1.0 mmol, 98.1 mg) was added, and the reaction was stirred at room temperature. The mixture was washed with brine (10 mL x 3) and extracted with AcOEt (5 mL x 3). The organic phase was dried over Na_2SO_4 and the solvent was evaporated under reduced pressure. Compound **7** was afforded as a light-yellow wax (76.5 mg, 28%) after FCC – AcOEt/hexane (3:2). ^{1}H NMR (300 MHz, $CDCl_3$): δ 7.73ì4–7.69 (m, 2H), 7.56–7.43 (m, 3H), 6.97 (d, *J* = 16.0 Hz, 1H), 6.10 (d, *J* = 16.0 Hz, 1H), 4.20 (q, *J* = 7.1 Hz, 2H), 1.77 (s, 3H), 1.28 (t, *J* = 7.1 Hz, 3H). $^{13}C\{^1H\}$ NMR (75 MHz, $CDCl_3$): δ 177.4, 167.1, 164.8, 140.7, 132.1, 129.2, 127.2, 126.6, 125.8, 61.1, 51.1, 20.9, 14.1. Anal. Calcd for $C_{15}H_{15}NO_4$: C, 65.92; H, 5.53; N, 5.13. Found: C, 66.08; H, 5.40; N, 5.22.

Synthesis of ethyl 4-methyl-5-phenyl-1*H*-pyrrole-2-carboxylate (8):

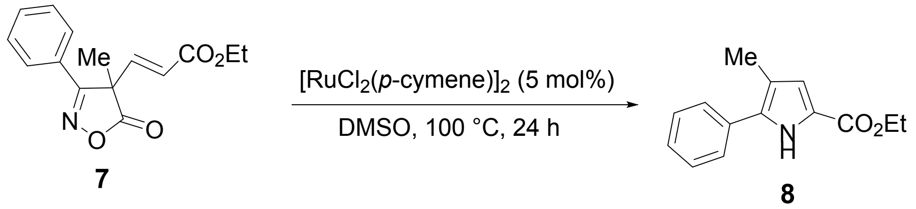

7 **8**

In a sealed tube, $[RuCl_2(p\text{-cymene})]_2$ (0.05 mmol, 30.6 mg) was added to a solution of the ethyl (*E*)-3-(4-methyl-5-oxo-3-phenyk-4,5-dihydroisoxazol-4-yl) acrylate **7** (1.0 mmol, 273.3 mg) in DMSO (9 mL). The reaction was stirred at 100 °C in an oil bath for 24 hours. The mixture was washed with brine (5 mL x 3) and extracted with AcOEt (5 mL x 3). The organic phase was dried over Na_2SO_4 and the solvent was evaporated under reduced pressure. Compound **8** was afforded (183.4 mg, 80%) as a white solid after FCC–

[7] Hellmuth, T. D.-C.; Frey, W.; Peters, R. Regioselective Catalytic Asymmetric C-Alkylation of Isoxazolinones by a Base-Free Palladacycle-Catalyzed Direct 1,4-Addition. *Angew. Chem. Int. Ed.* **2015**, *54*, 2788-2791.

AcOEt/hexane (1:1). ^{1}H NMR (300 MHz, $CDCl_3$): δ 9.62 (br s, 1H), 7.53-7.49 (m, 2H), 7.46-7.39 (m, 2H), 7.33-7.25 (m, 1H), 6.82 (d, J = 2.5 Hz, 1H), 4.27 (q, J = 7.1 Hz, 2H), 2.26 (s, 3H), 1.33 (t, J = 7.1 Hz, 3H). The characterization of product **8** was consistent with that reported in the literature.[8]

Synthesis of 3-phenyl-4-(3-phenylallylidene)isoxazol-5(4*H*)-one (9):

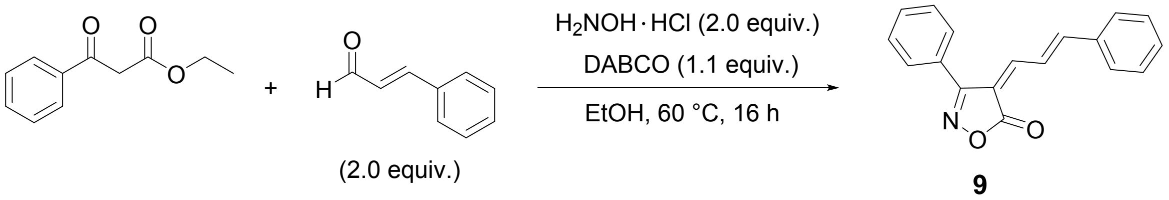

To a solution of ethyl benzoylacetate (2.0 mmol, 2.0 equiv., 384.4 mg), hydroxylamine hydrochloride (2.0 mmol, 2.0 equiv., 138.9 mg) and DABCO (1.1 mmol, 1.1 equiv., 123.4 mg) in ethanol (1M) was added cinnamaldehyde (2.0 mmol, 2.0 equiv., 264.3 m g) and the reaction mixture was stirred for 5 hours at 60 °C in an oil bath. The mixture was washed with brine (15 mL x 3) and extracted with AcOEt (10 mL x 3). The organic phase was dried over Na_2SO_4 and the solvent was evaporated under reduced pressure. The compound **9** was afforded as orange solid (214.7 mg, 39%). ^{1}H NMR (300 MHz, $CDCl_3$): δ 8.45 (dd, 1H, J = 15.5, 11.9 Hz), 7.67-7.55 (m, 7H), 7.49 (d, 1H, J = 11.8 Hz), 7.45-7.43 (m, 3H), 7.30 (d, 1H, J = 15.3 Hz). The characterization was consistent with that reported in literature.[9]

Gram-scale synthesis of 1-benzyl-3-phenyl-1*H*-pyrazole-4-carboxylic acid (3a):

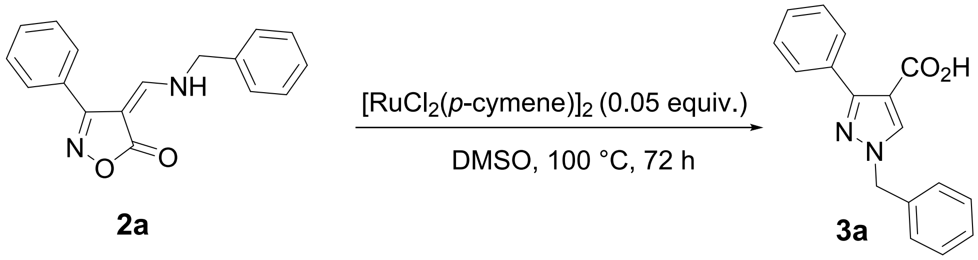

In a sealed tube, $[RuCl_2(p\text{-cymene})]_2$ (0.15 mmol, 91.9 mg) was added to a solution of the (*Z*)-4-((benzylamino)methylene)-3-phenylisoxazol-5(*4H*)-one **2a** (3.0 mmol, 834.9 mg) in DMSO (9 mL). The reaction was stirred at 100 °C in an oil bath. The mixture was washed with brine (15 mL x 3) and extracted with AcOEt (15 mL x 3). The organic phase was dried over Na_2SO_4 and the solvent was evaporated under reduced pressure. Compound **3a** was afforded (592.8 mg, 71%) as a brown solid after FCC– AcOEt/hexane (1:1).

[8] Yang, H.-B.; Selander, N. Divergent Iron-Catalyzed Coupling of O-Acyloximes with Silyl Enol Ethers. *Chem. Eur. J.* **2017**, *23*, 1779-1783.
[9] Mirzazadeh, M.; Mahdavinia, G. H. Fast and Efficient Synthesis of 4-Arylidene-3-phenylisoxazol-5-ones. *E-J. Chem.* **2012**, *9*, 425-429.

Single crystal X-ray diffraction experiment of 3n

CCDC 2154188 contains the supplementary crystallographic data for this paper. These data can be obtained free of charge from The Cambridge Crystallographic Data Centre via www.ccdc.cam.ac.uk/structures.

The material is made up by yellowish brown blades, sticked to the bottom of the crystallization tube. A suitable untwinned specimen was cut from a larger agglomerate with a stainless steel microblade and polished by mechanical ablation in a drop of perfluorinated oil. The habit of the crystal was blade-like, colour yellowish brown, transparent, with dimensions ≈ 0.0.425 x 0.175 x 0.050 mm (Figure 1). The crystal was mounted on the top of a glass capillary fiber with perflorinated oil as a glue.

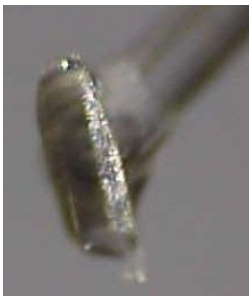

Figure 1. Single crystal sample of **3n**, mounted on the top of a capillary fibre.

X-ray diffraction data were collected on a Bruker AXS smart APEX three-circle diffractometer equipped with a CCD detector, using graphite-monochromatized Mo Kα radiation (λ = 0.71073 Å) at a nominal source power of 50 kV x 30 mA. A high-redundant data collection strategy was employed to record a 100 % complete sphere of data up to a maximum resolution of 0.9 Å. A higher resolution was not achieved due to the poor scattering power of the sample.

The compound crystallizes in the monoclinic centric $P2_1/c$ space group with 4 formulae in cell and 1 molecule per asymmetric unit. Cell parameters are a = 11.7295(6) Å, b = 14.1389(10) Å, c = 8.0853(9) Å, β = 90.607(5) deg, V = 1340.8(2) Å^3, as estimated from the least-squares fitting against 1138 intense reflections among 4.5 and 39.8 deg in 2θ. The structure was solved by direct methods and refined within the independent atom approximation with the shelx suite of programs.[10] The final least-squares model (Figure 2) had agreement factors as low as R1(F) = 0.0392 for 1336 Fo > 4σ(Fo), 0.0665 for all the 19221 independent data, wR(F2) = 0.1171 for all the independent data and goodness-of-fit 0.996. The highest Fourier residuals were $\Delta\rho_{MAX/MIN}$ = +0.092/ –0.166 e/Å^3.

The terminal methyl group at C11 (Figure 2) is rotationally disordered through two non-equivalent orientations, with least-squares estimates of the relative weights as high as 0.6(1) and 0.4(1). More precise determinations require better couting statistics and will be possibly the object of future experiments at lower temperatures.

[10] Sheldrick, G. M. Crystal structure refinement with SHELXL. *Acta Cryst.* **2015**, *C71*, 3-8.

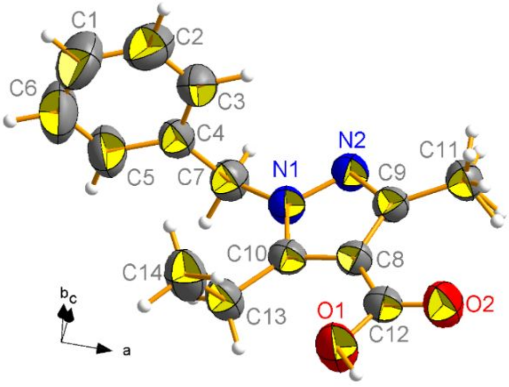

Figure 2. Asymmetric unit of **3n**, with the atom-numbering scheme and the crystallographic reference system. Thermal ellipsoids at RT were drawn at the 50 % probability level. Atoms are represented with the usual colour code (C: gray; N: blue; O: red; H: white).

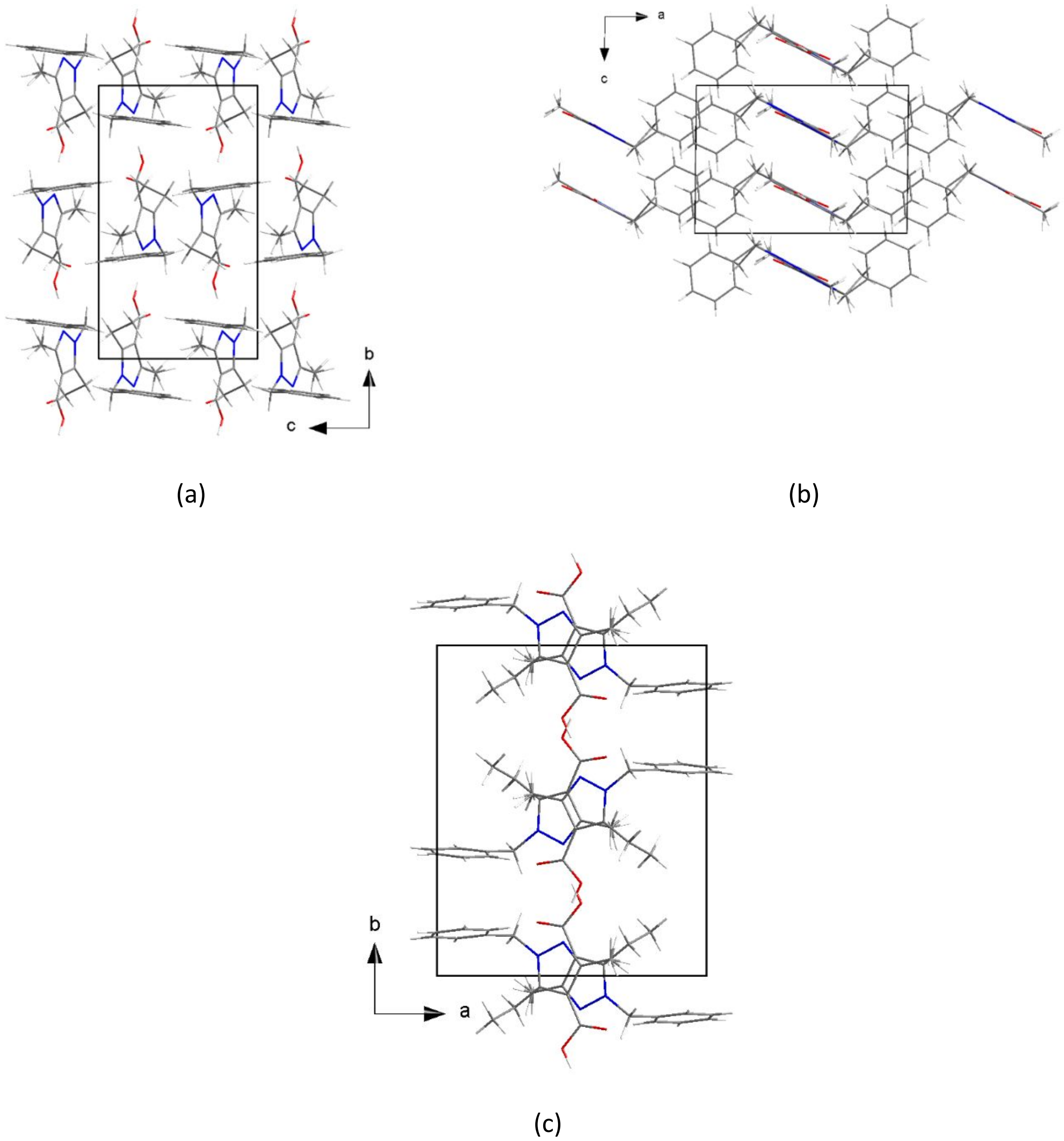

Figure 3. Wires–stick representation of the crystal packing of **3n** at RT, as seen along the *a* (a), *b* (b) and *c* (c) cell axes.

Figure 3 shows the main packing motifs in the (*b,c*), (*a,c*) and (*a,b*) planes. Molecules are organized into antiparallel rows that run along the *b* axis (Figure 2a). These are connected by allegedly strong COOH···N hydrogen bonds that involve the acidic function and the substituent-free N2 nitrogen atom (Table 1). The hydrophobic phenyl substituents are orented orthogonally with respect to the hydrogen-bonded motif (Figure 3b). Stacking interactions, if any, are very weak and likely do not contribute to the overall crystal stability.

Table 1. Geometric parameters for intermolecular hydrogen-bonded contact in **3n** at room temperature. Values in Å and deg, with sensible estimated standard deviations reported in parentheses.

D–H···O	D–H	H···A	D···A	D-H···A	Symmetry operation
O1–H1O···N2	0.91	1.80	2.6840(3)	165	1-x,-1/2+y,1/2-z

^{1}H-NMR spectra of known compounds

3-Phenylisoxazol-5(4*H*)-one (1a)

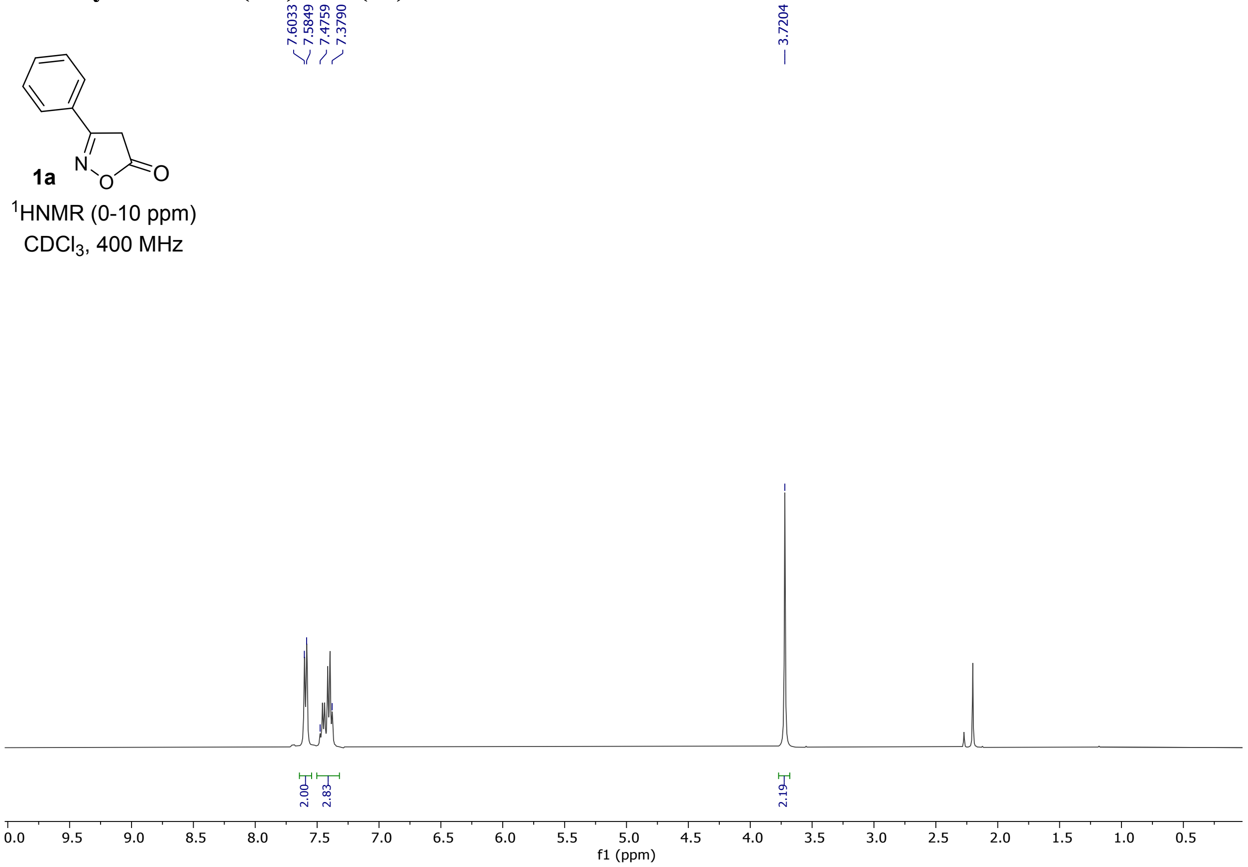

3-Propylisoxazol-5(4*H*)-one (1b)

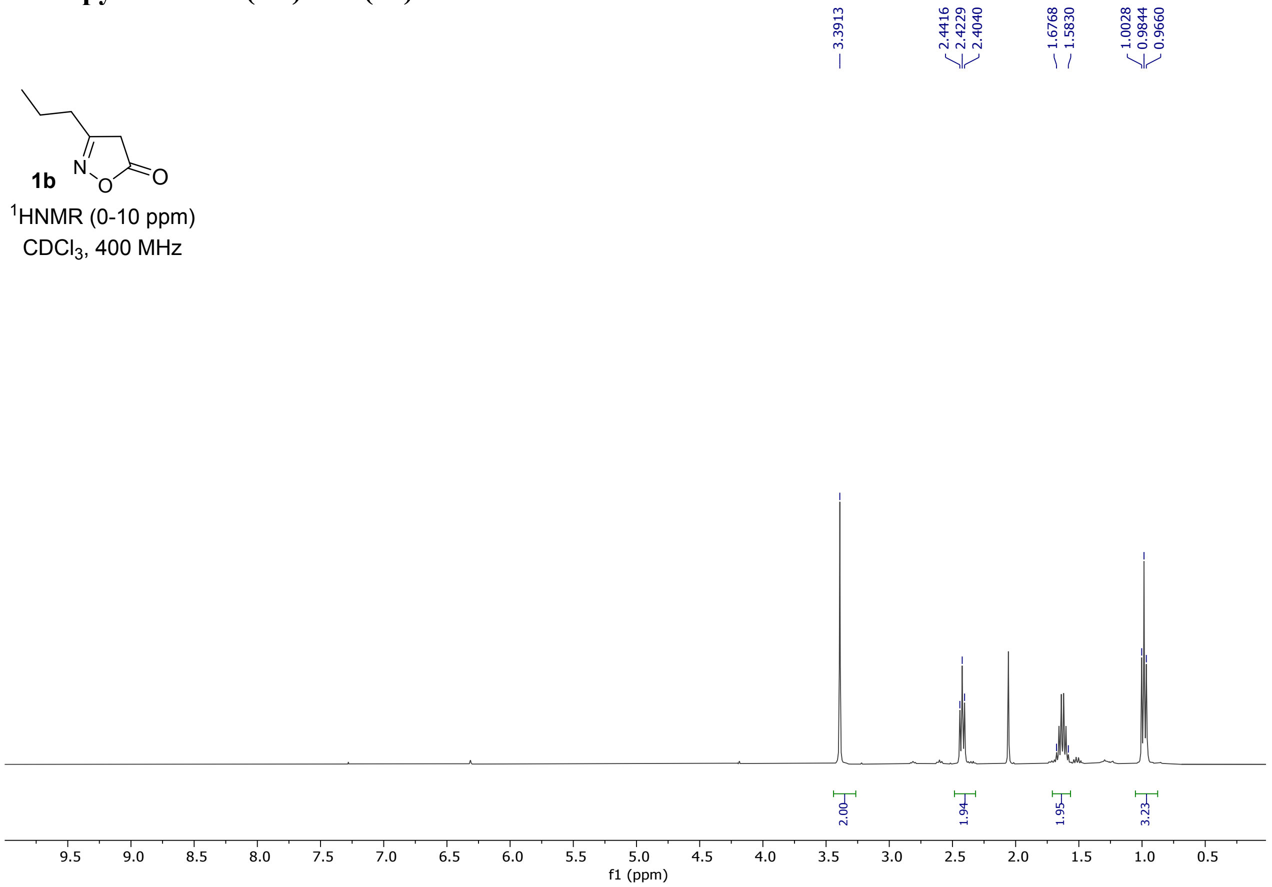

3-Methylisoxazol-5(4*H*)-one (1c)

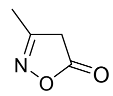

1c

^{1}HNMR (0-10 ppm)
$CDCl_3$, 400 MHz

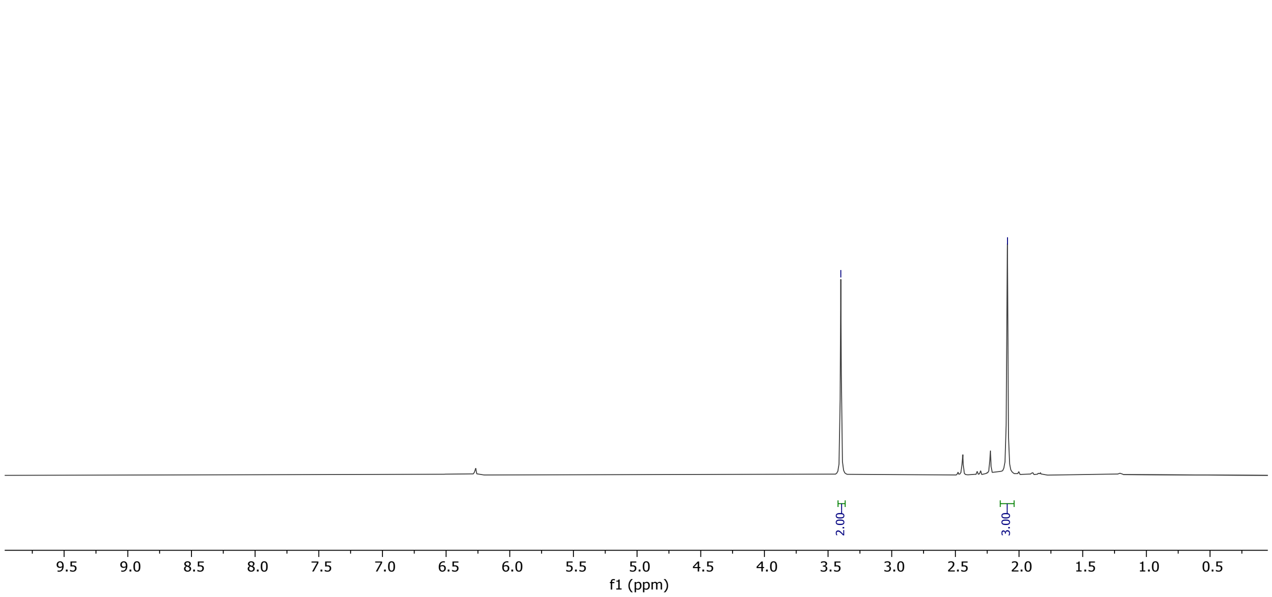

(*Z*)-3-Phenyl-4-((phenylamino)methylene)isoxazol-5(4*H*)-one (2b)

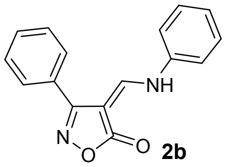

2b

^{1}HNMR (0-12 ppm)
$CDCl_3$, 400 MHz

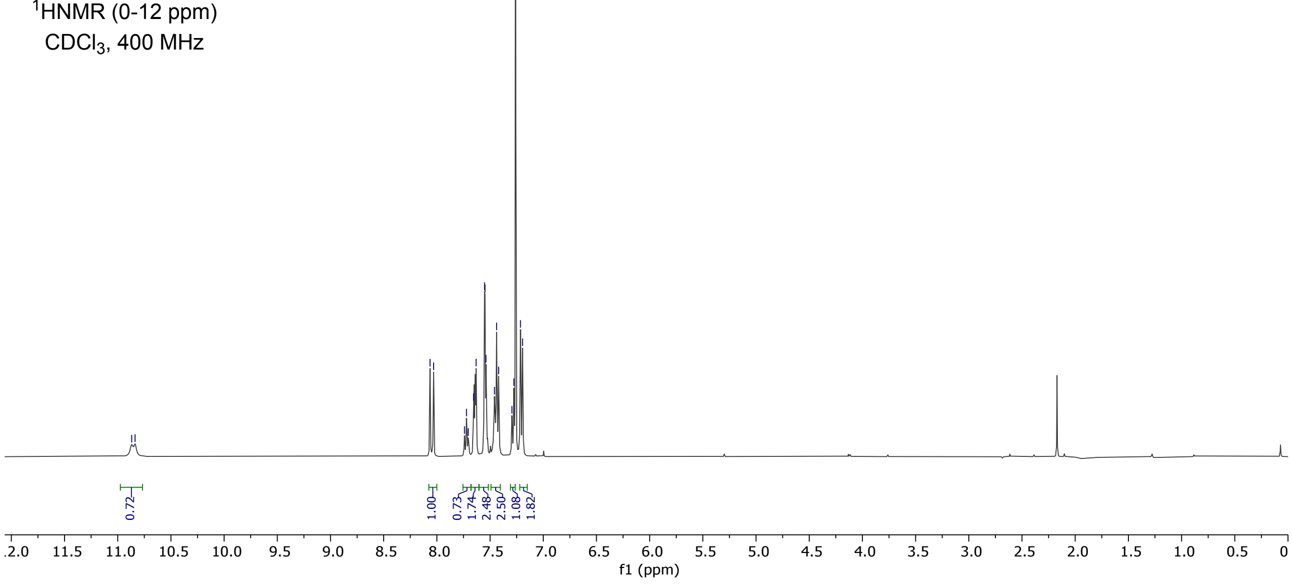

(*Z*)-4-(1-Hydroxypropylidene)-3-phenylisoxazol-5(4*H*)-one (5b)

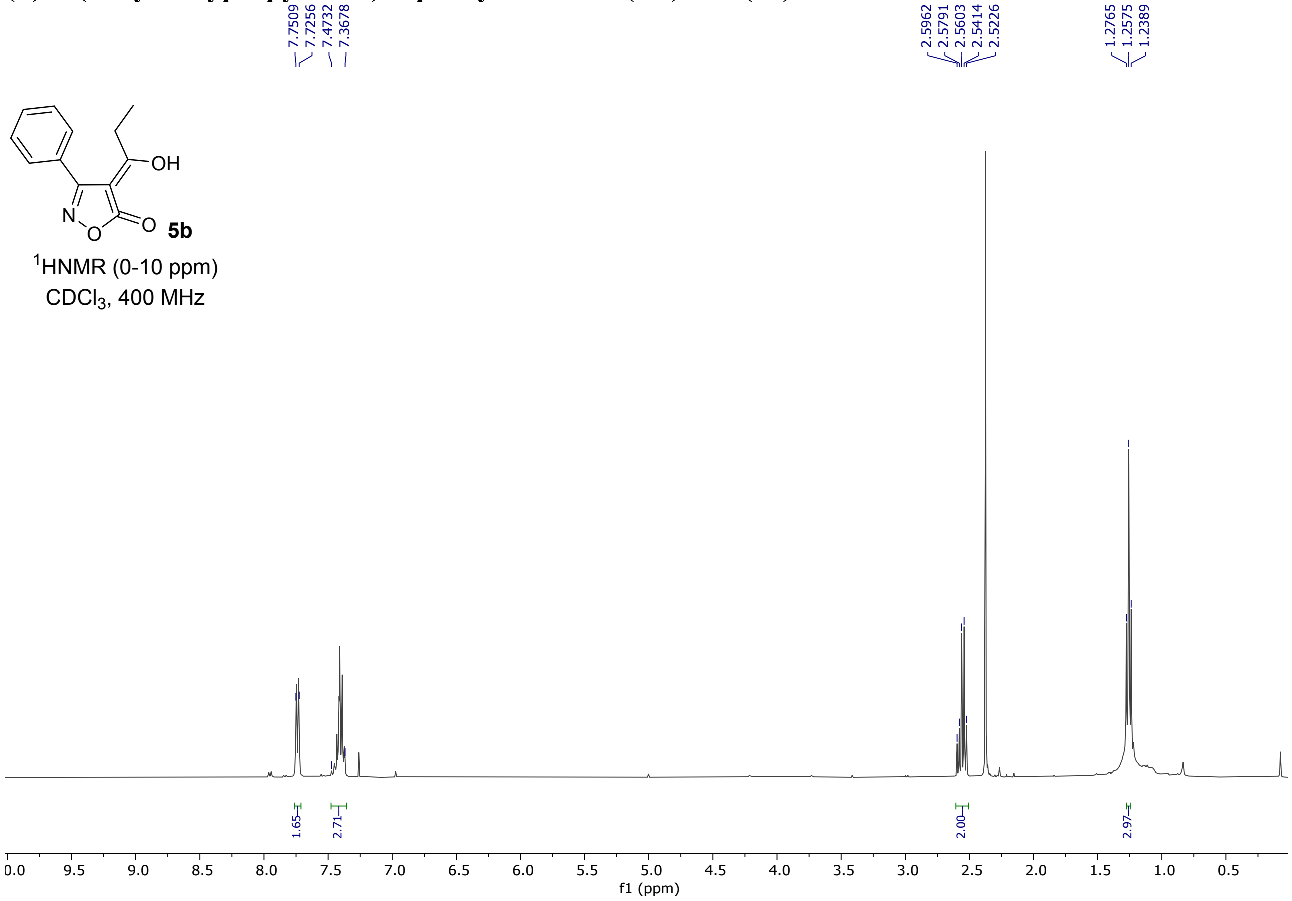

5-Methyl-3-phenylisoxazole-4-carboxylic acid (6a)

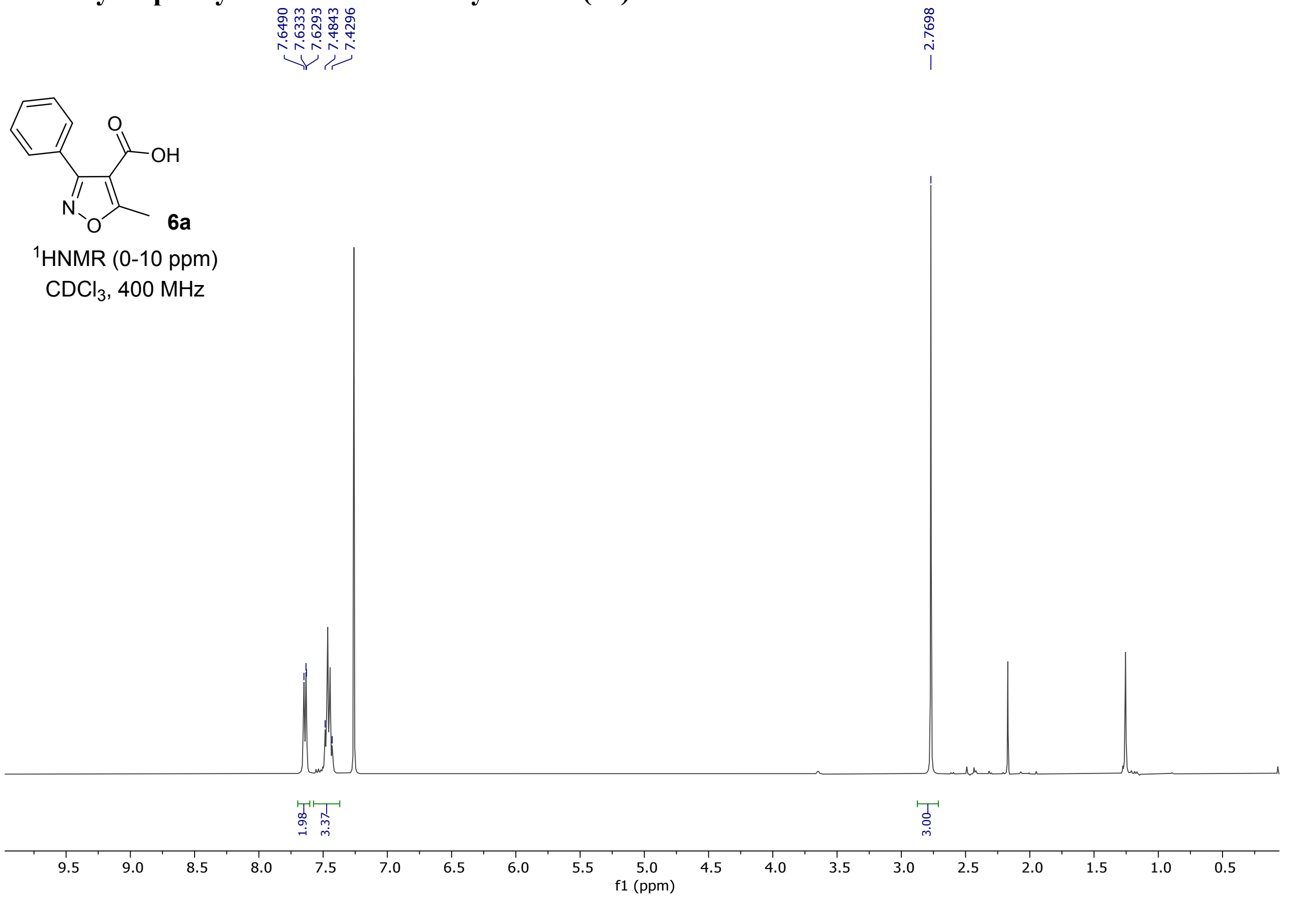

3,5-Diphenylisoxazole-4-carboxylic acid (6d)

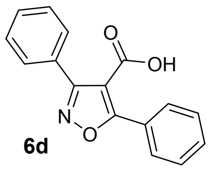

6d

^{1}HNMR (0-10 ppm)
$CDCl_3$, 400 MHz

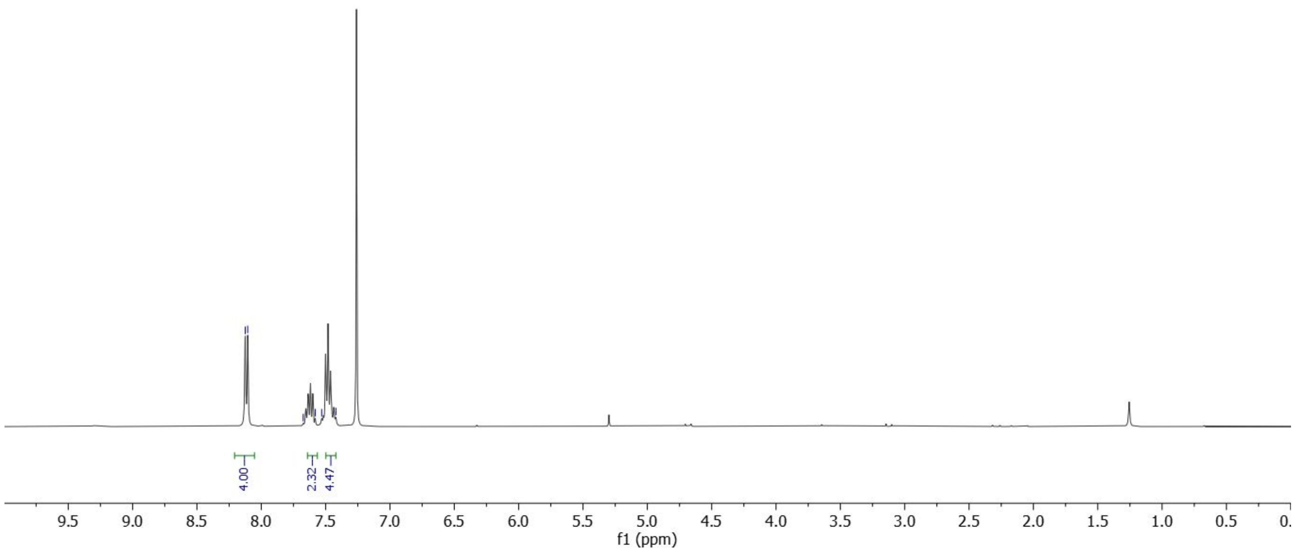

(*Z*)-1-Amino-1-phenyl-1-buten-3-one (4)

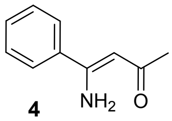

4

^{1}HNMR (0-11 ppm)
$CDCl_3$, 400 MHz

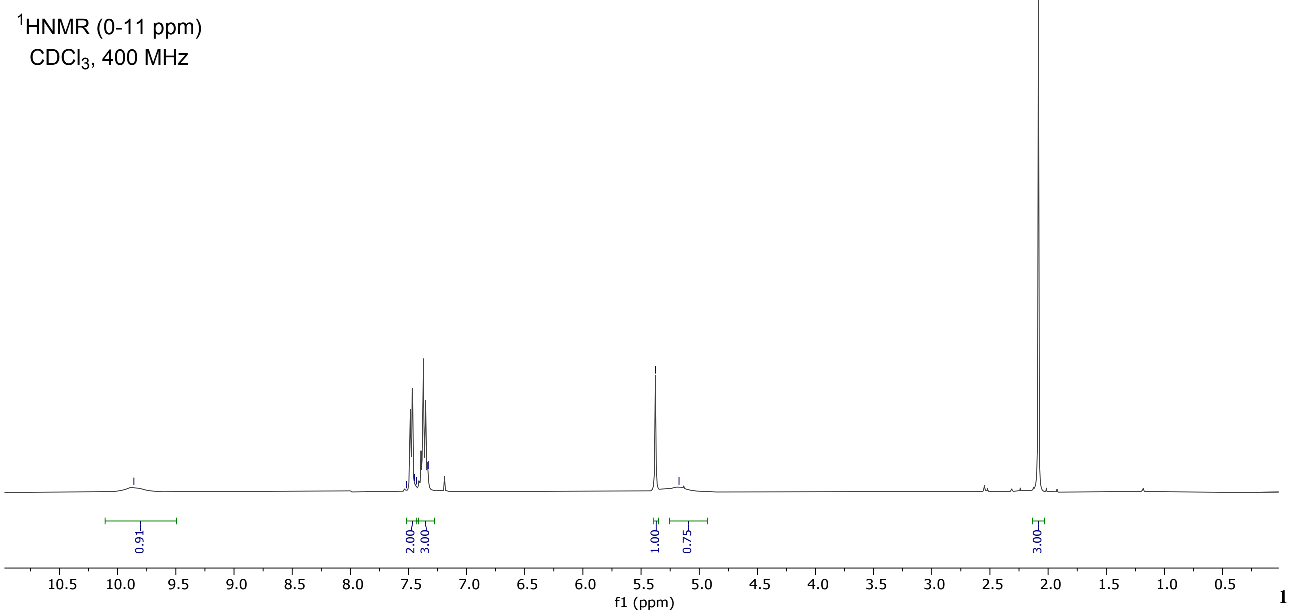

1

Ethyl 4-methyl-5-phenyl-1*H*-pyrrole-2-carboxylate (8)

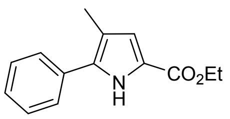

8

^{1}HNMR (0-11 ppm)
$CDCl_3$, 300 MHz

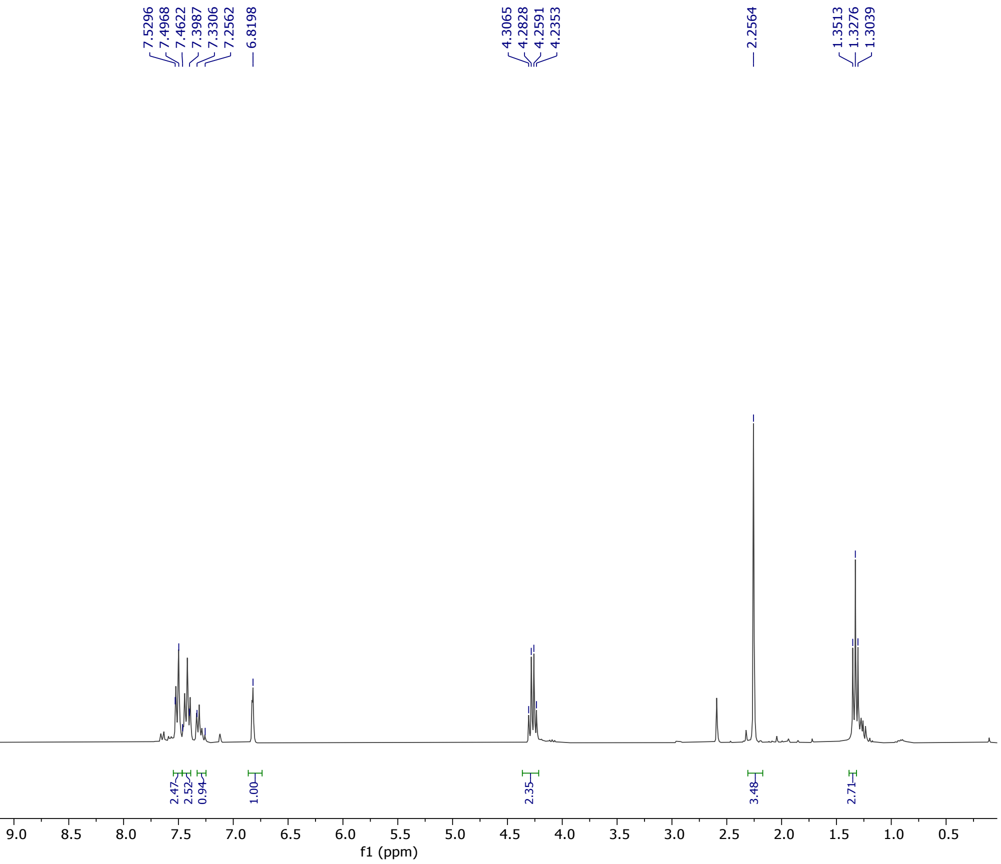

3-Phenyl-4-(3-phenylallylidene)isoxazol-5(4*H*)-one (9)

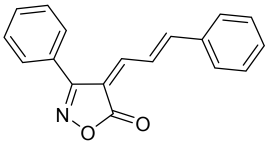

9

^{1}HNMR (0-10 ppm)
$CDCl_3$, 300 MHz

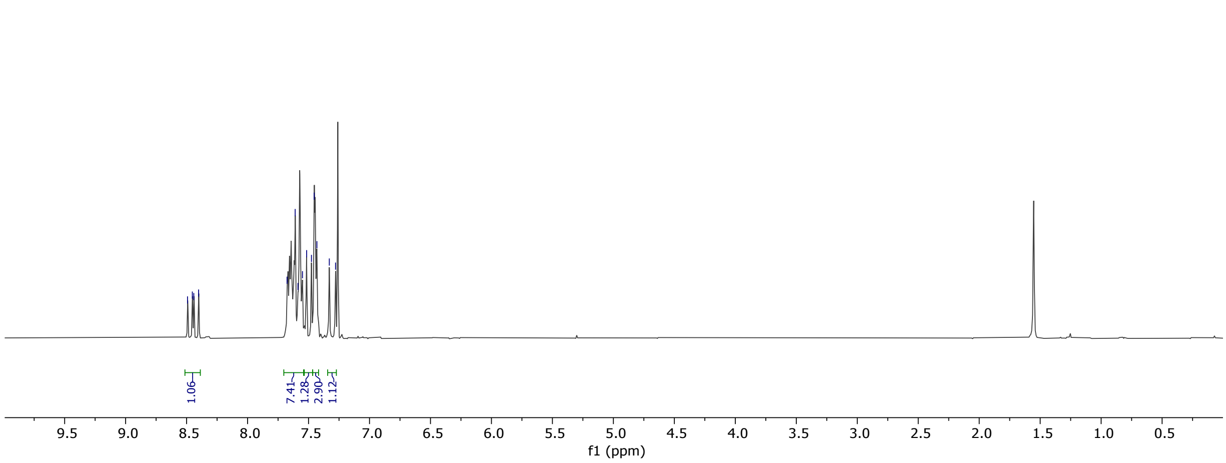

H^1-NMR and ^{13}C-NMR spectra of unknown compounds
(*Z*)-4-((Benzylamino)methylene)-3-phenylisoxazol-5(4*H*)-one (2a)

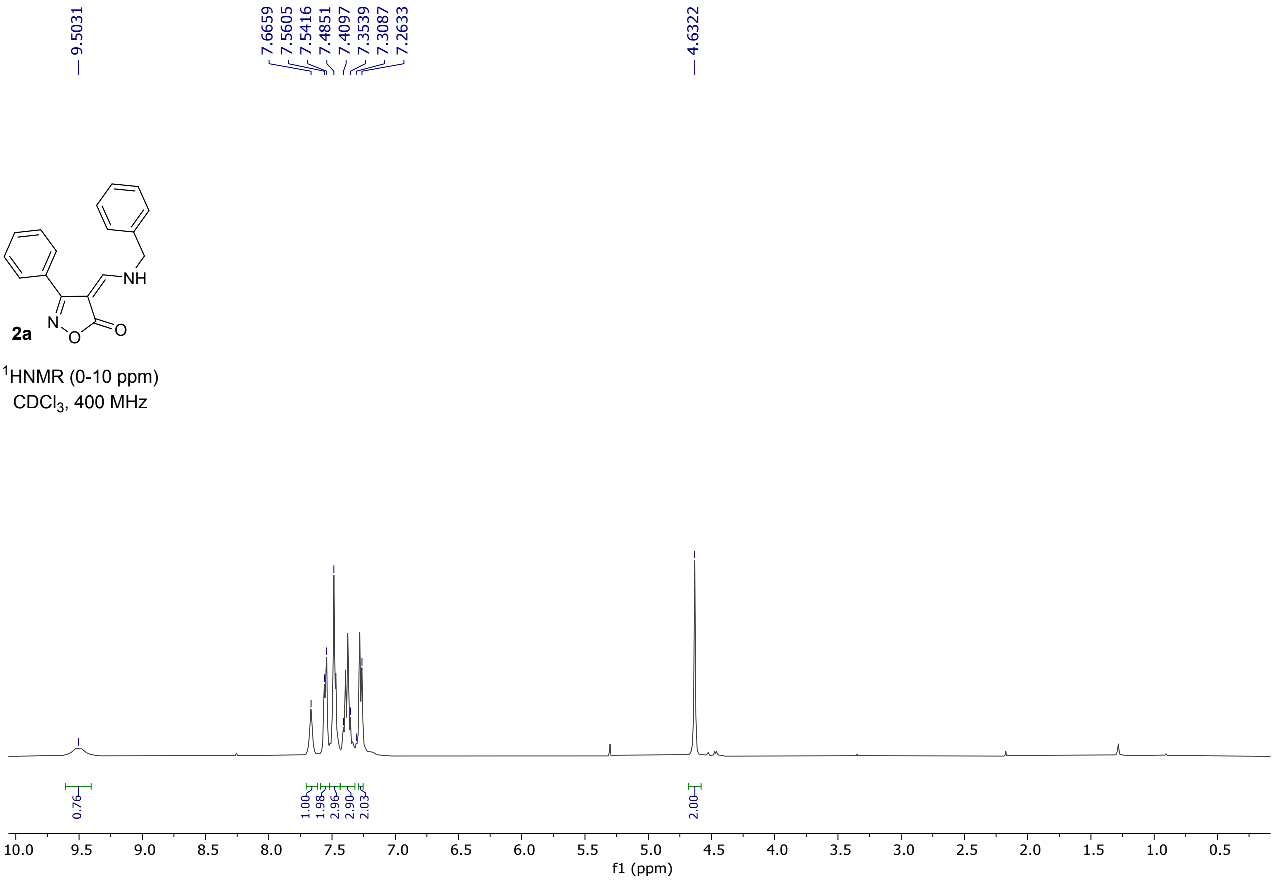

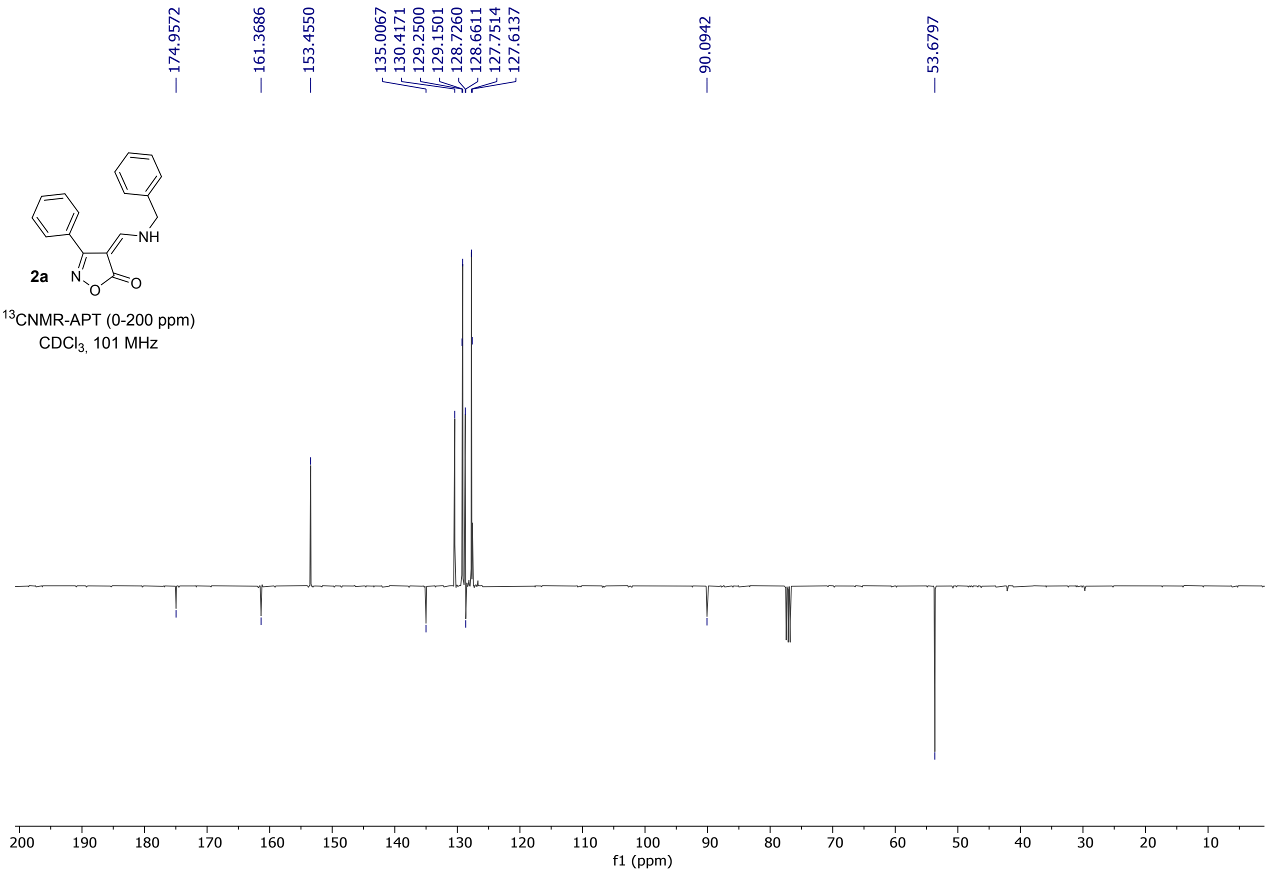

(*Z*)-3-Phenyl-4-((2-tolylamino)methylene)isoxazol-5(4*H*)-one (2c)

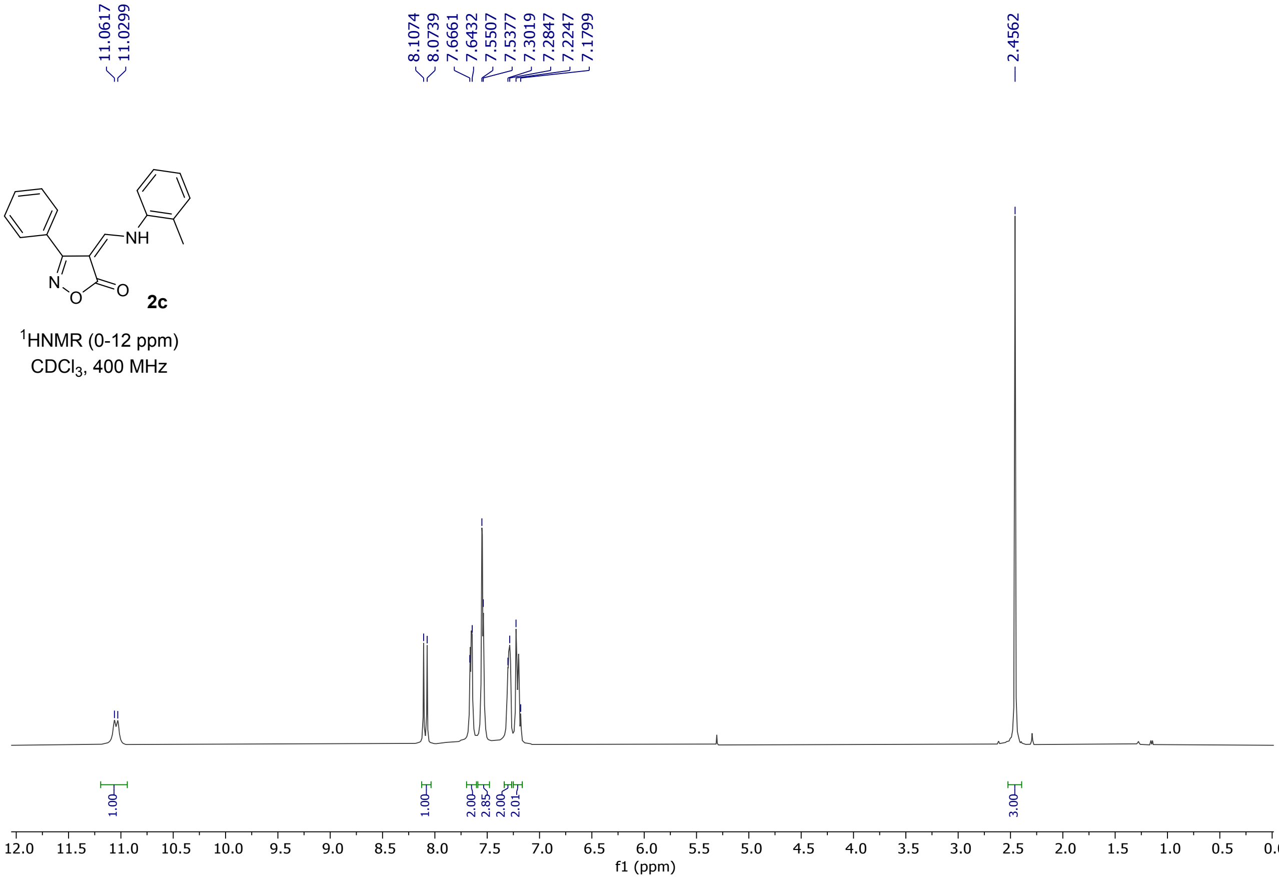

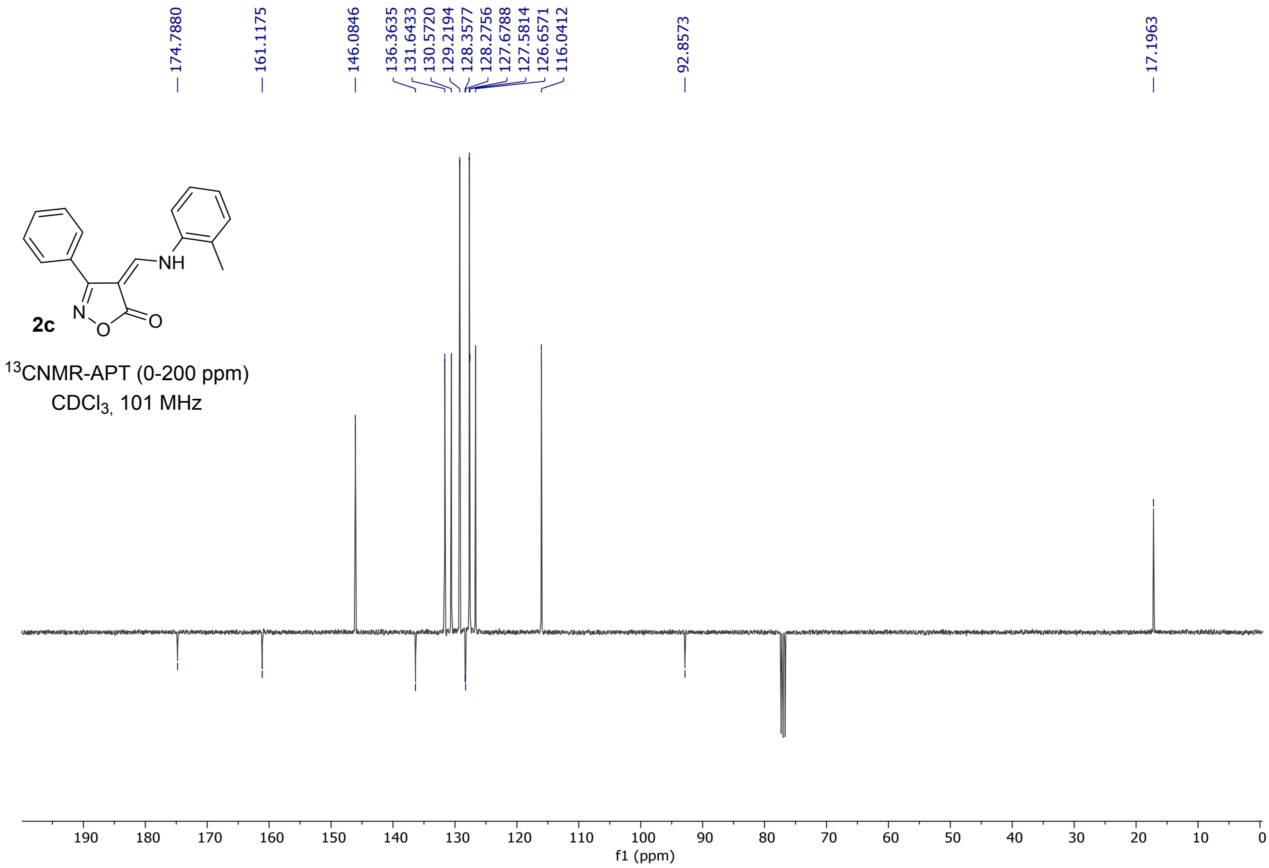

(Z)-4-(((2-Iodophenyl)amino)methylene)-3-phenylisoxazol-5(4*H*)-one (2d)

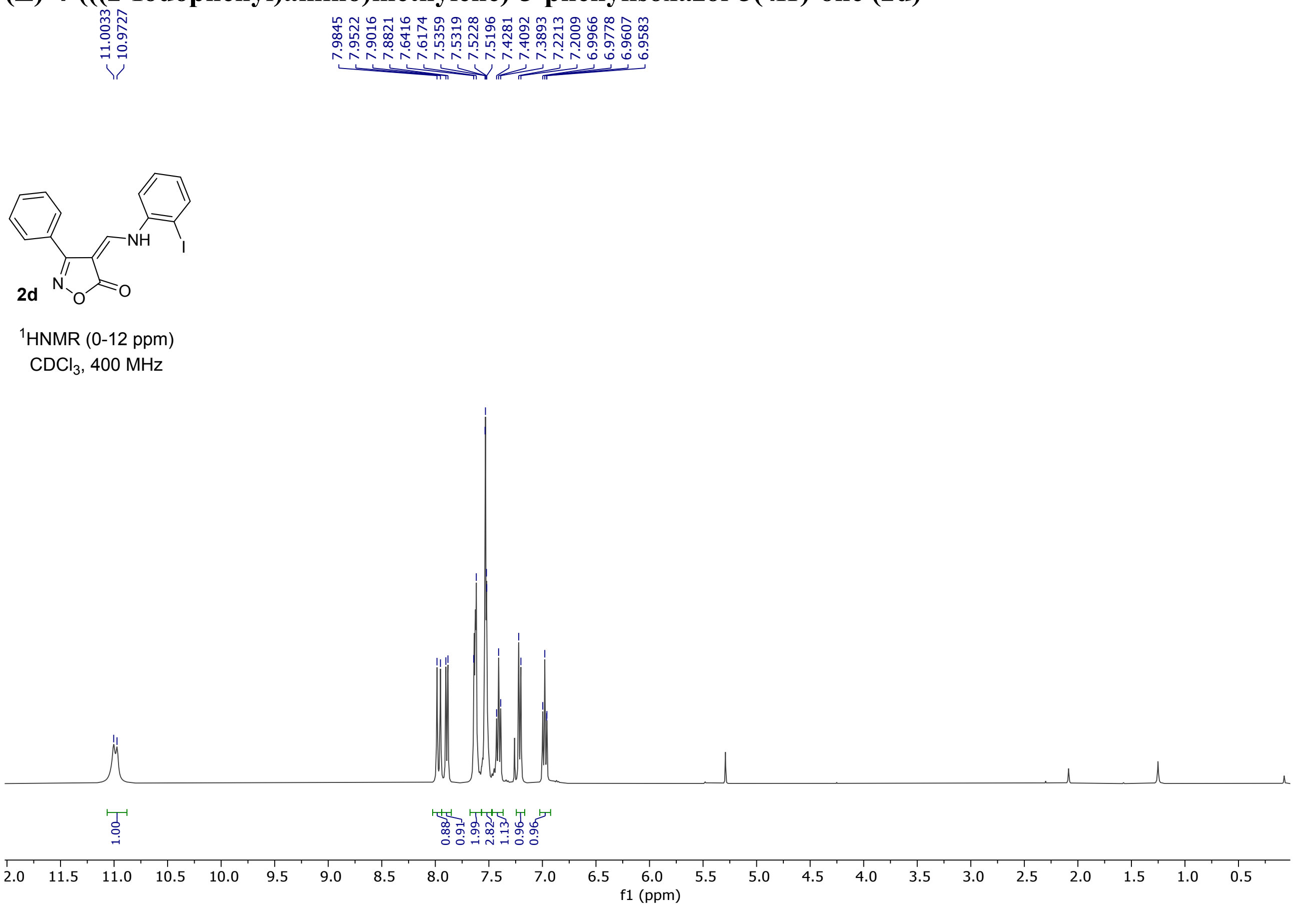

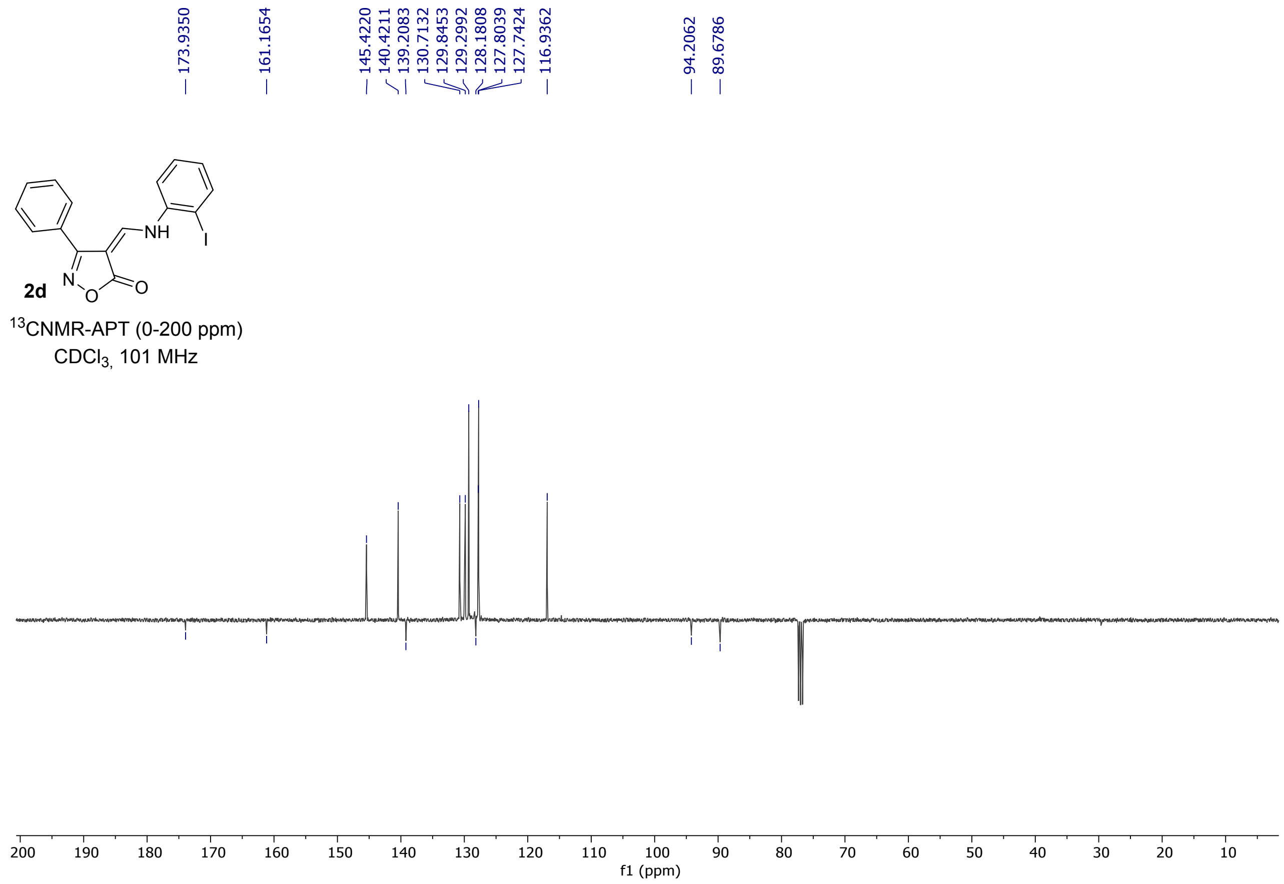

(*Z*)-4-(((4-Methoxyphenyl)amino)methylene)-3-phenylisoxazol-5(4*H*)-one (2e)

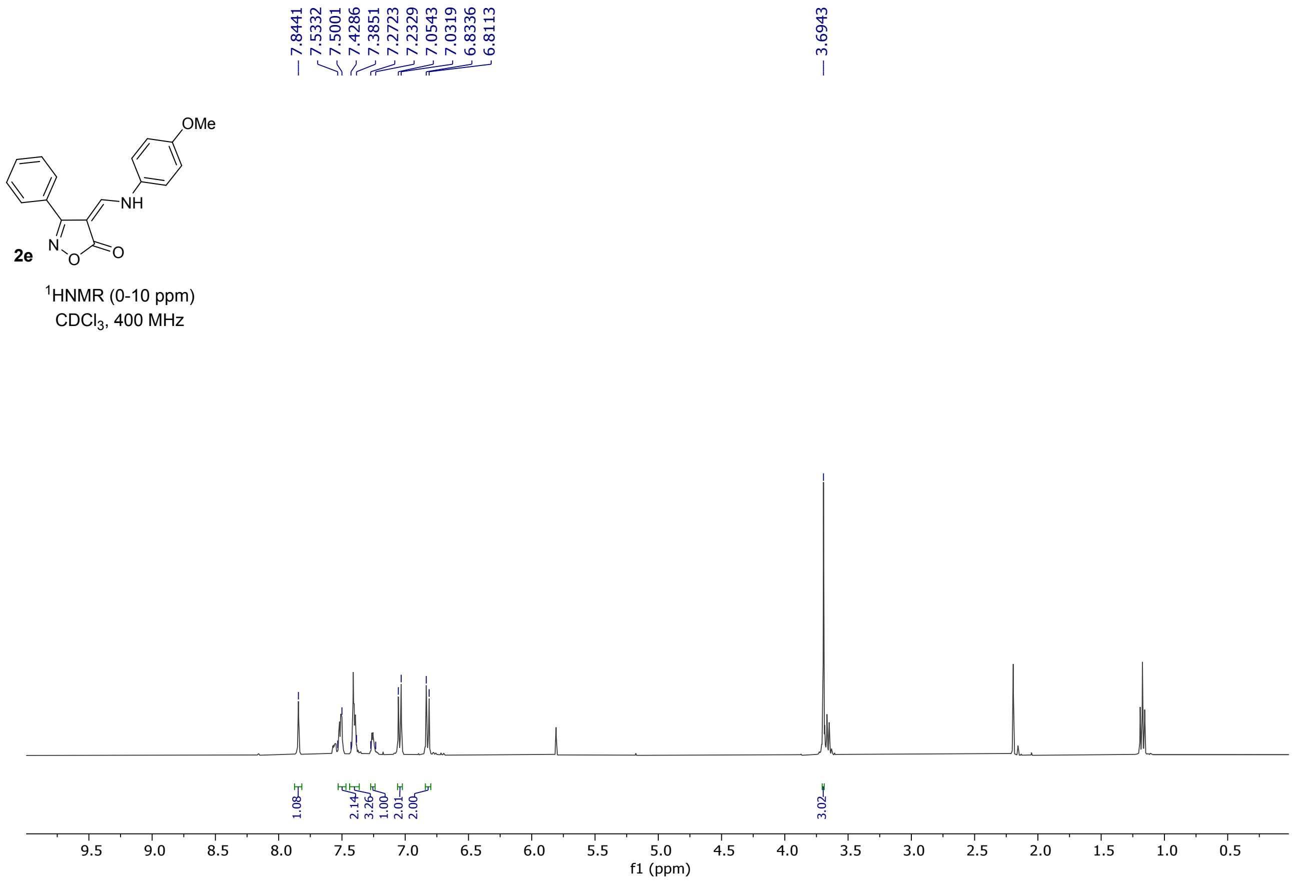

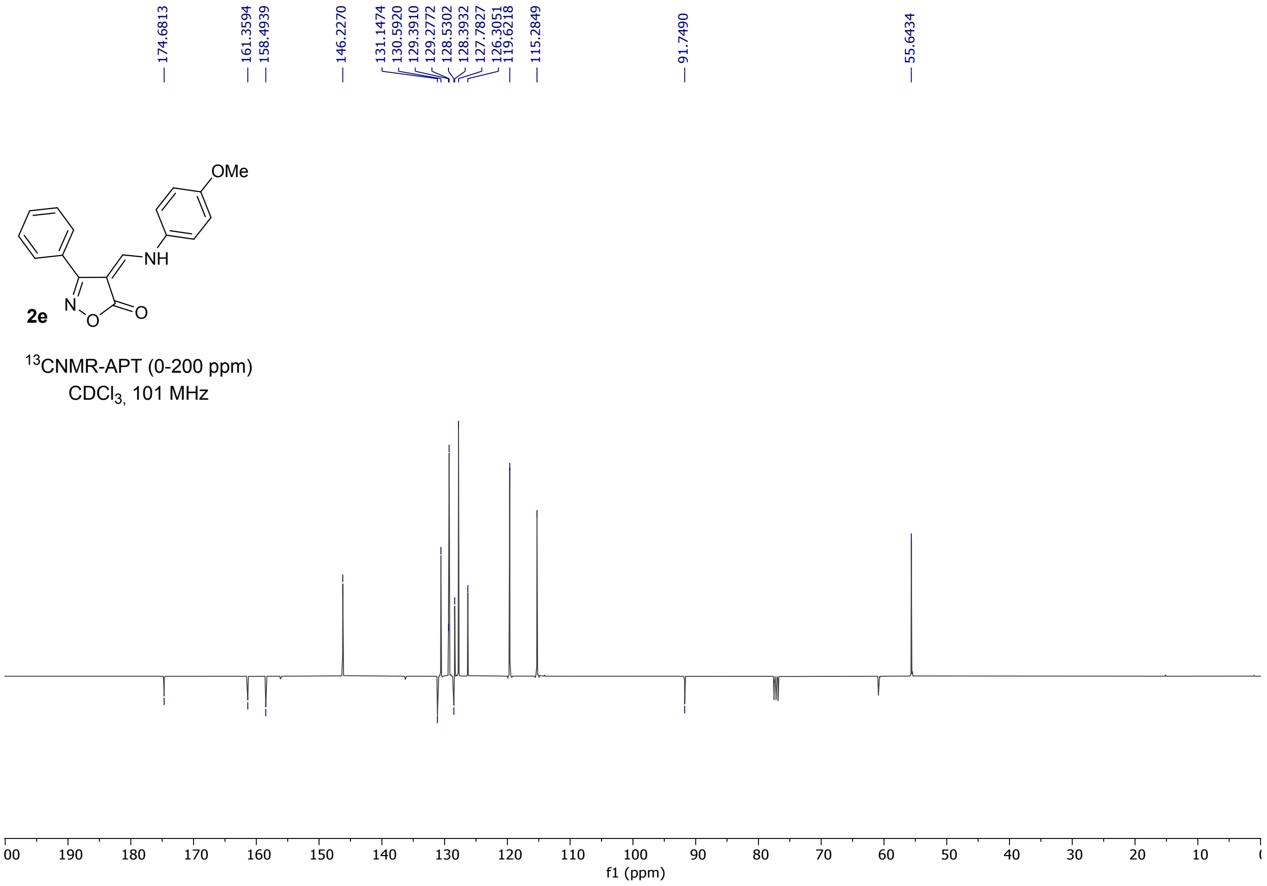

(*Z*)-4-((Phenylamino)methylene)-3-propylisoxazol-5(4*H*)-one (2f)

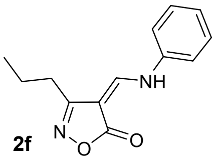

2f

^{1}HNMR (0-10 ppm)
$CDCl_3$, 400 MHz

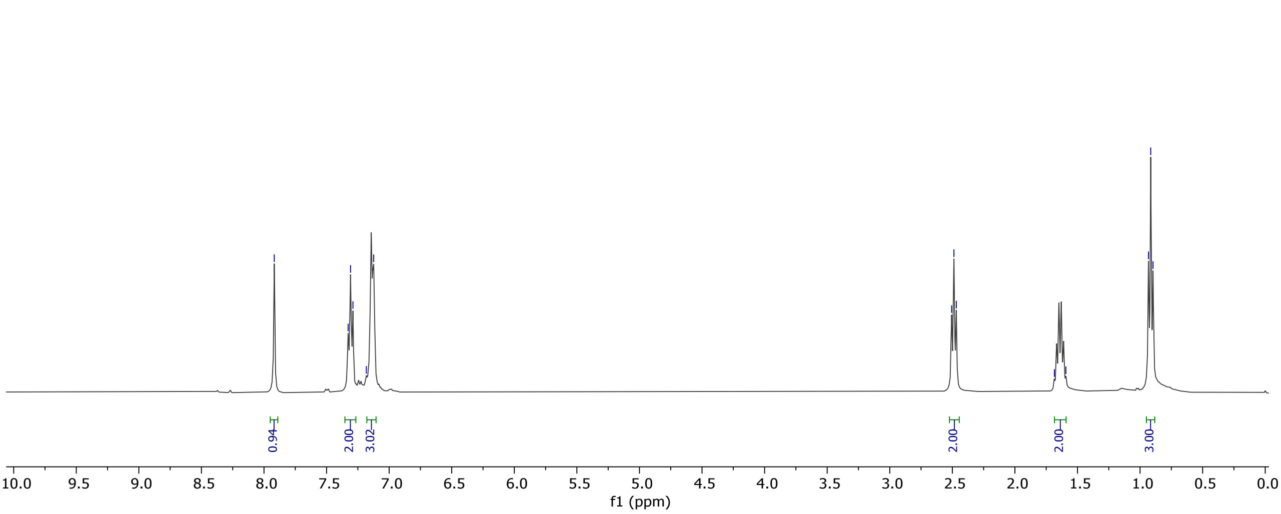

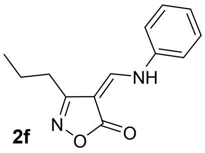

2f

^{13}CNMR-APT (0-200 ppm)
$CDCl_3$, 101 MHz

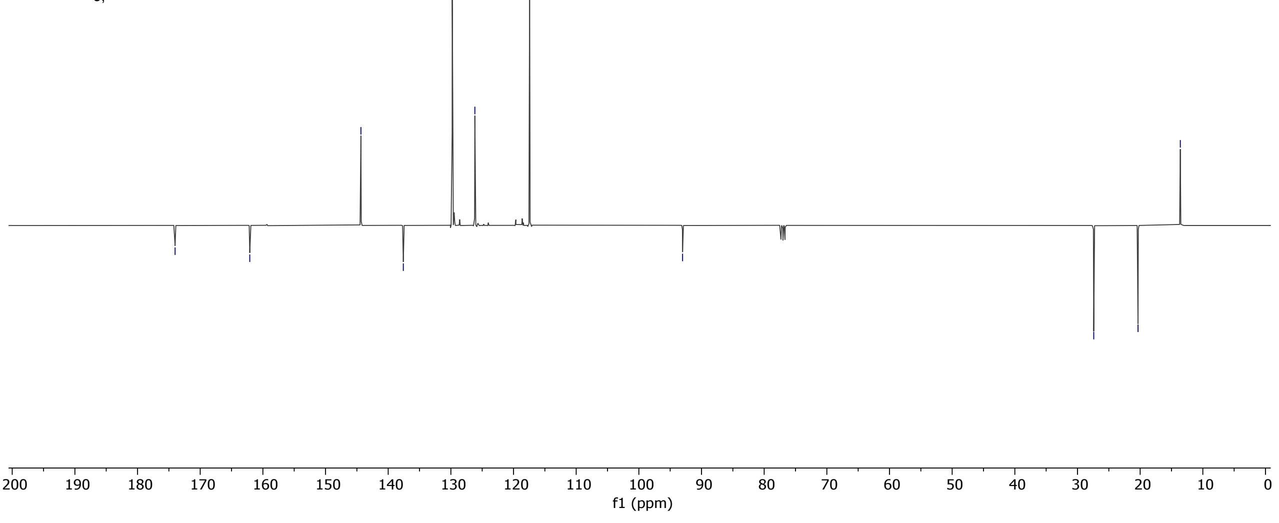

(Z)-3-Propyl-4-((4-tolylamino)methylene)isoxazol-5(4H)-one (2g)

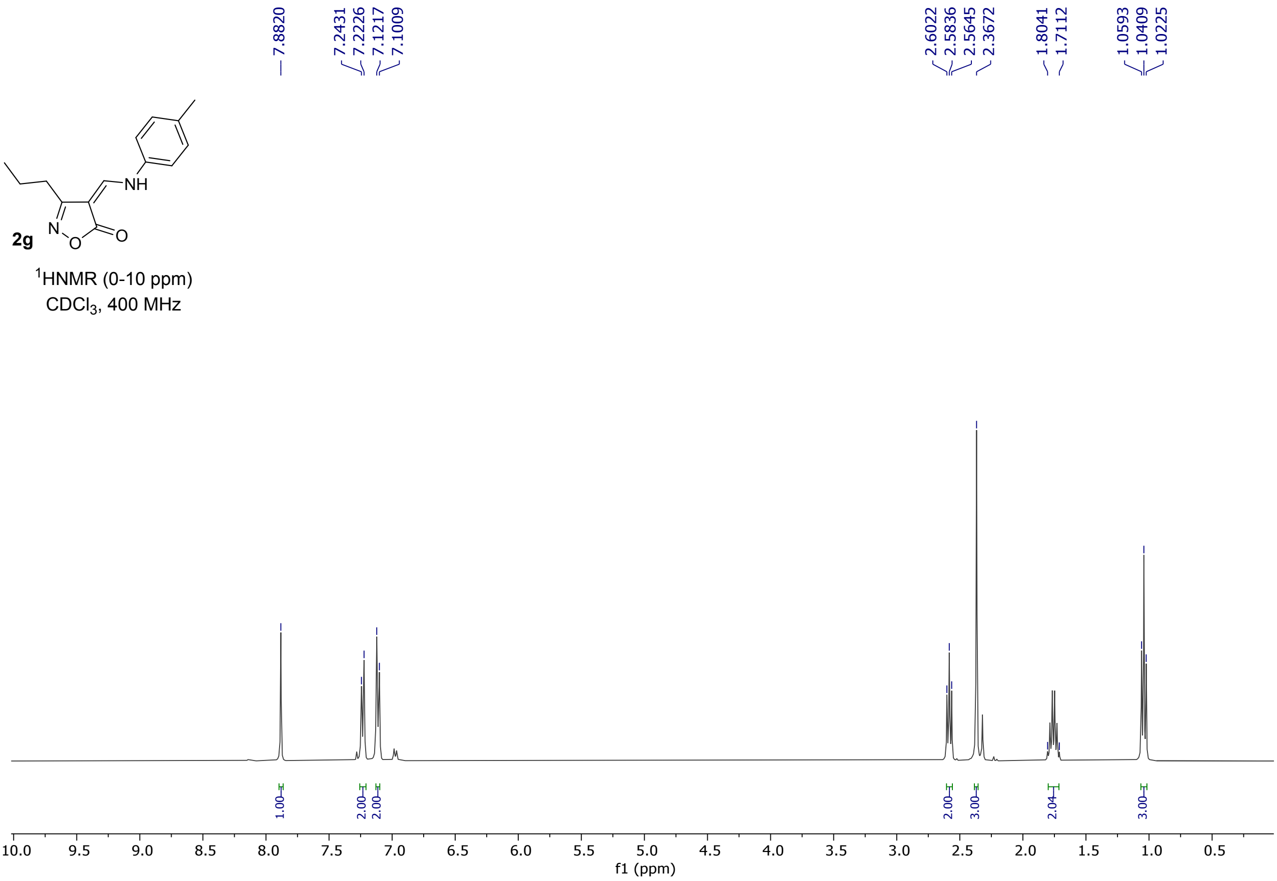

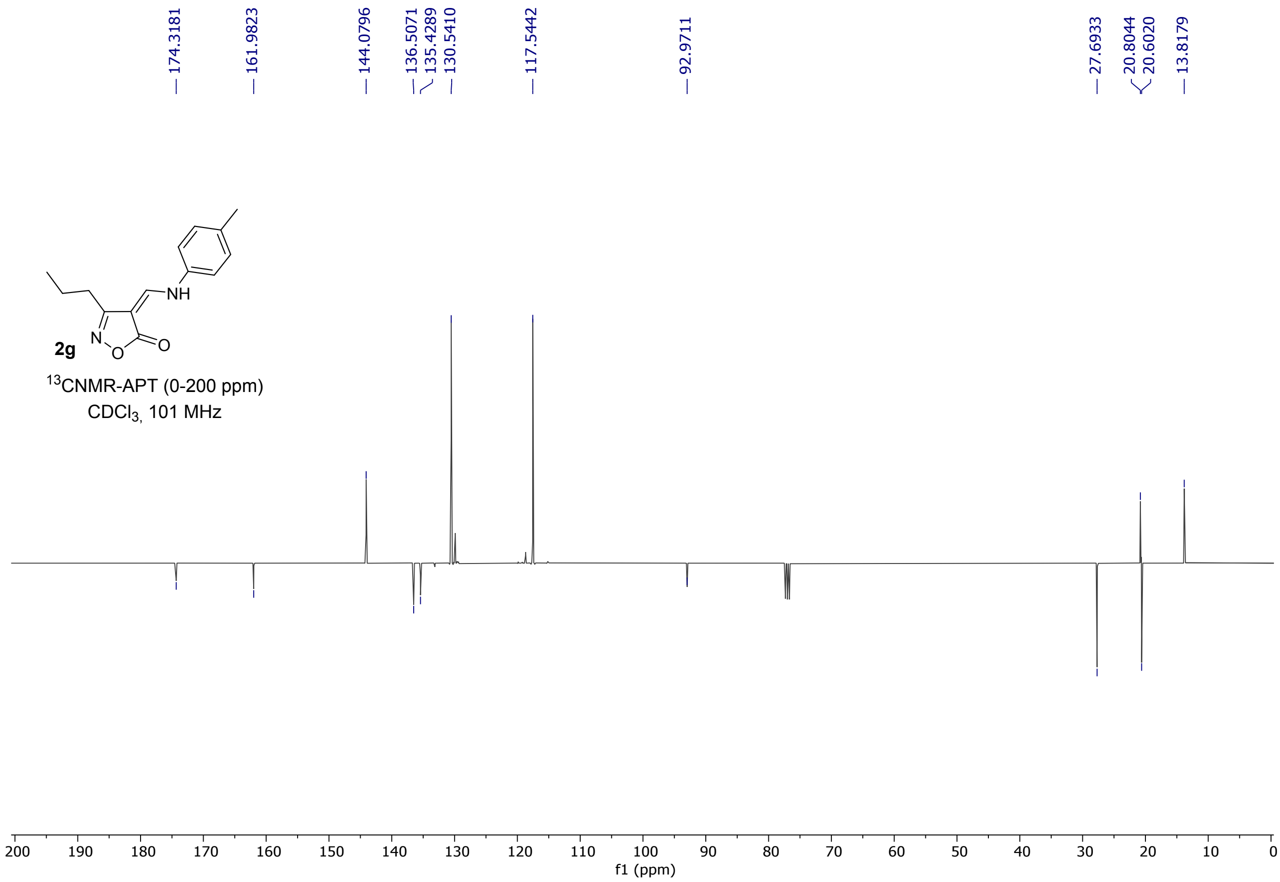

(*Z*)-4-(((4-Methoxyphenyl)amino)methylene)-3-propylisoxazol-5(4*H*)-one (2h)

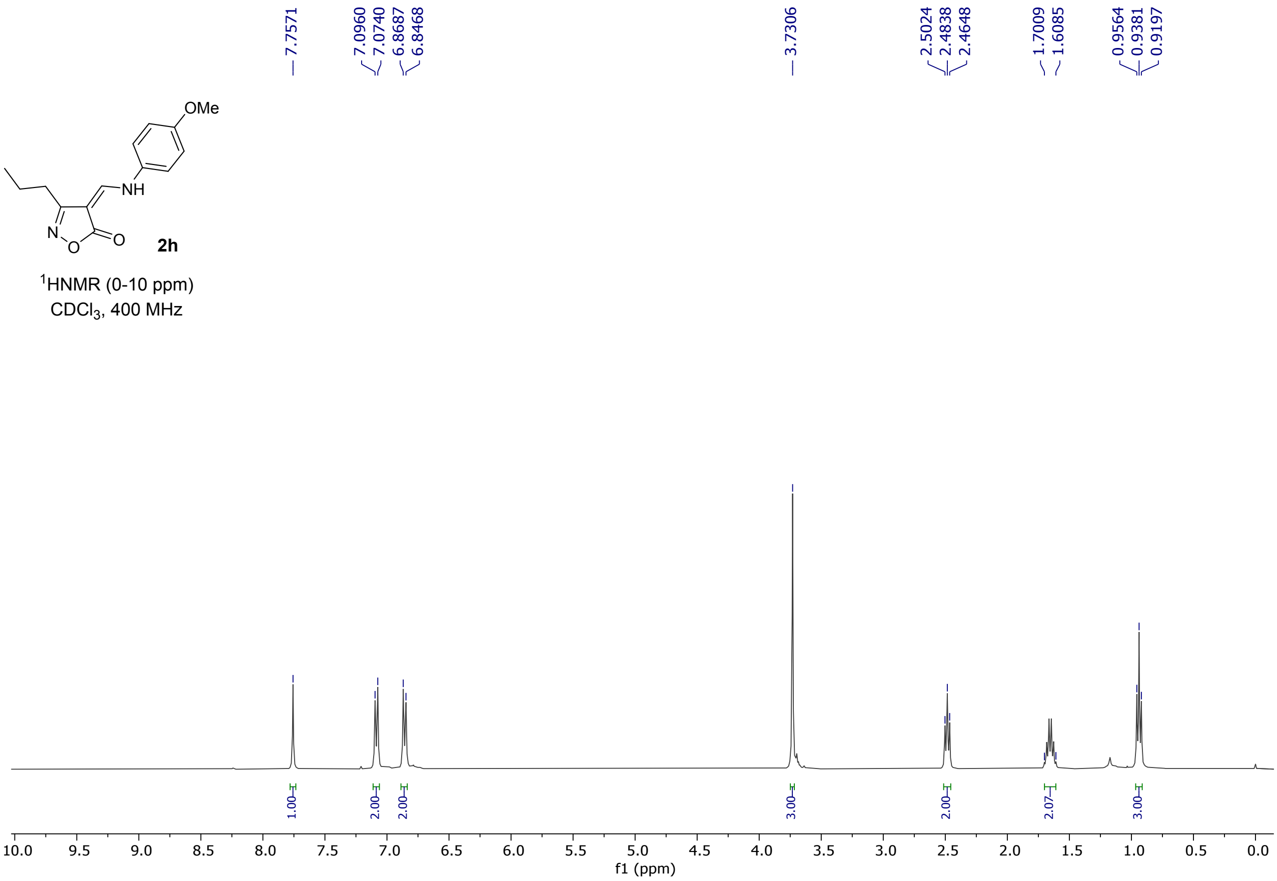

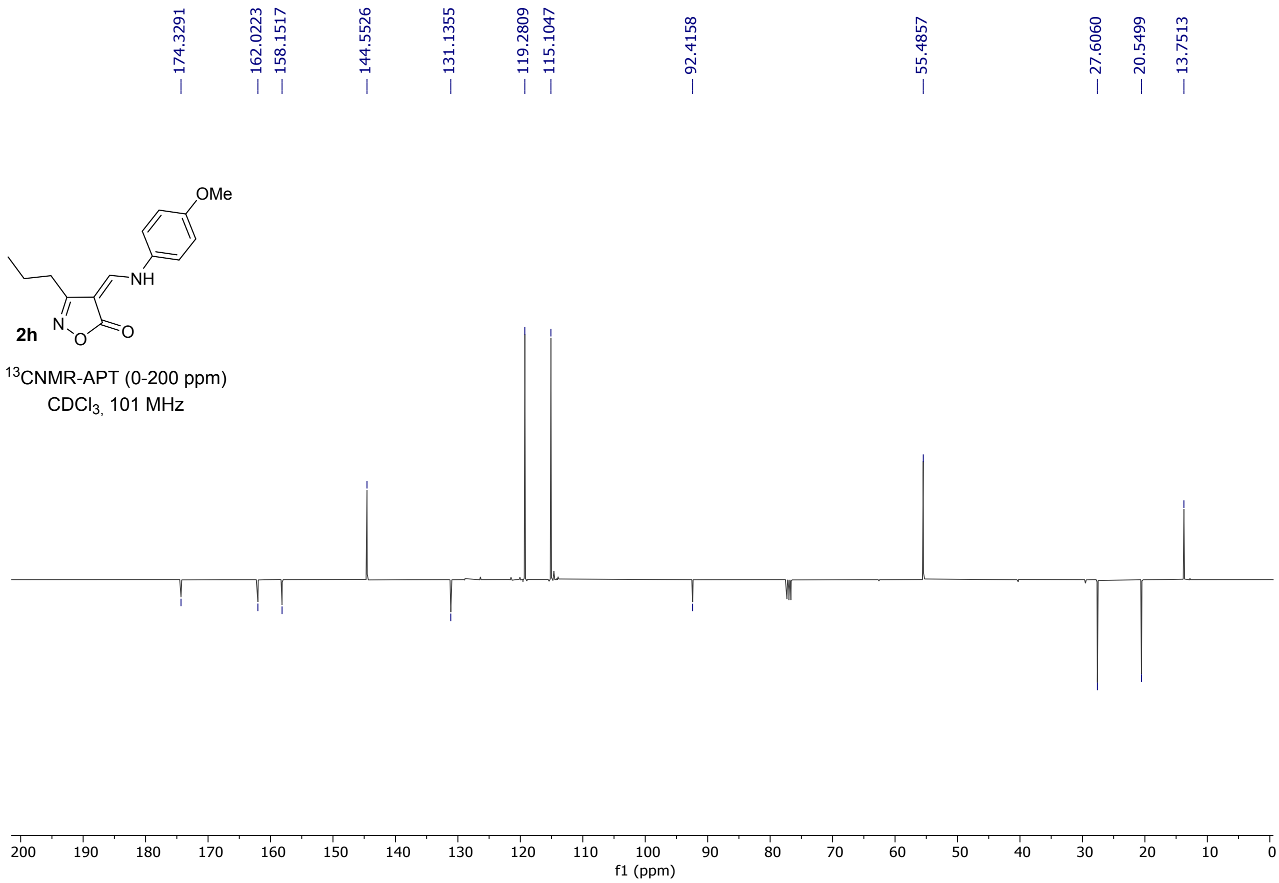

(*Z*)-3-Propyl-4-((3-tolylamino)methylene)isoxazol-5(4*H*)-one (2i)

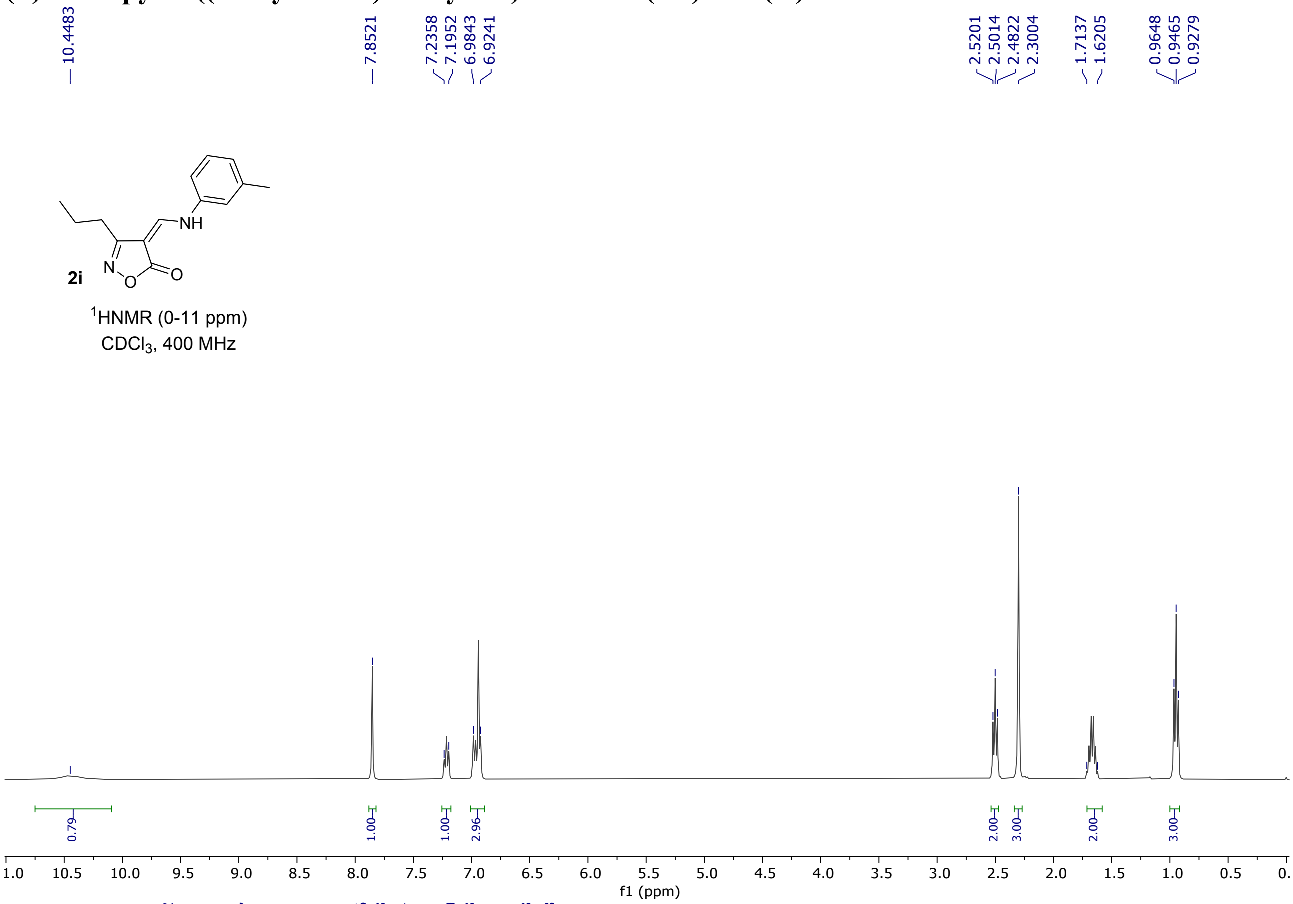

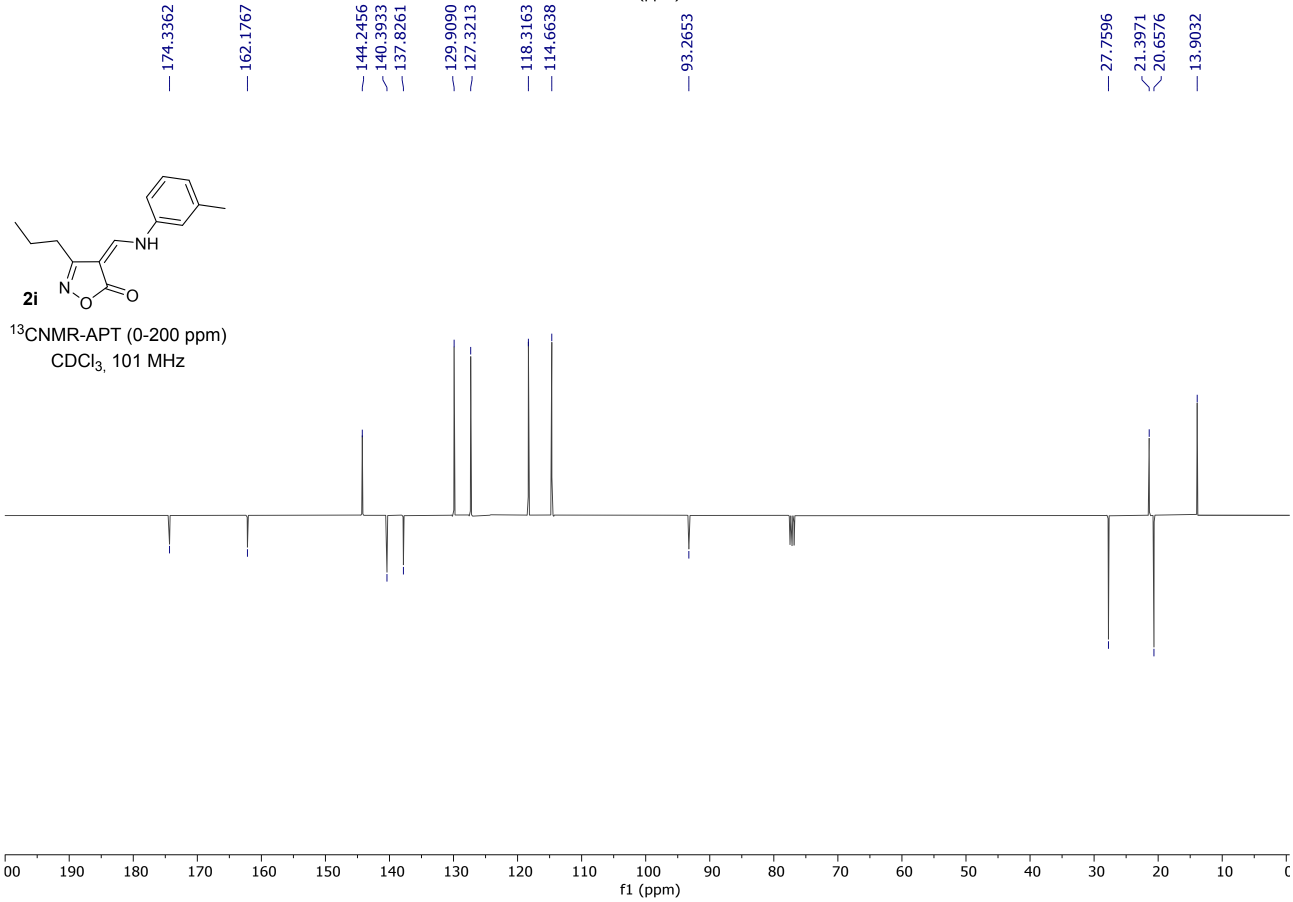

(*Z*)-4-((Phenethylamino)methylene)-3-propylisoxazol-5(4*H*)-one (2j)

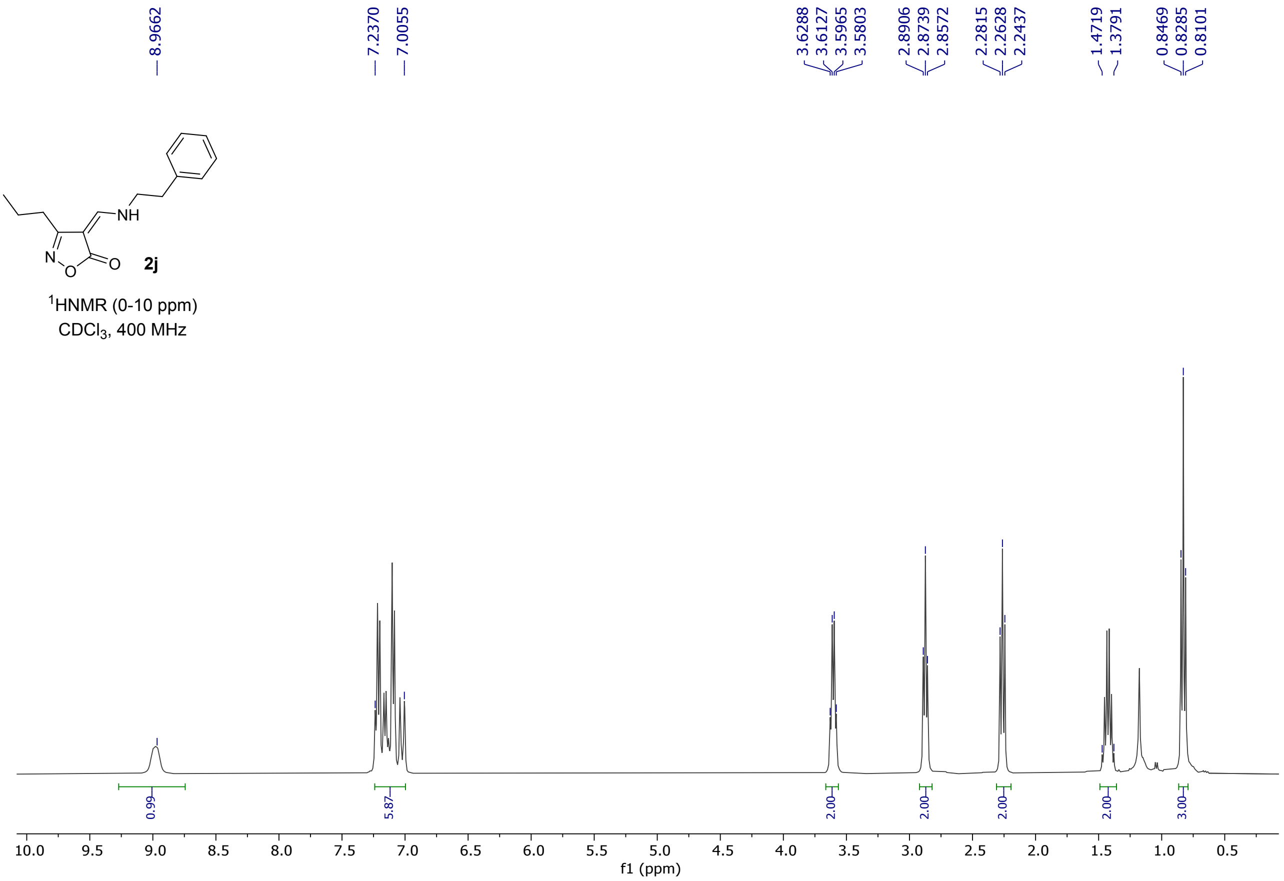

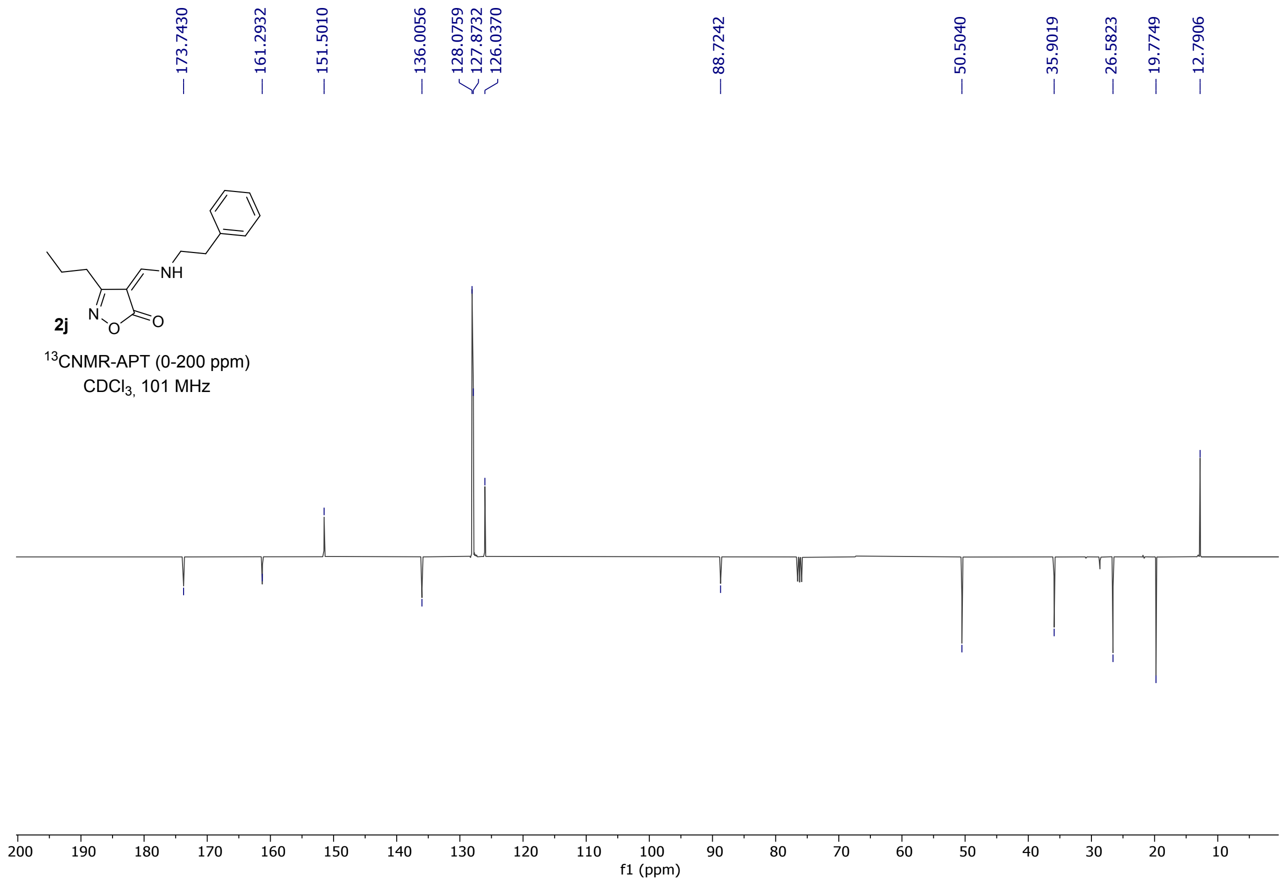

4-((Butylamino)methylene)-3-methylisoxazol-5(4*H*)-one (2k)

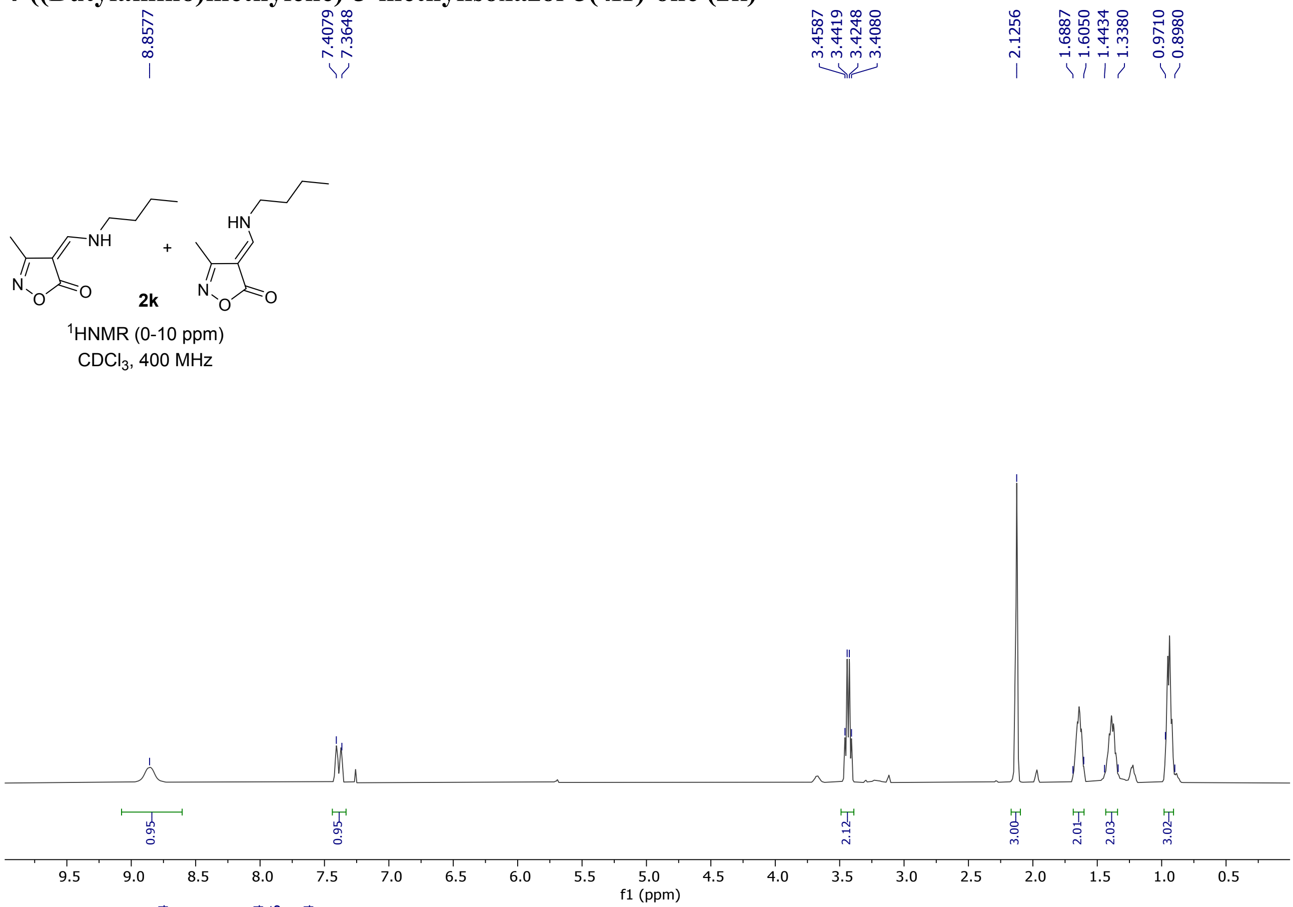

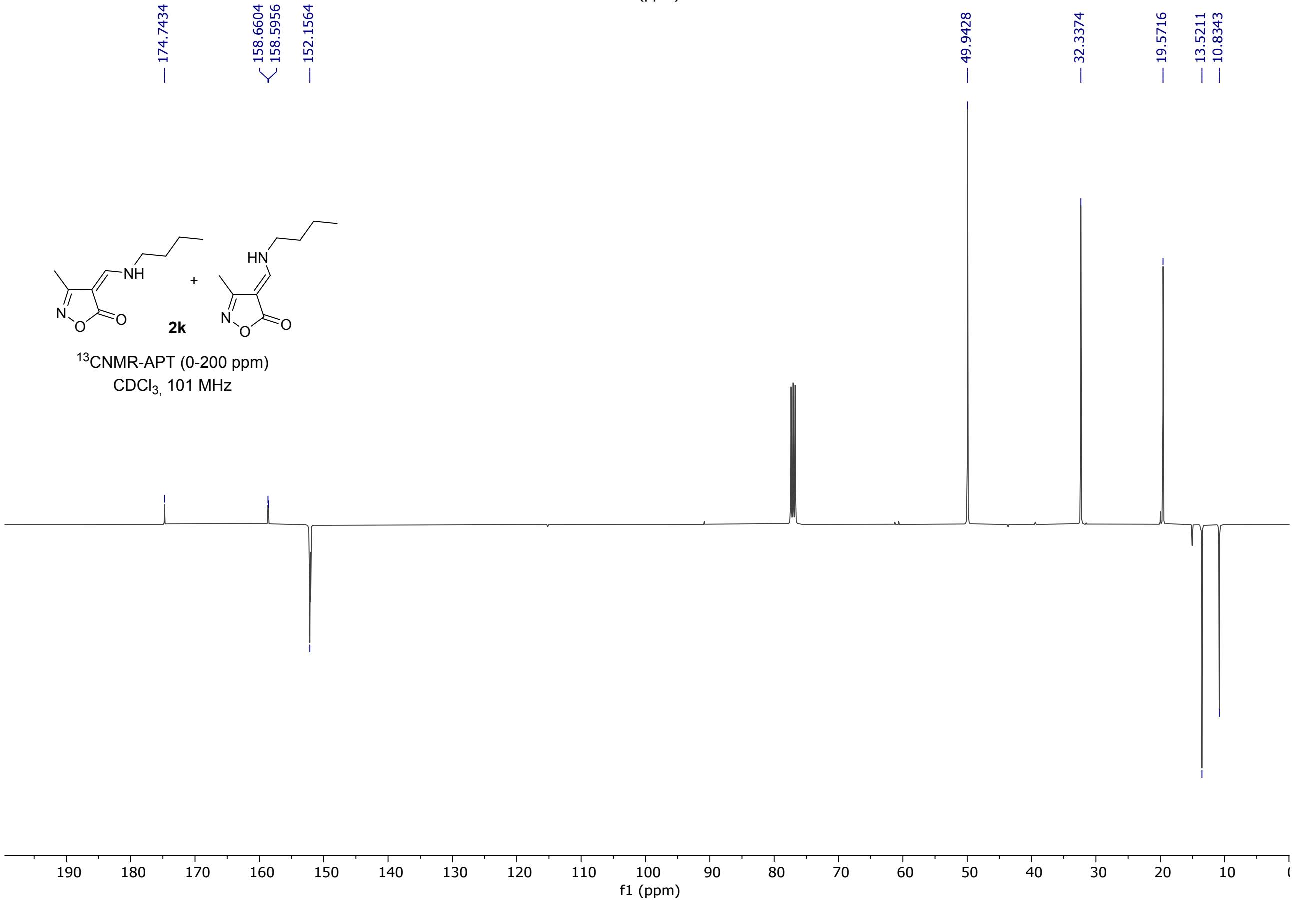

(*Z*)-4-(Phenyl(phenylamino)methylene)-3-propylisoxazol-5(4*H*)-one (2l)

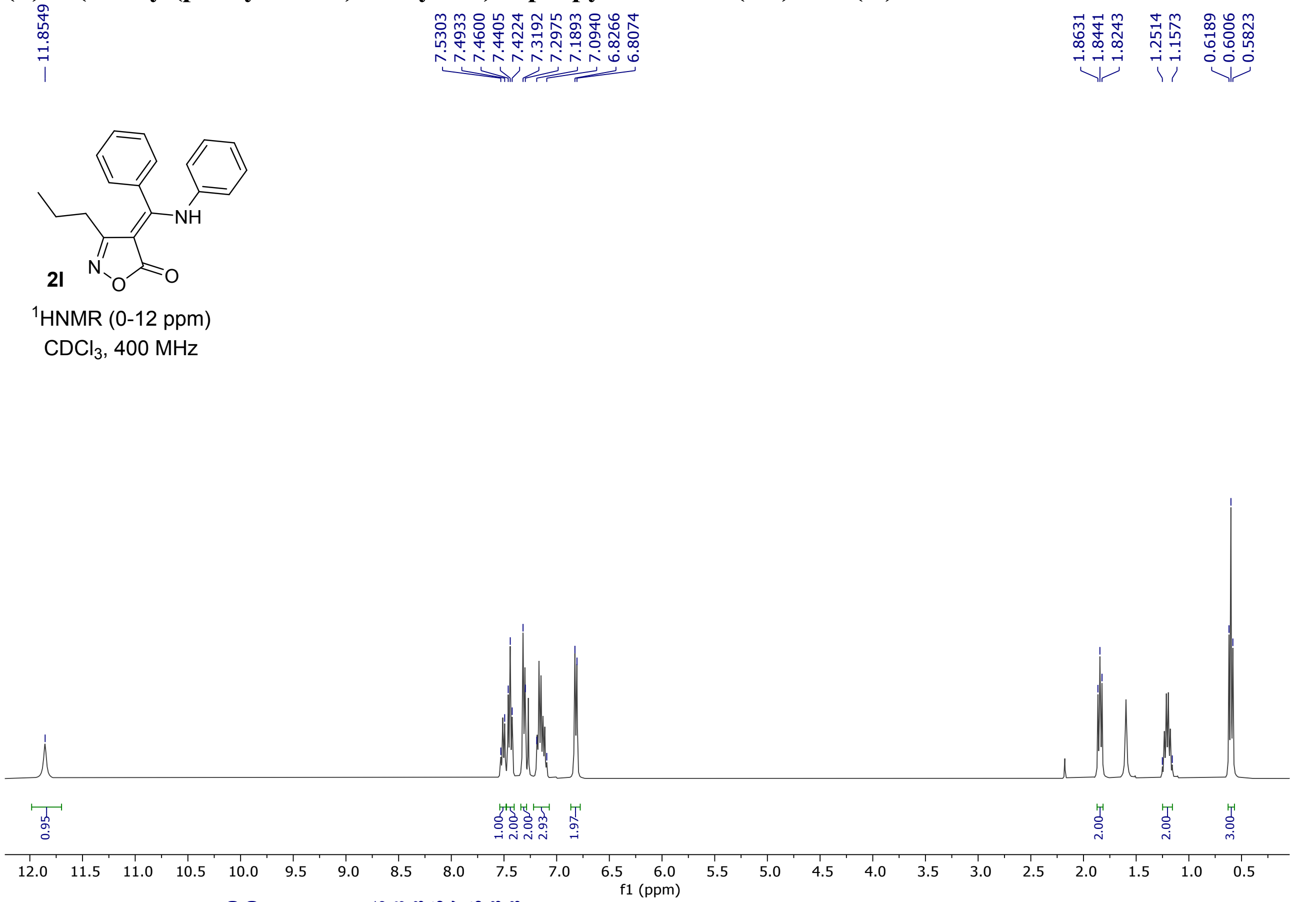

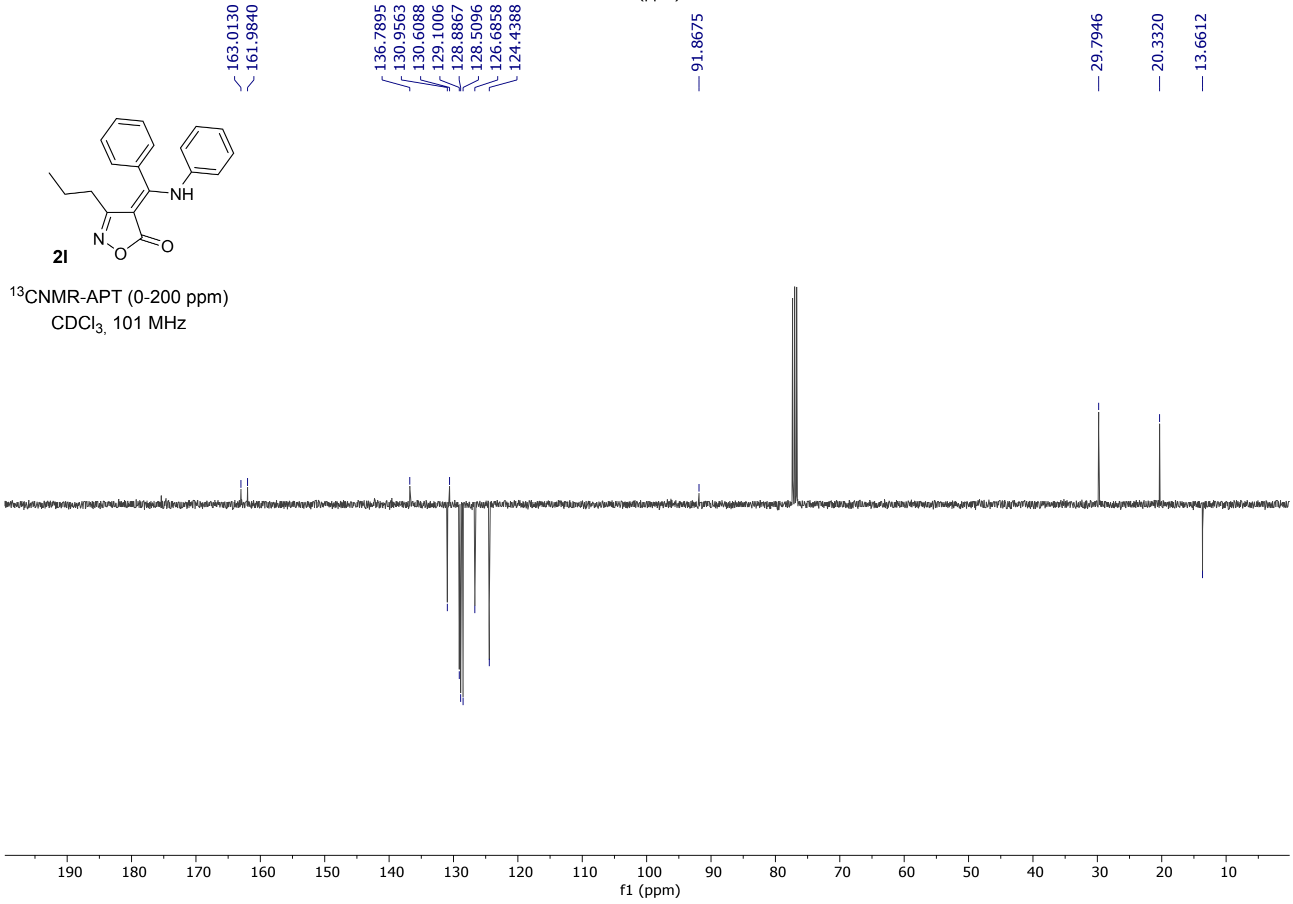

(*Z*)-4-(1-(Benzylamino)ethylidene)-3-propylisoxazol-5(4*H*)-one (2m)

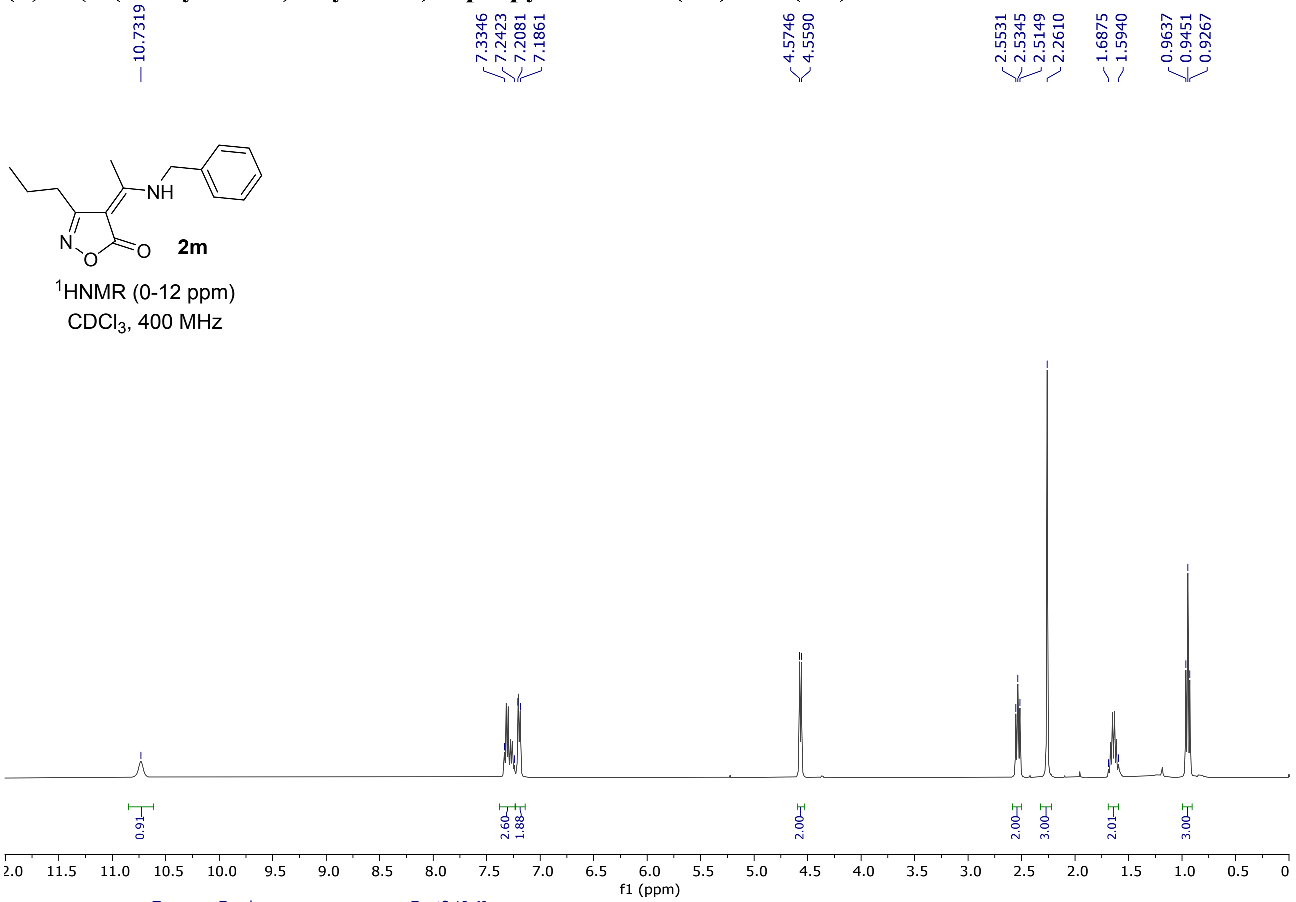

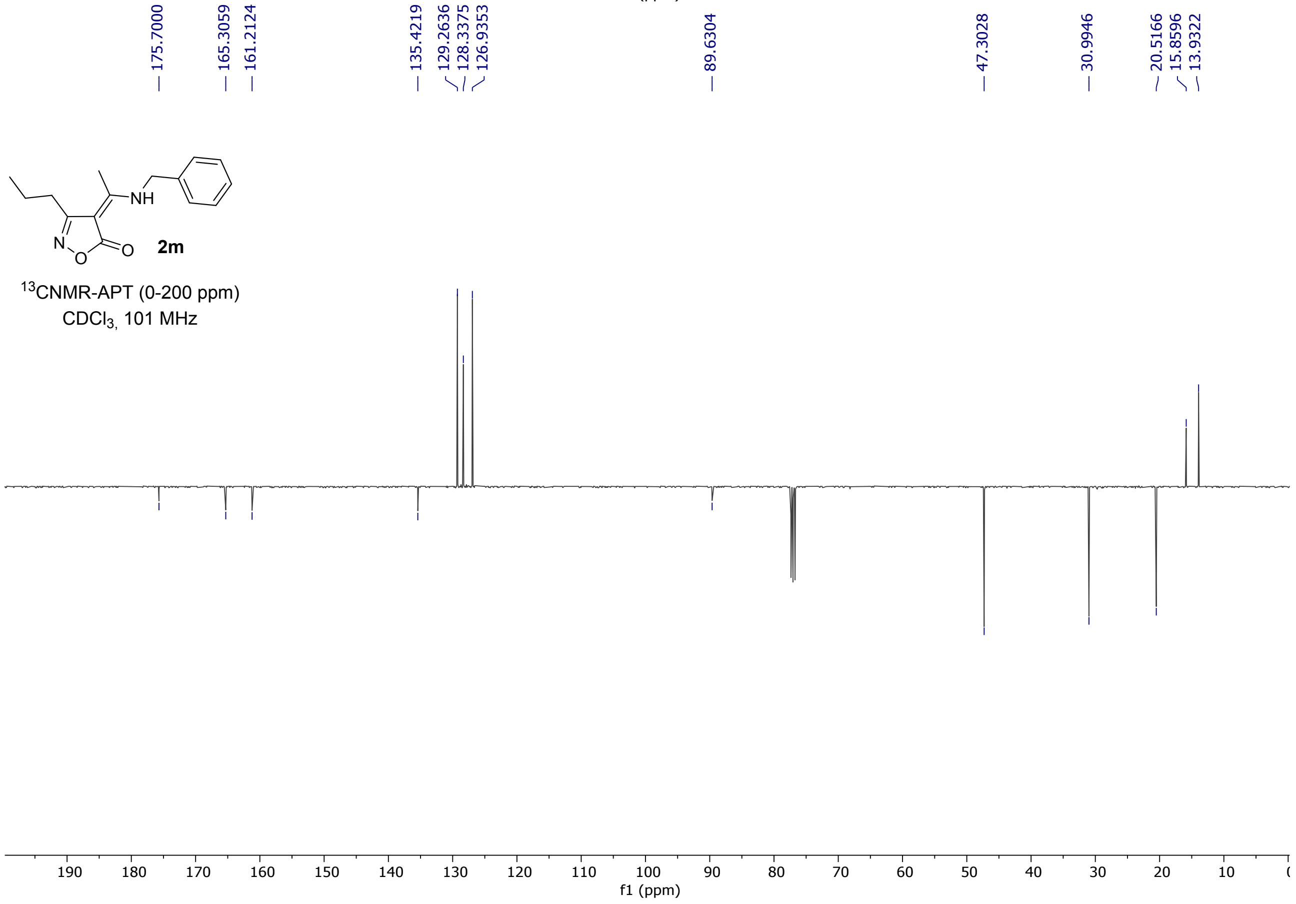

(*Z*)-4-(1-(Benzylamino)propylidene)-3-methylisoxazol-5(4*H*)-one (2n)

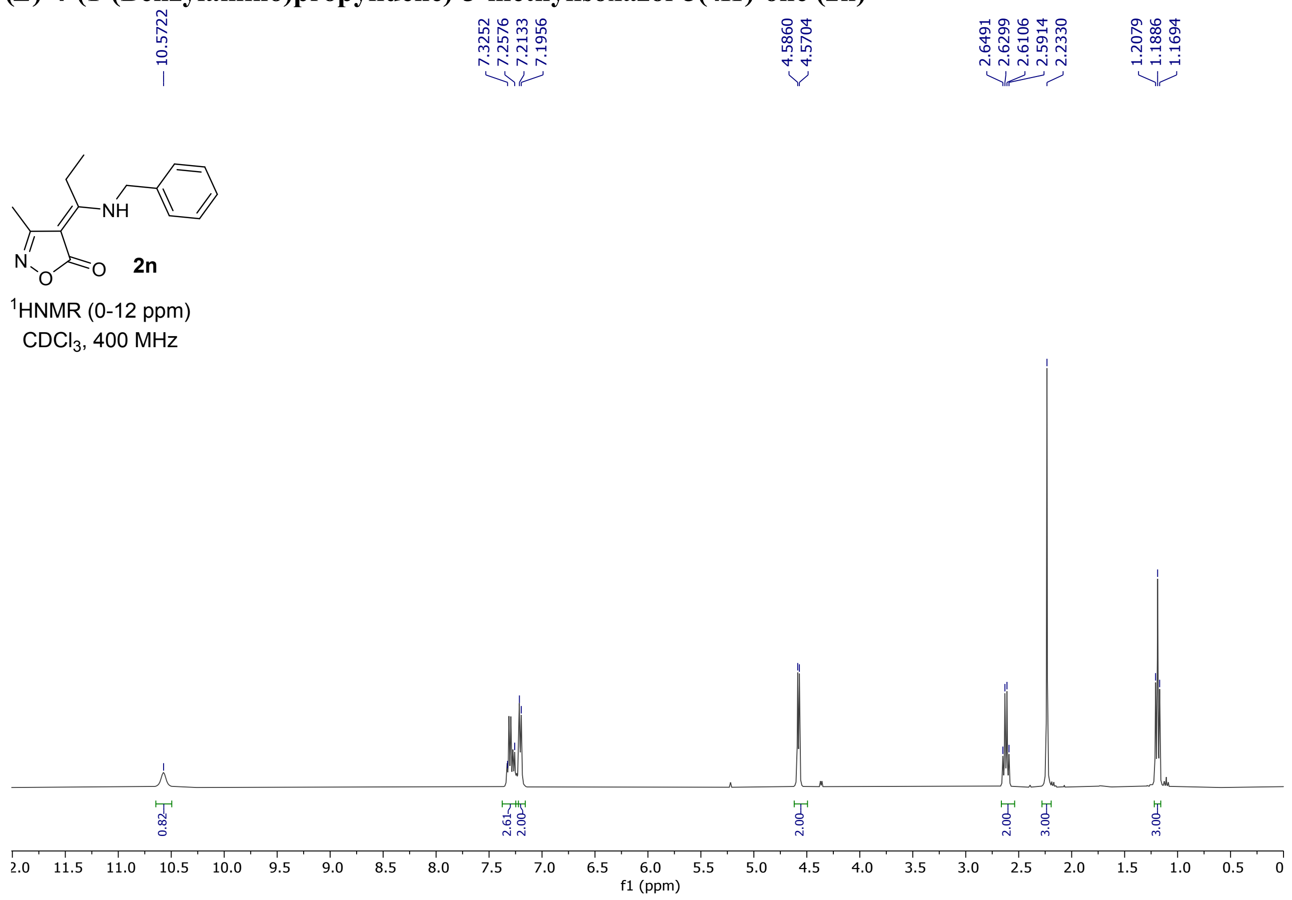

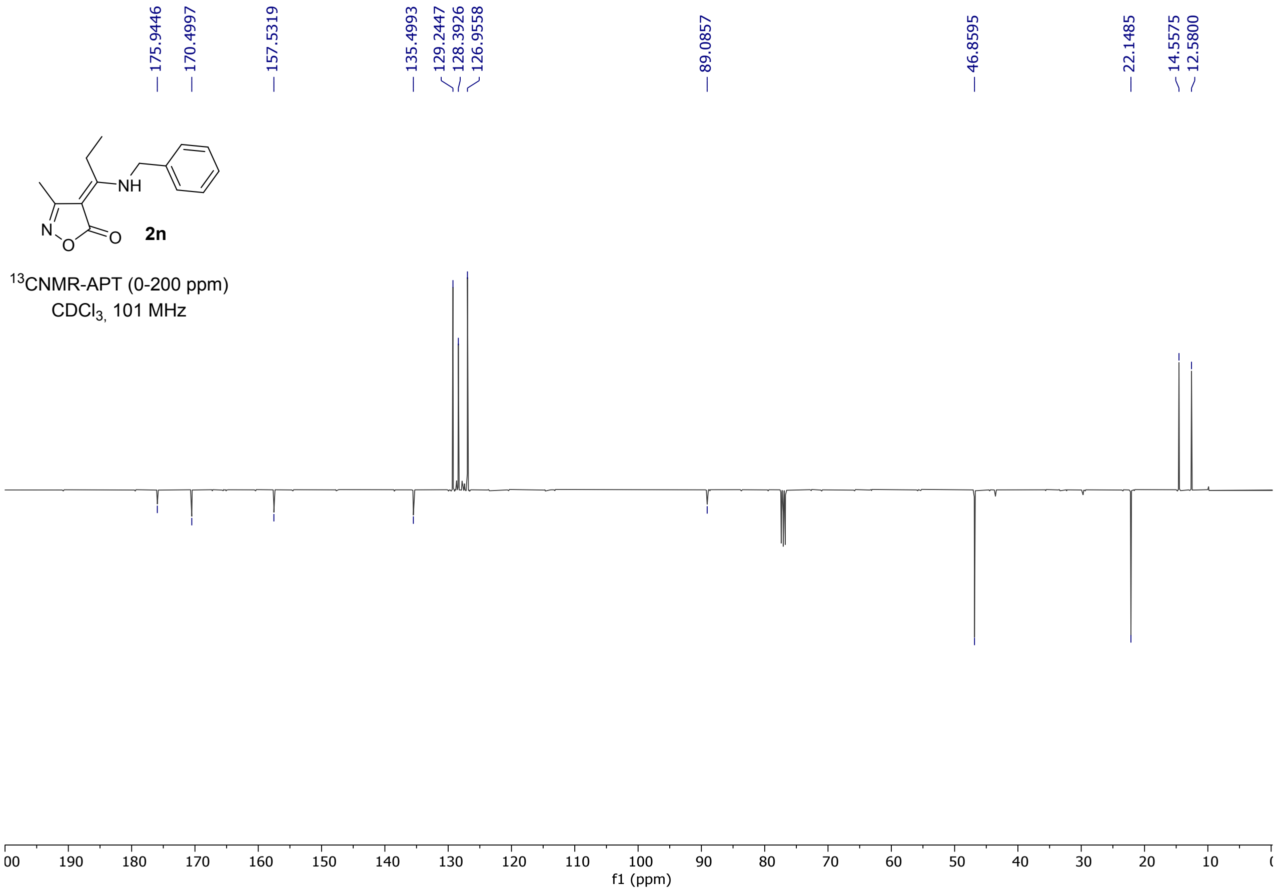

(*Z*)-4-(1-(Benzylamino)propylidene)-3-propylisoxazol-5(4*H*)-one (2o)

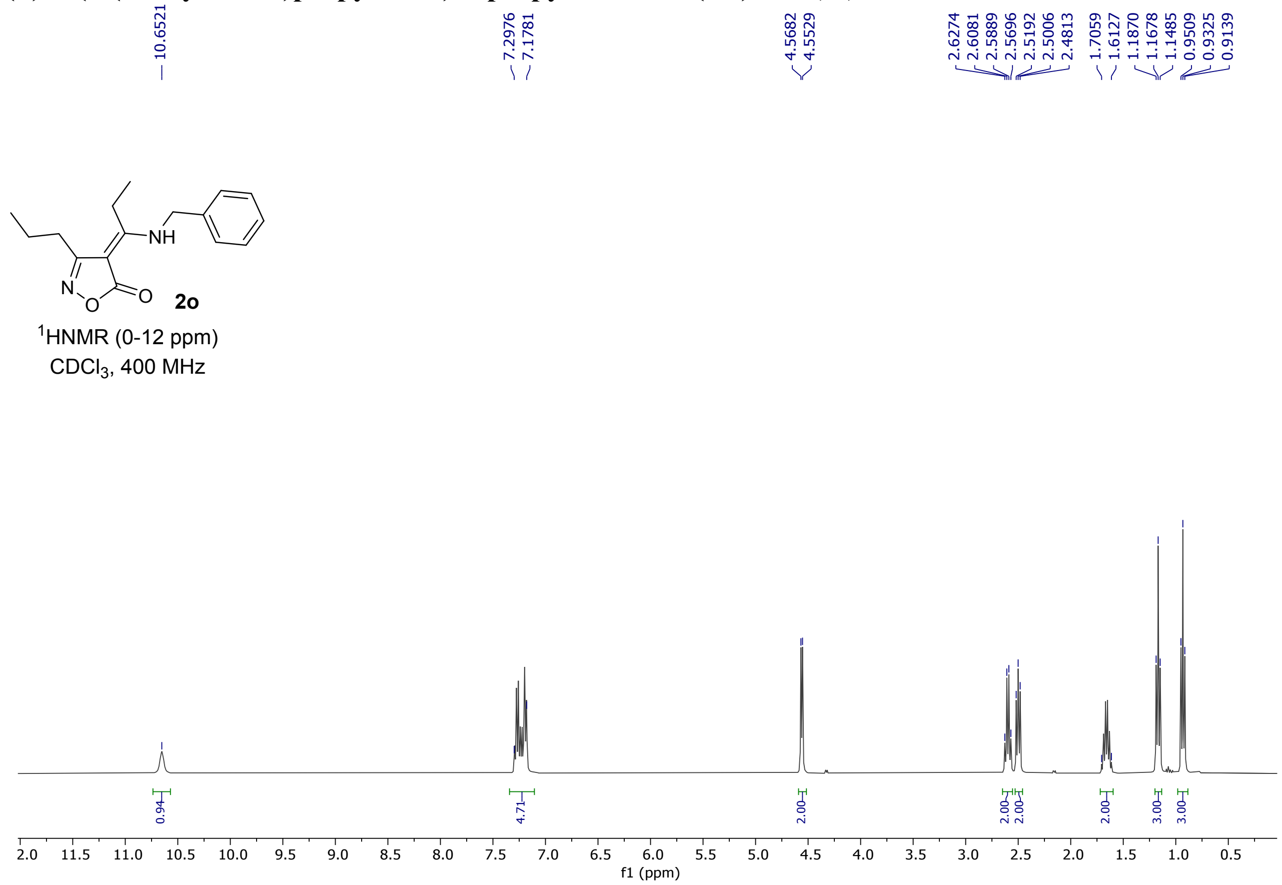

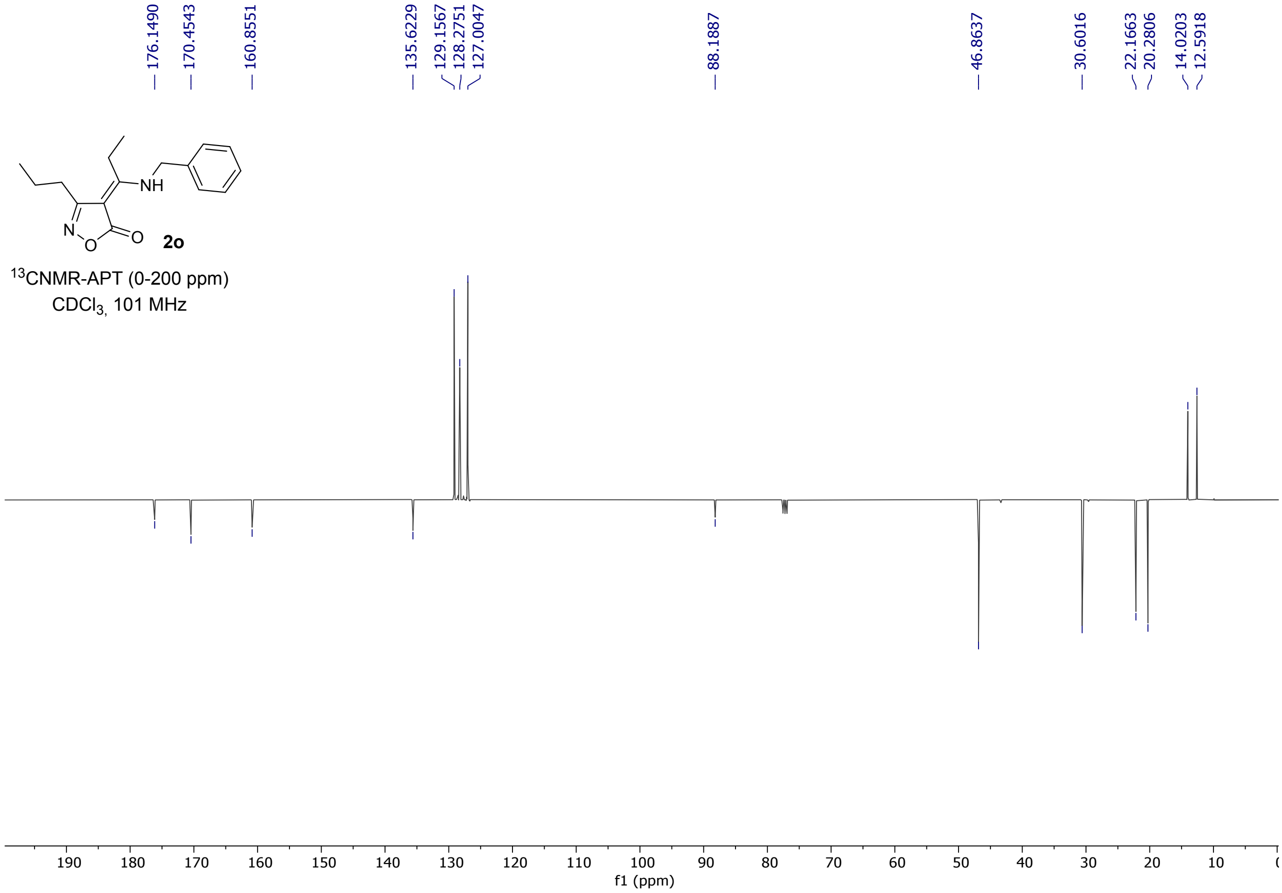

(*Z*)-4-(1-Aminopropylidene)-3-propylisoxazol-5(4*H*)-one (2p)

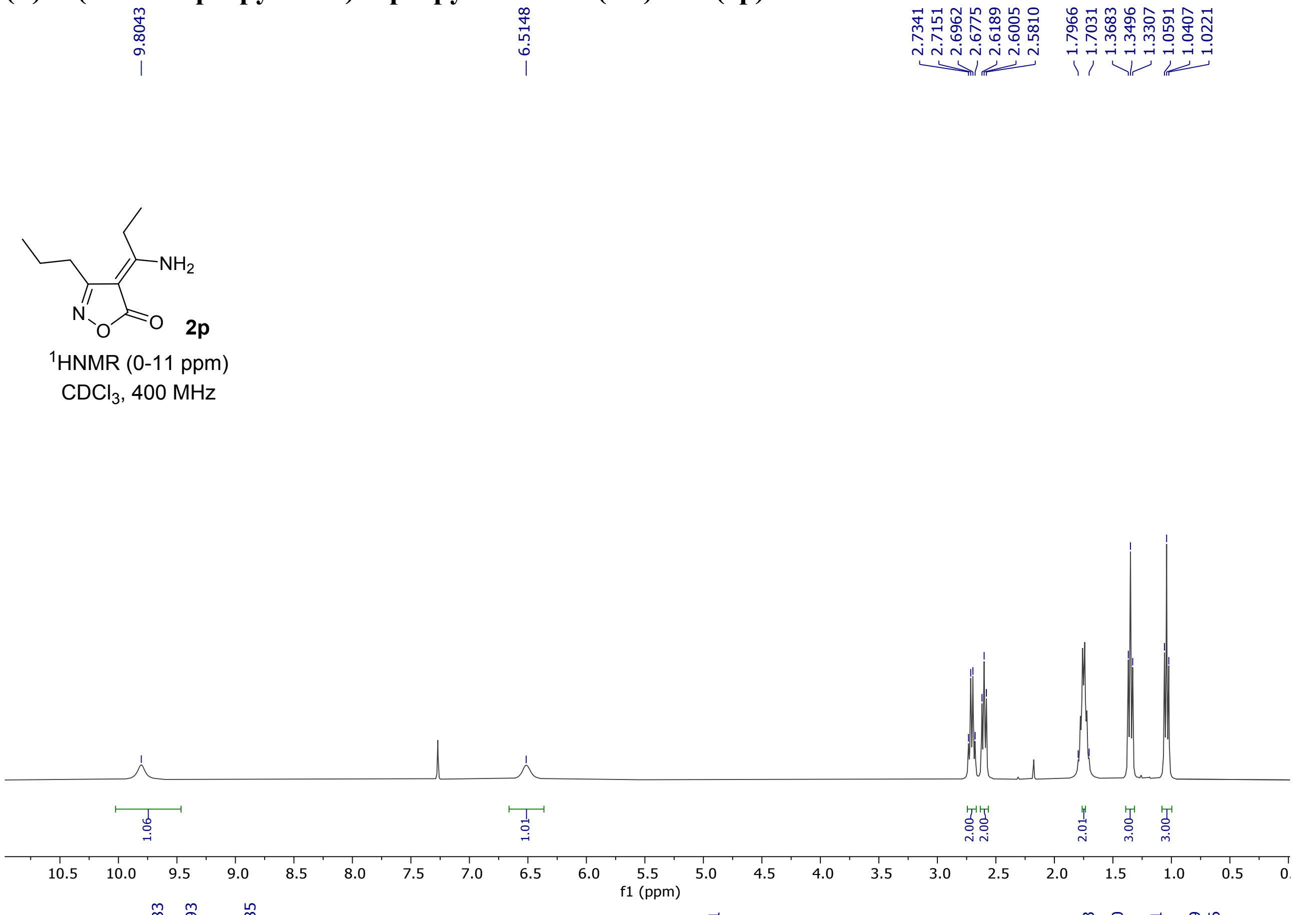

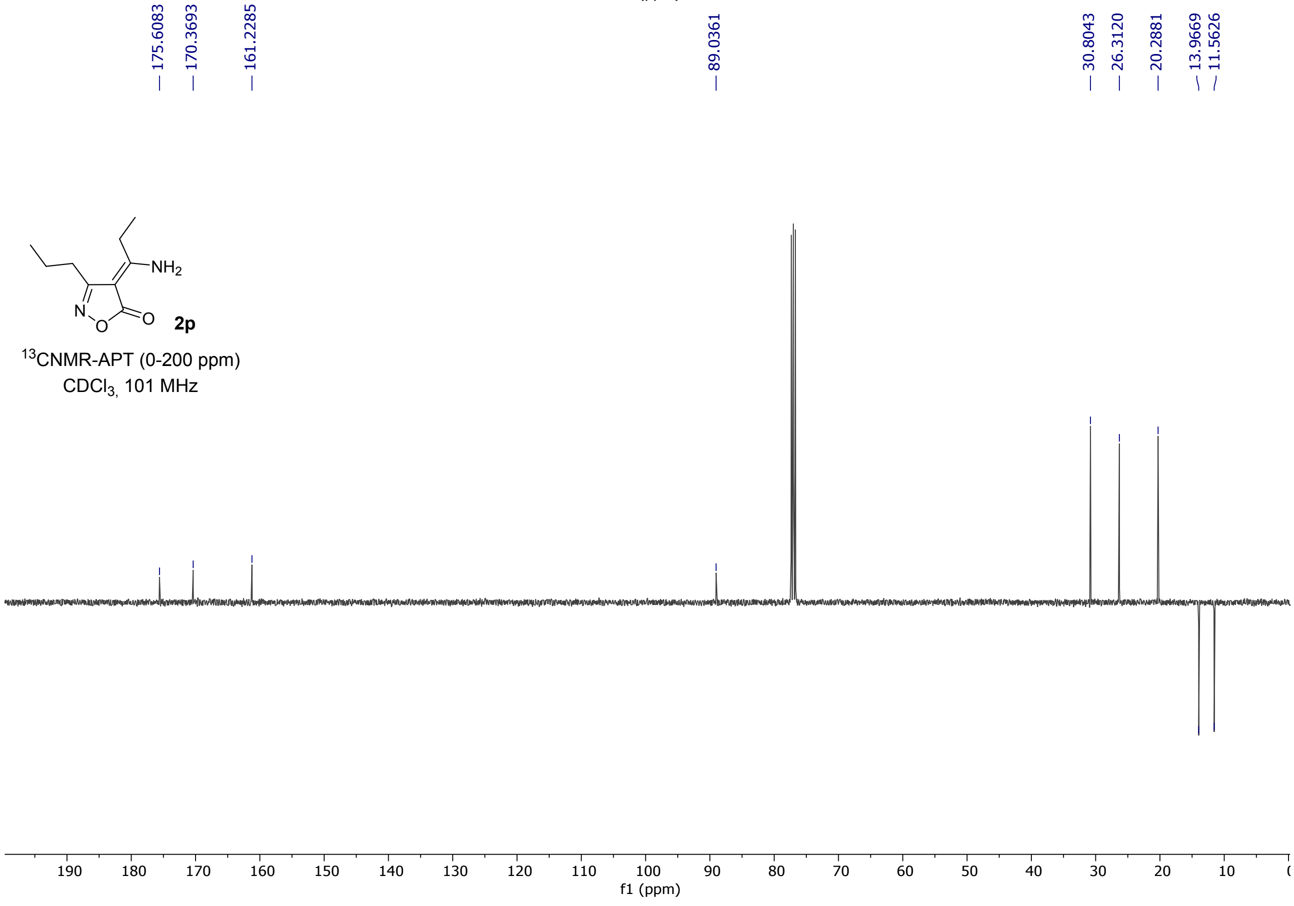

(*Z*)-4-(1-Aminoethylidene)-3-phenylisoxazol-5(4*H*)-one (2q)

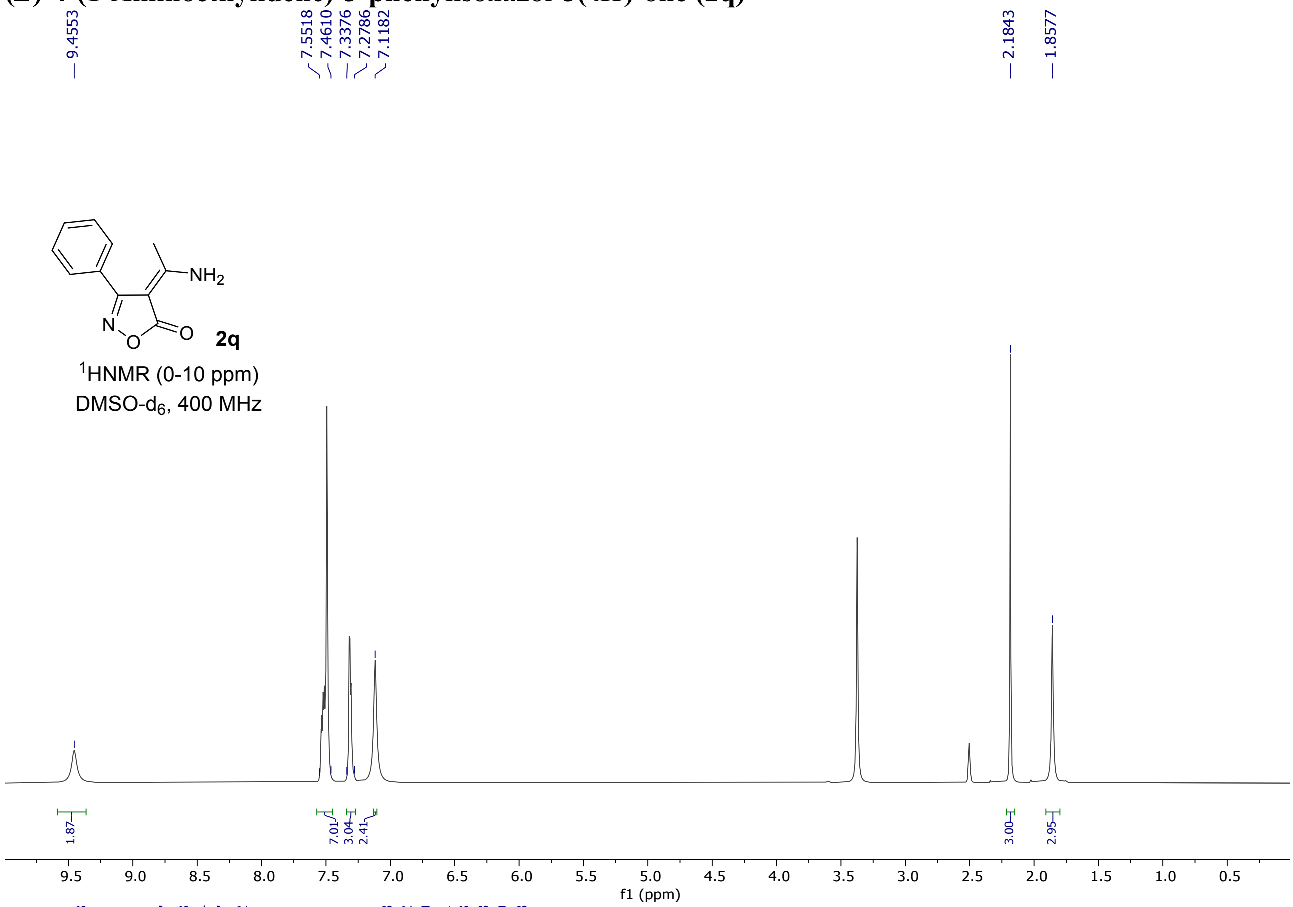

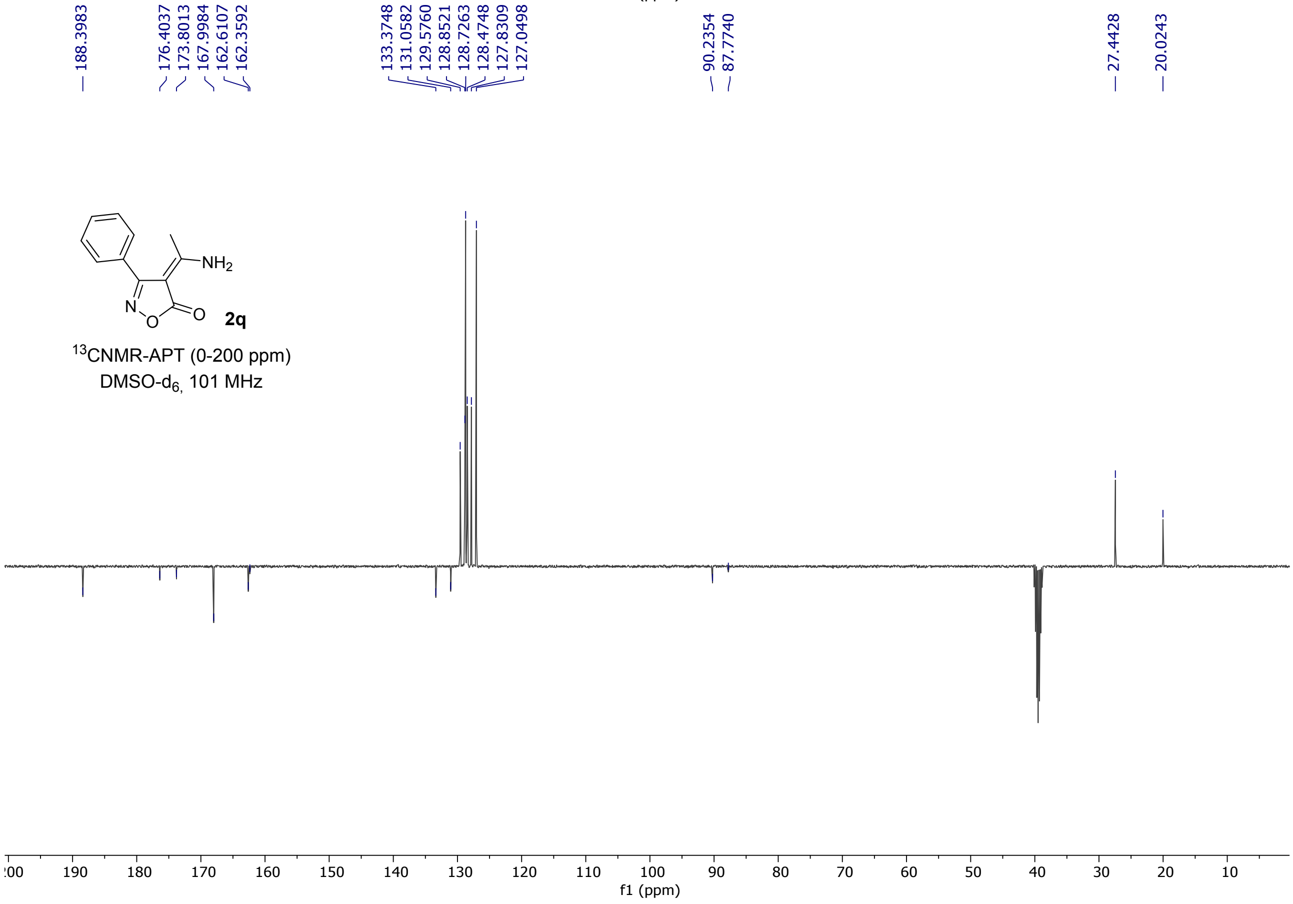

(*Z*)-4-(1-Hydroxyethylidene)-3-phenylisoxazol-5(4*H*)-one (5a)

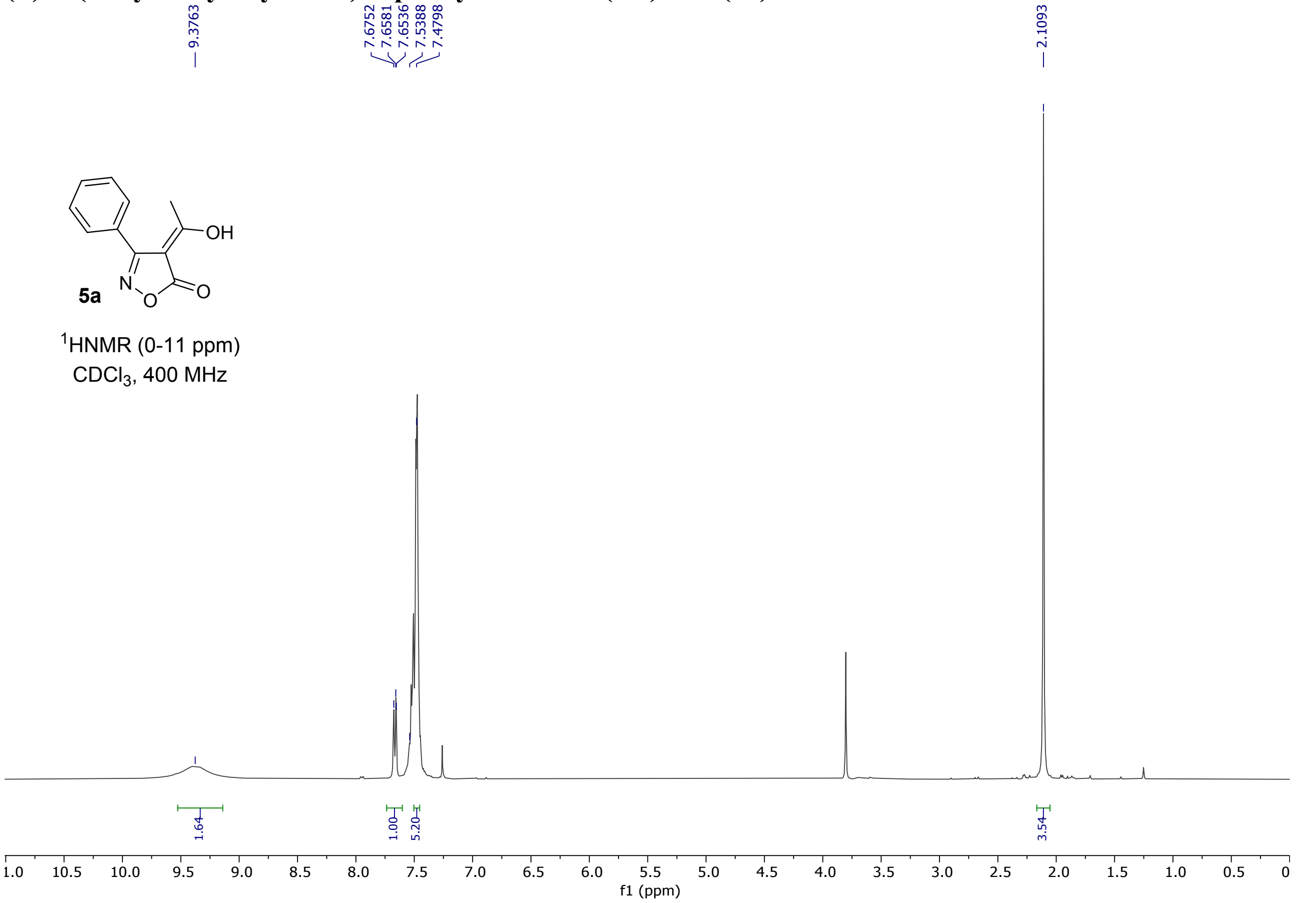

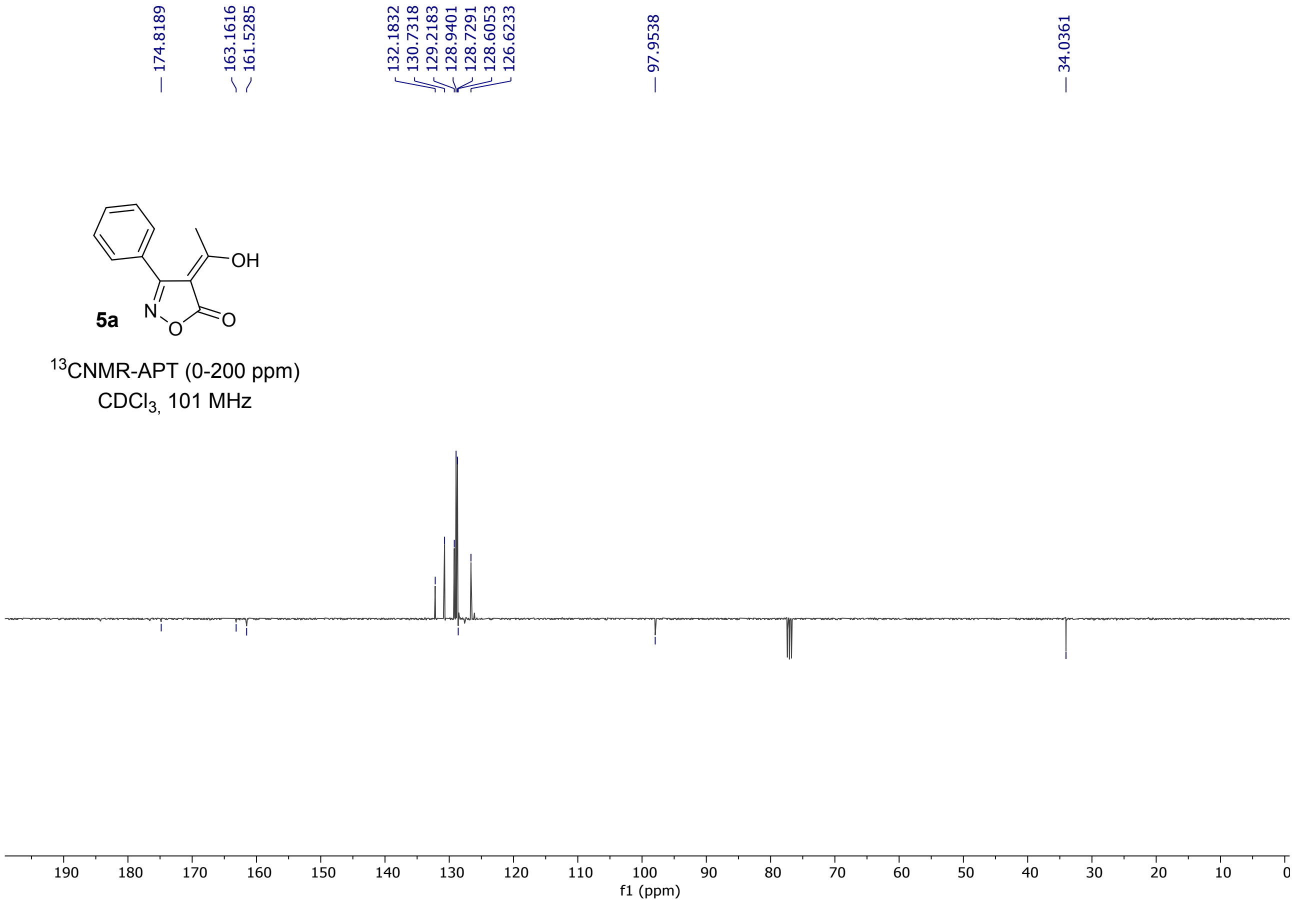

(*Z*)-4-(1-Hydroxyhexylidene)-3-phenylisoxazol-5(4*H*)-one (5c)

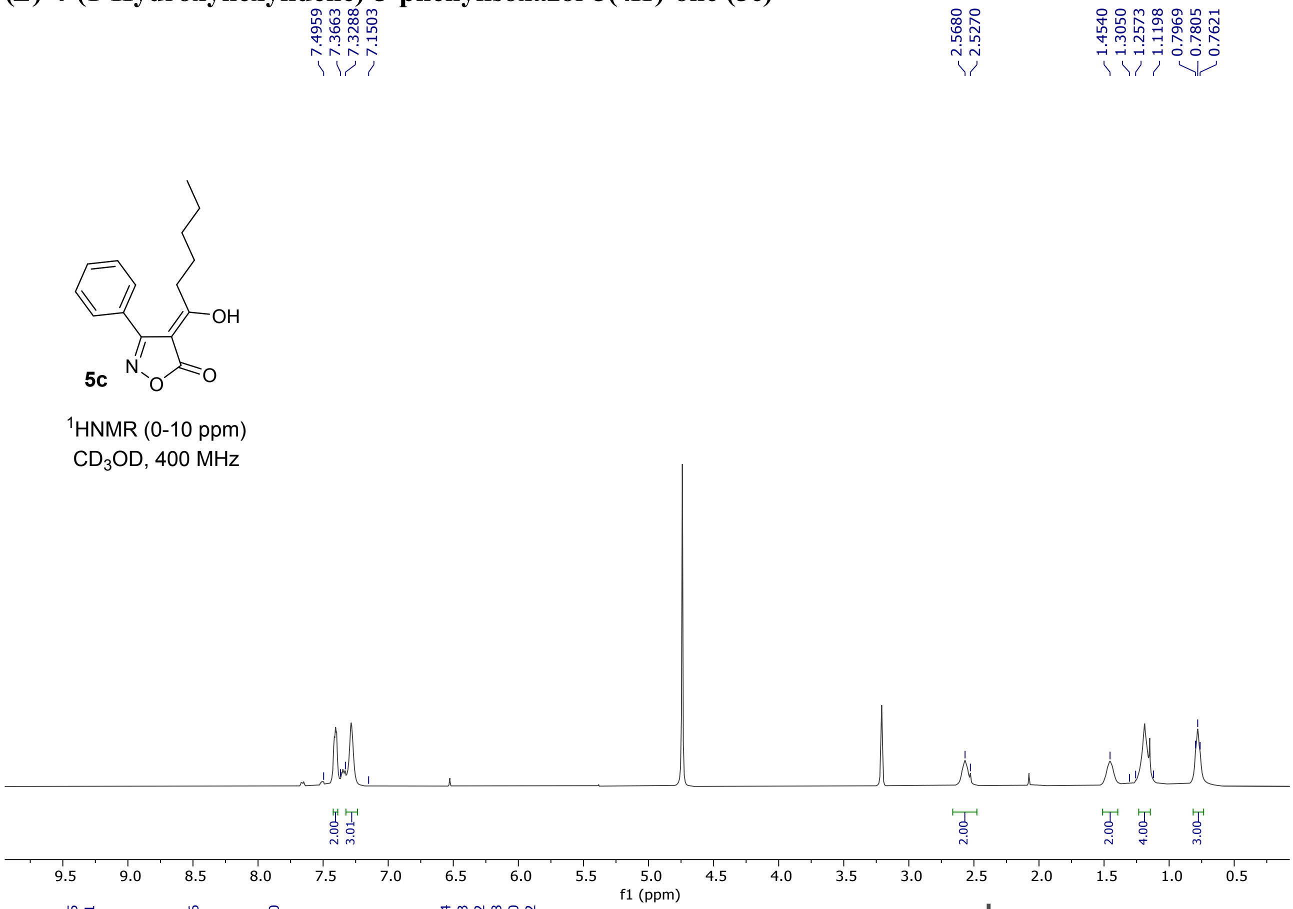

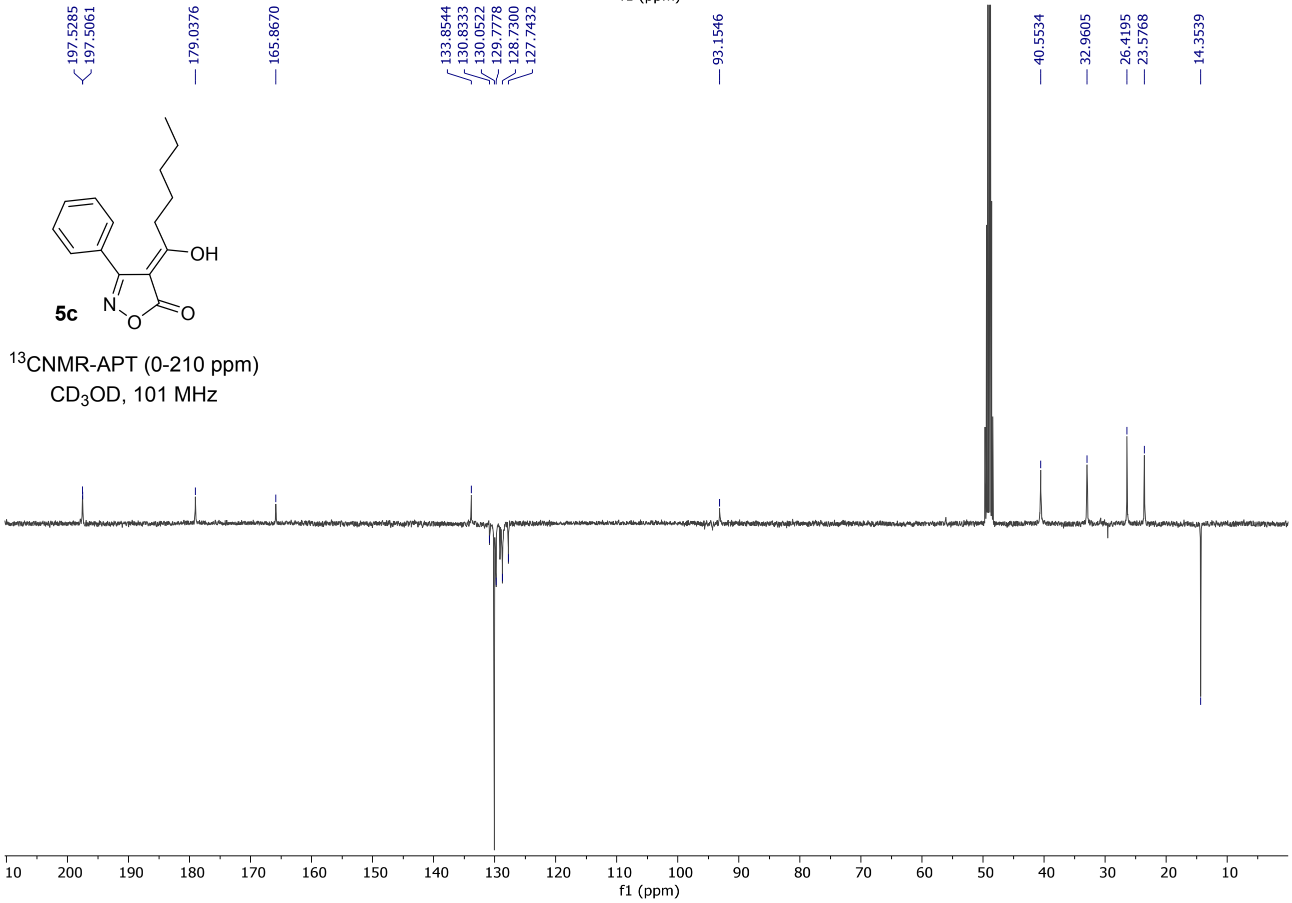

(*Z*)-4-(Hydroxy(phenyl)methylene)-3-phenylisoxazol-5(4*H*)-one (5d)

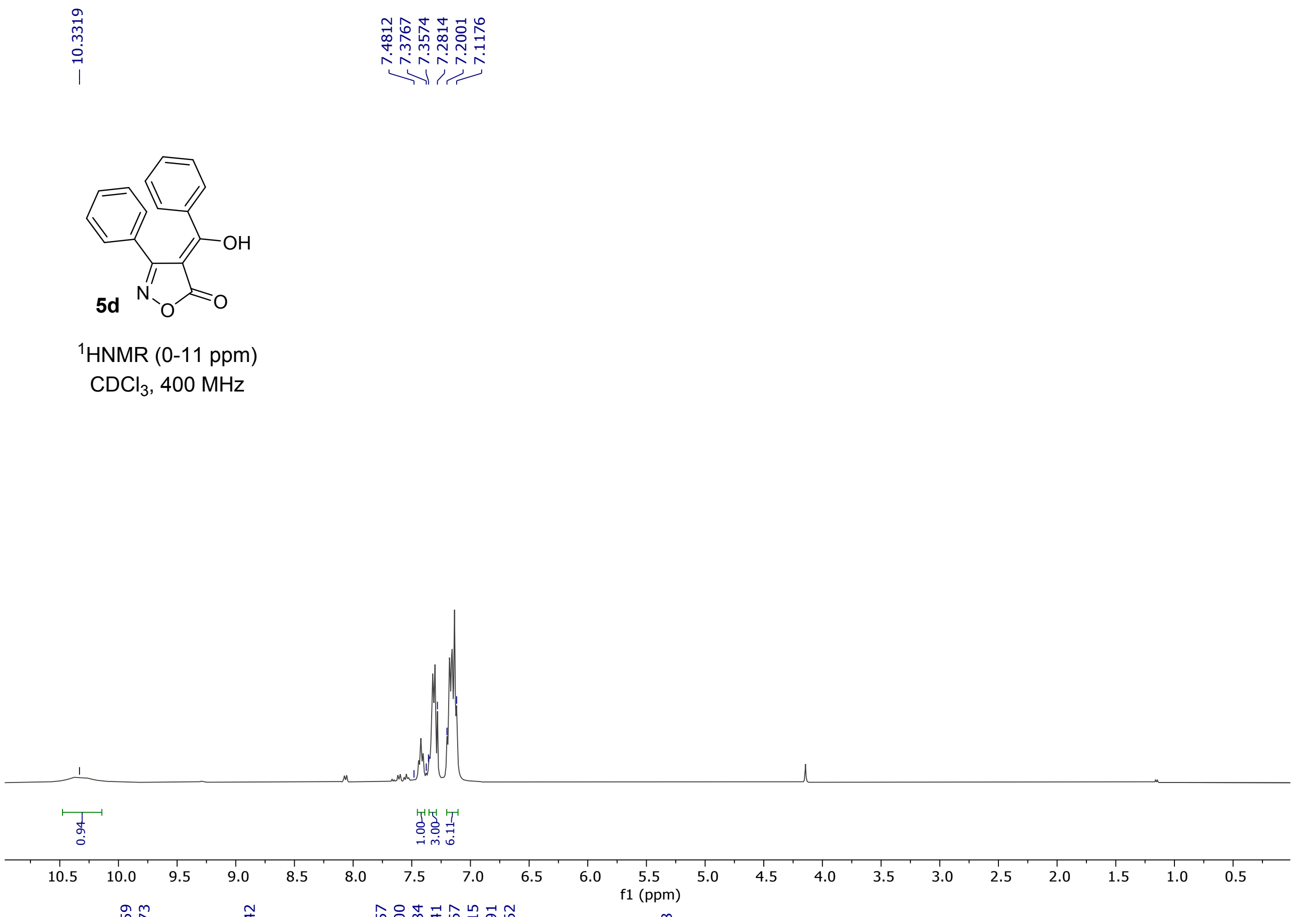

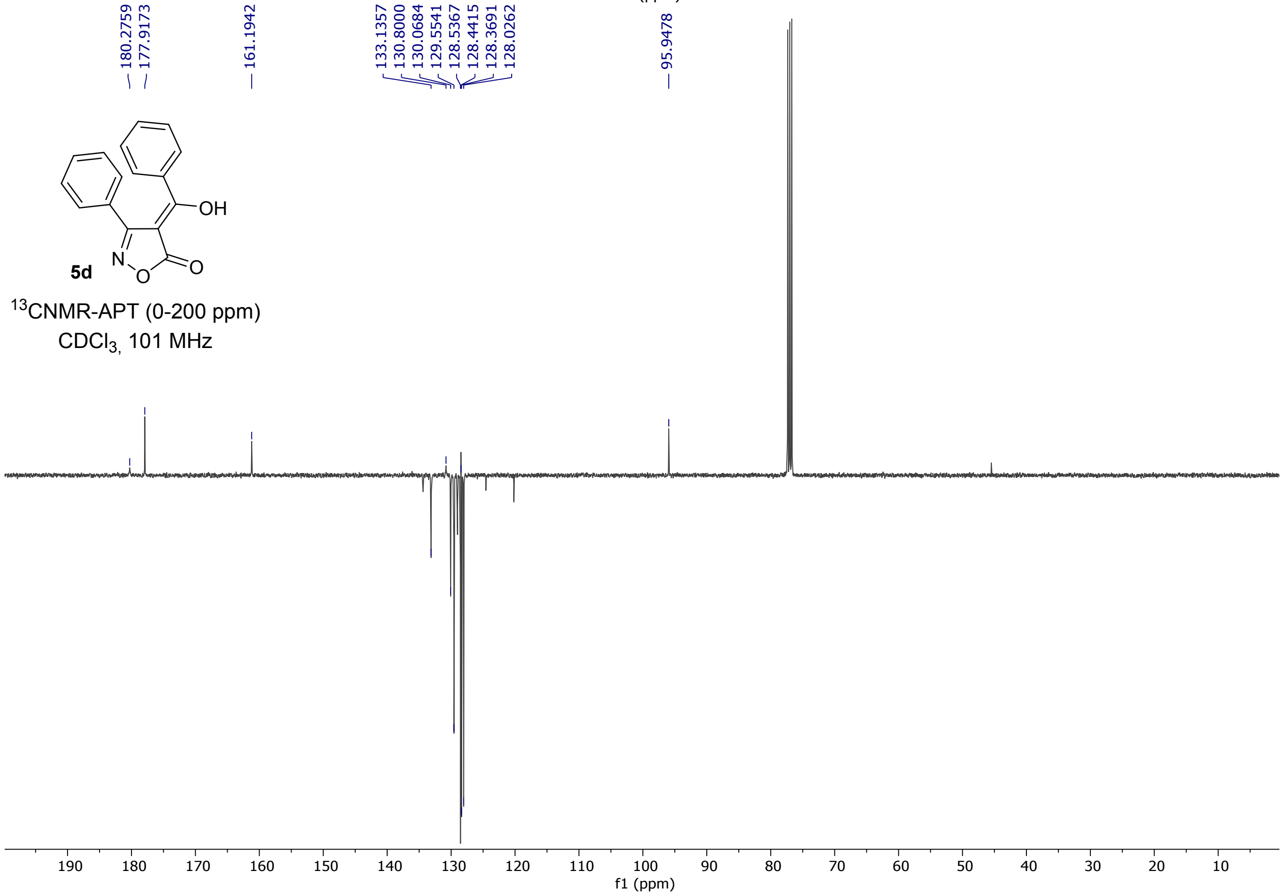

(*Z*)-4-(1-Hydroxypropylidene)-3-propylisoxazol-5(4*H*)-one (5e)

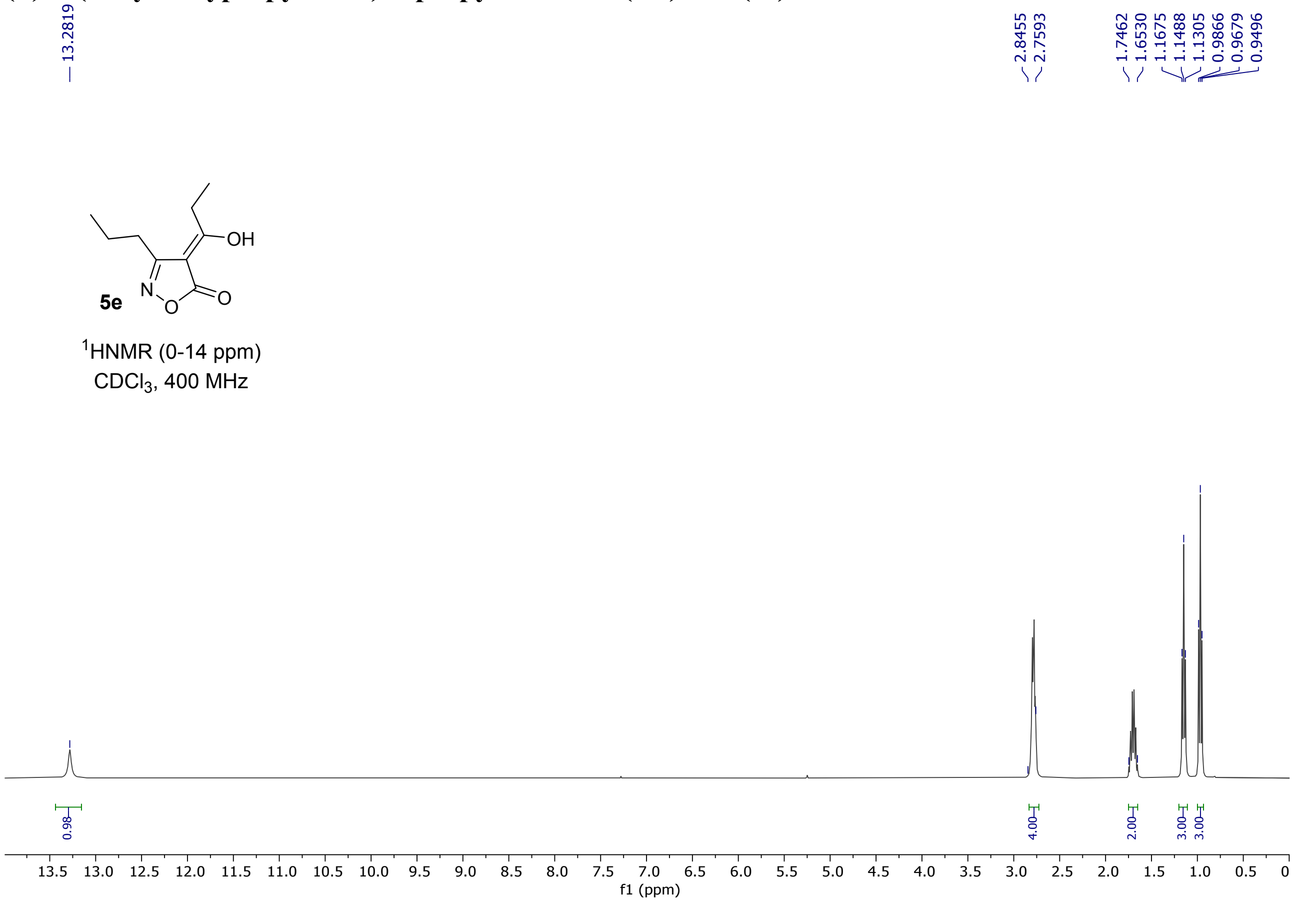

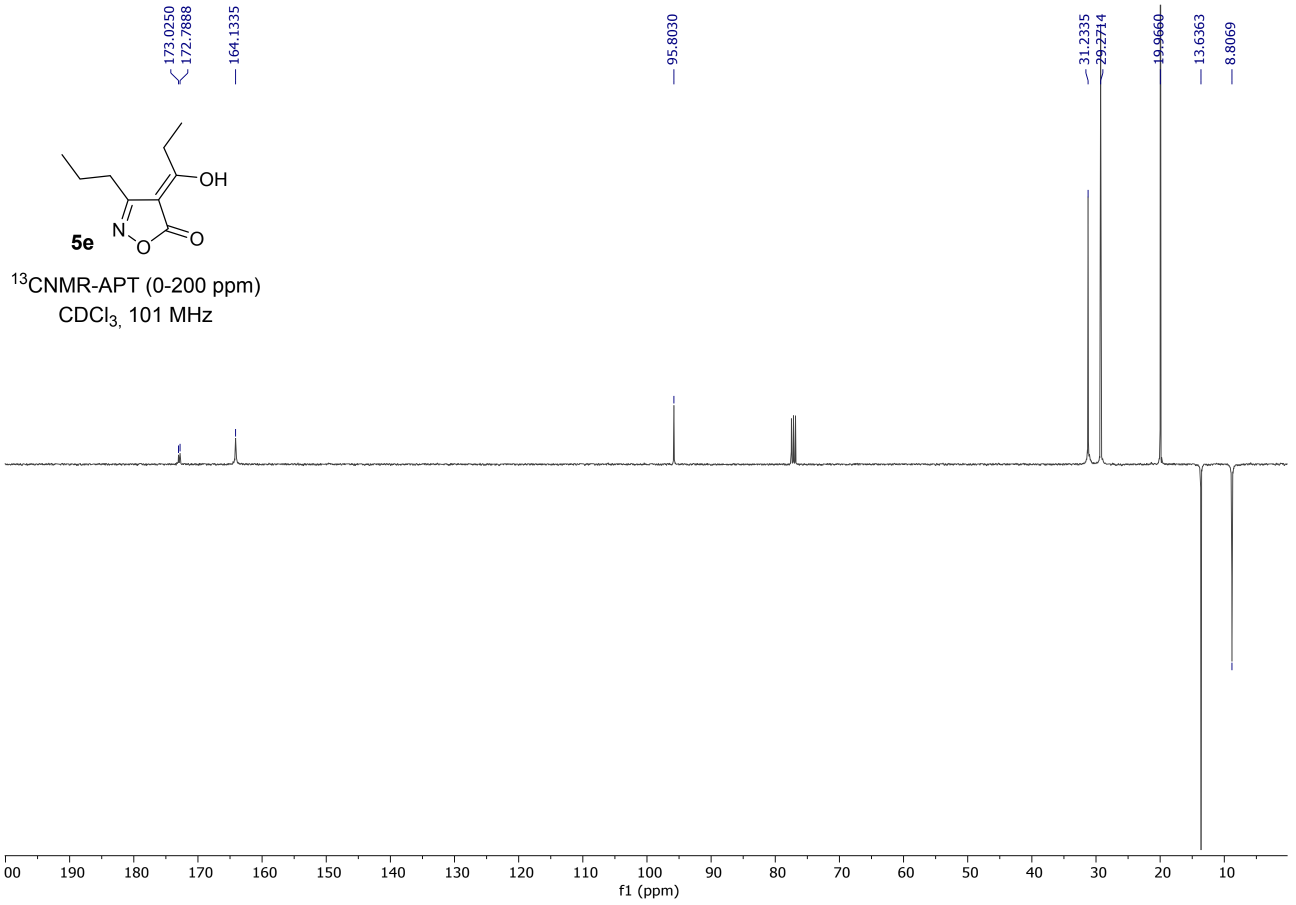

(*Z*)-4-(Hydroxy(phenyl)methylene)-3-methylisoxazol-5(4*H*)-one (5f)

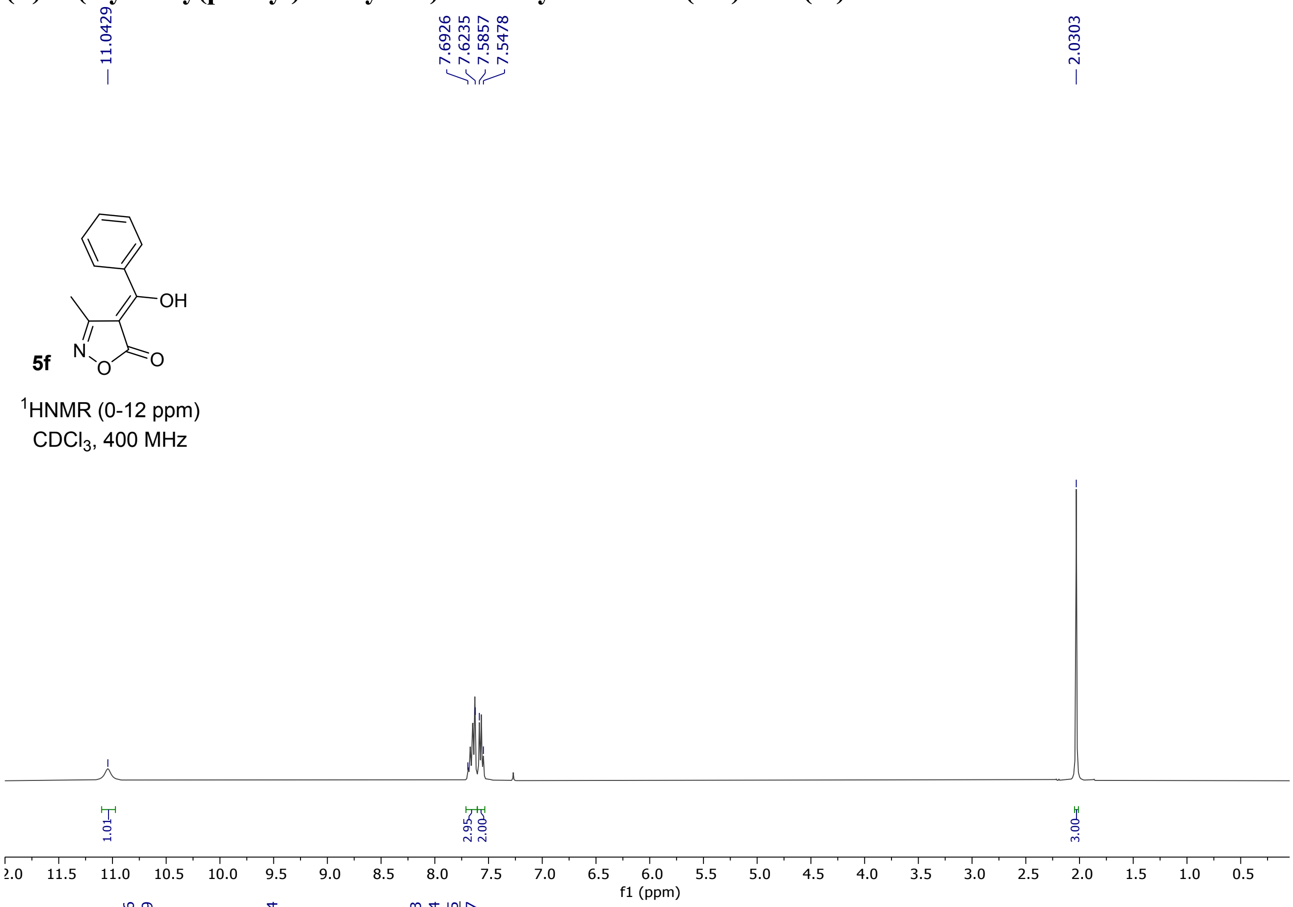

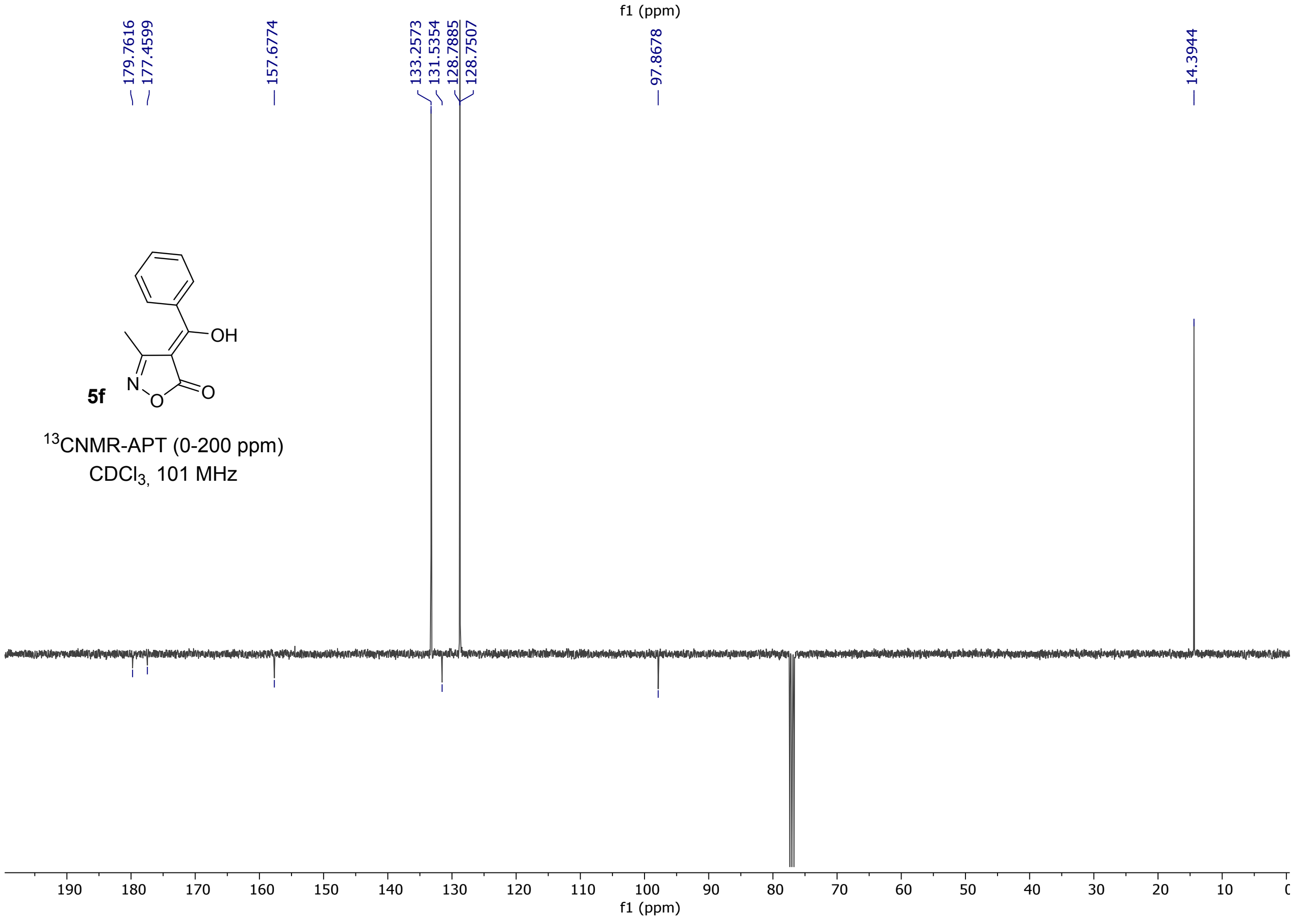

1-Benzyl-3-phenyl-1*H*-pyrazole-4-carboxylic acid (3a)

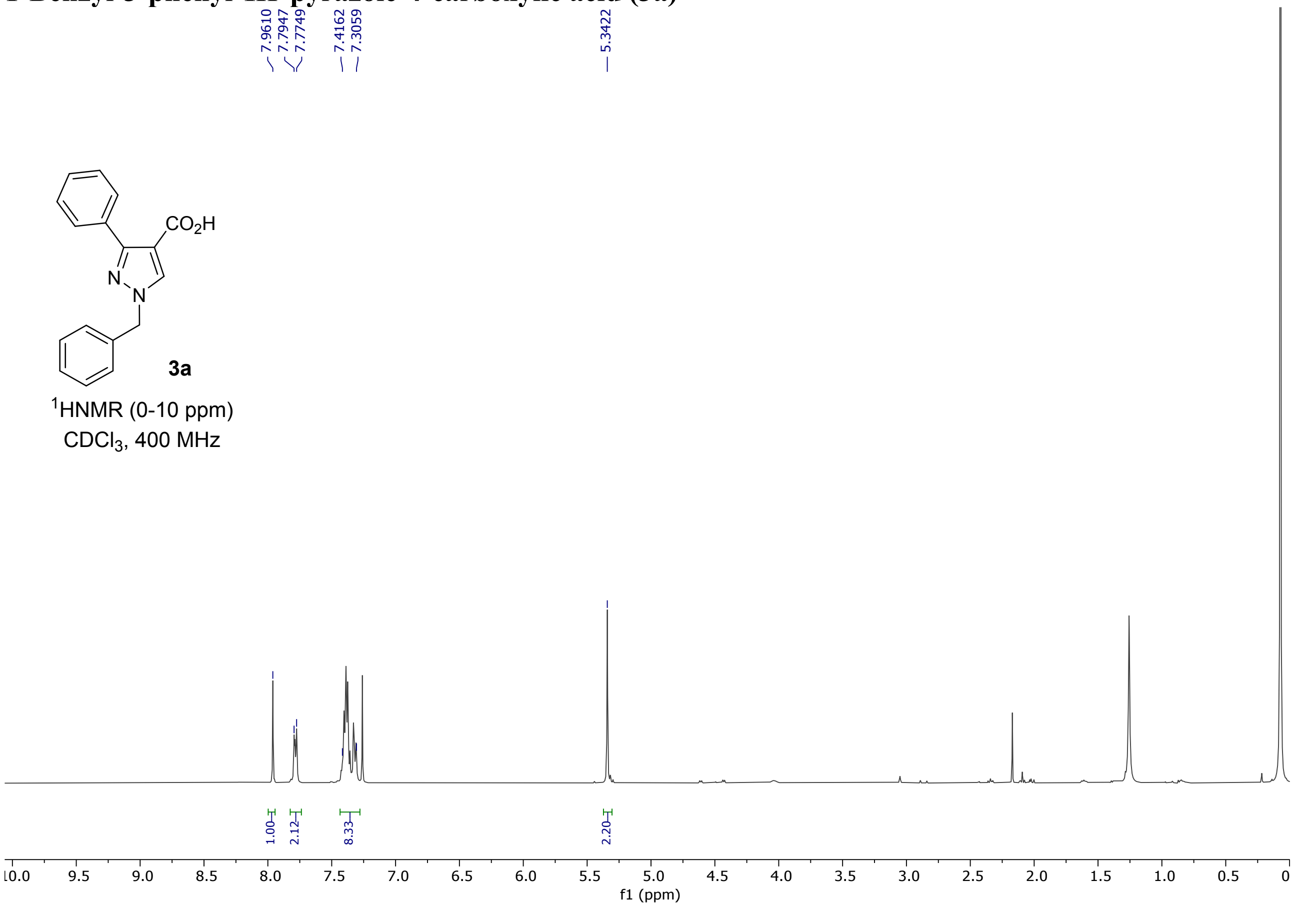

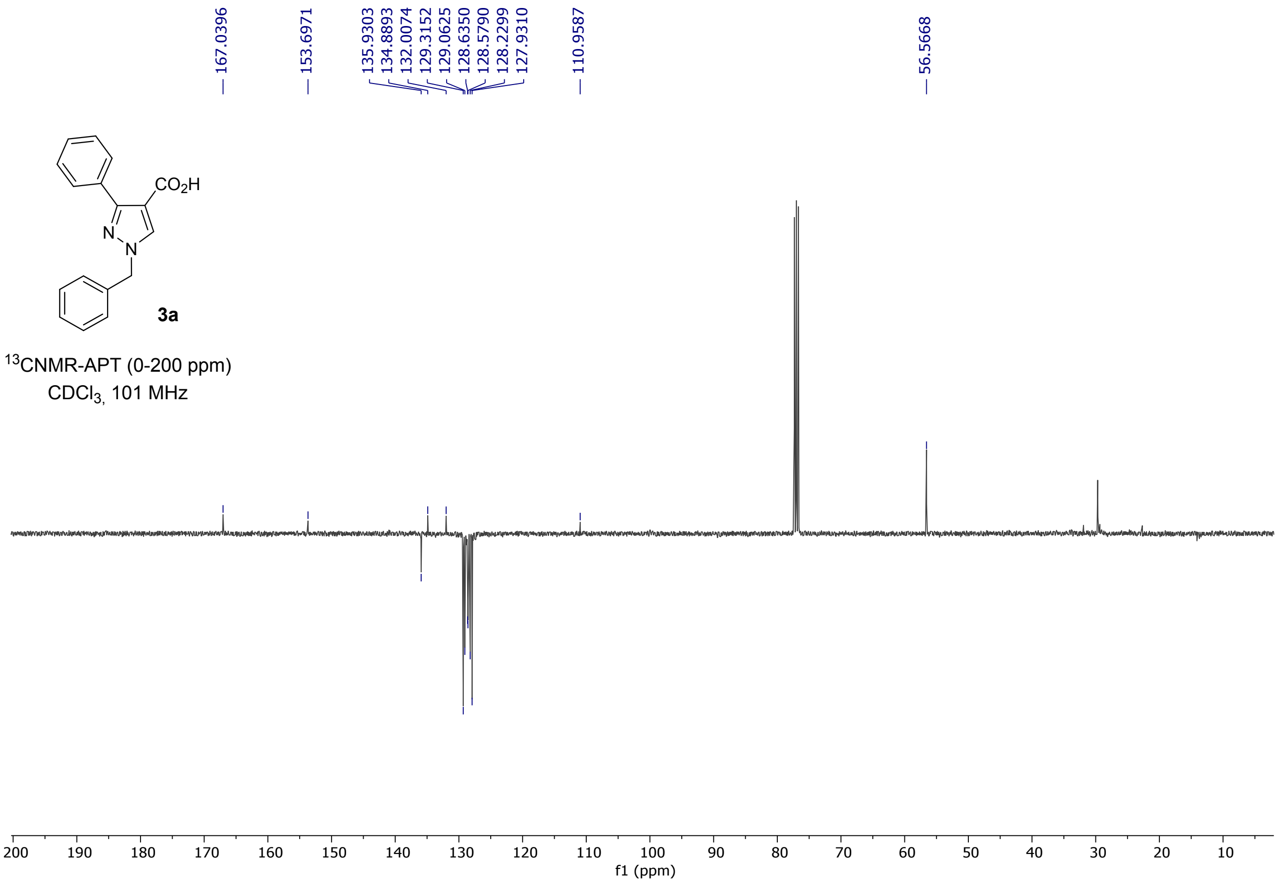

1,3-Diphenyl-1*H*-pyrazole-4-carboxylic acid (3b)

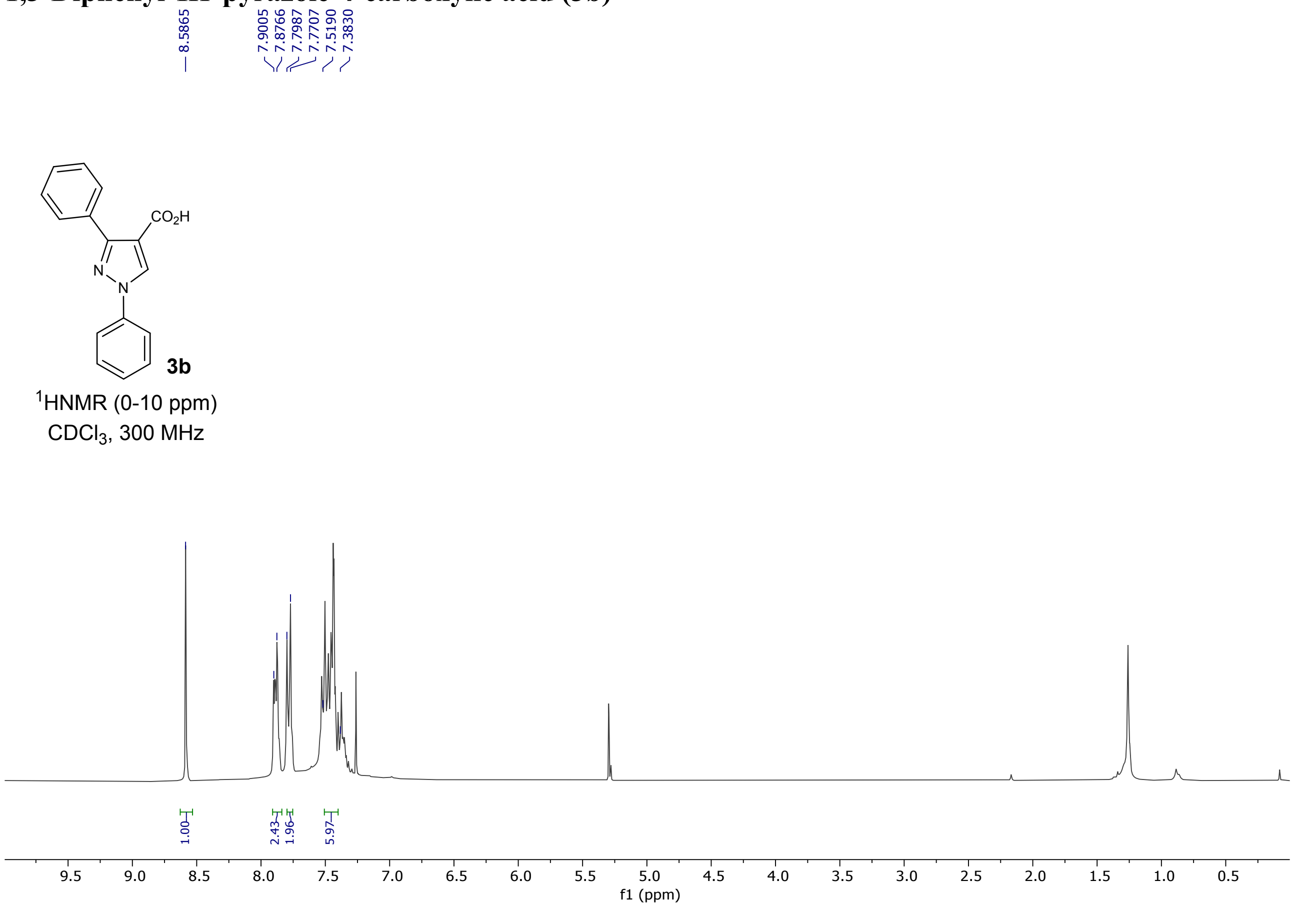

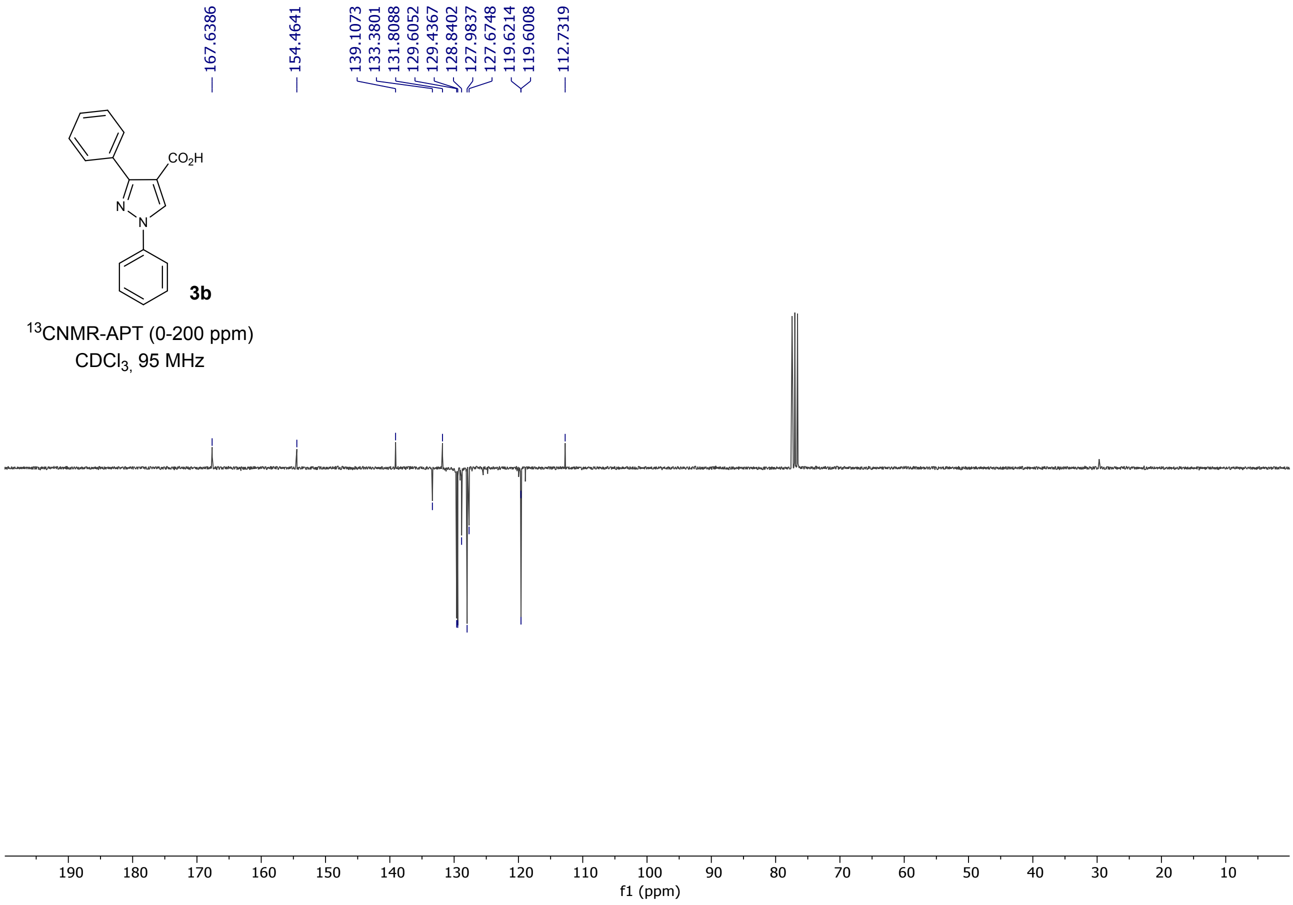

3-Phenyl-1-(2-tolyl)-1*H*-pyrazole-4-carboxylic acid (3c)

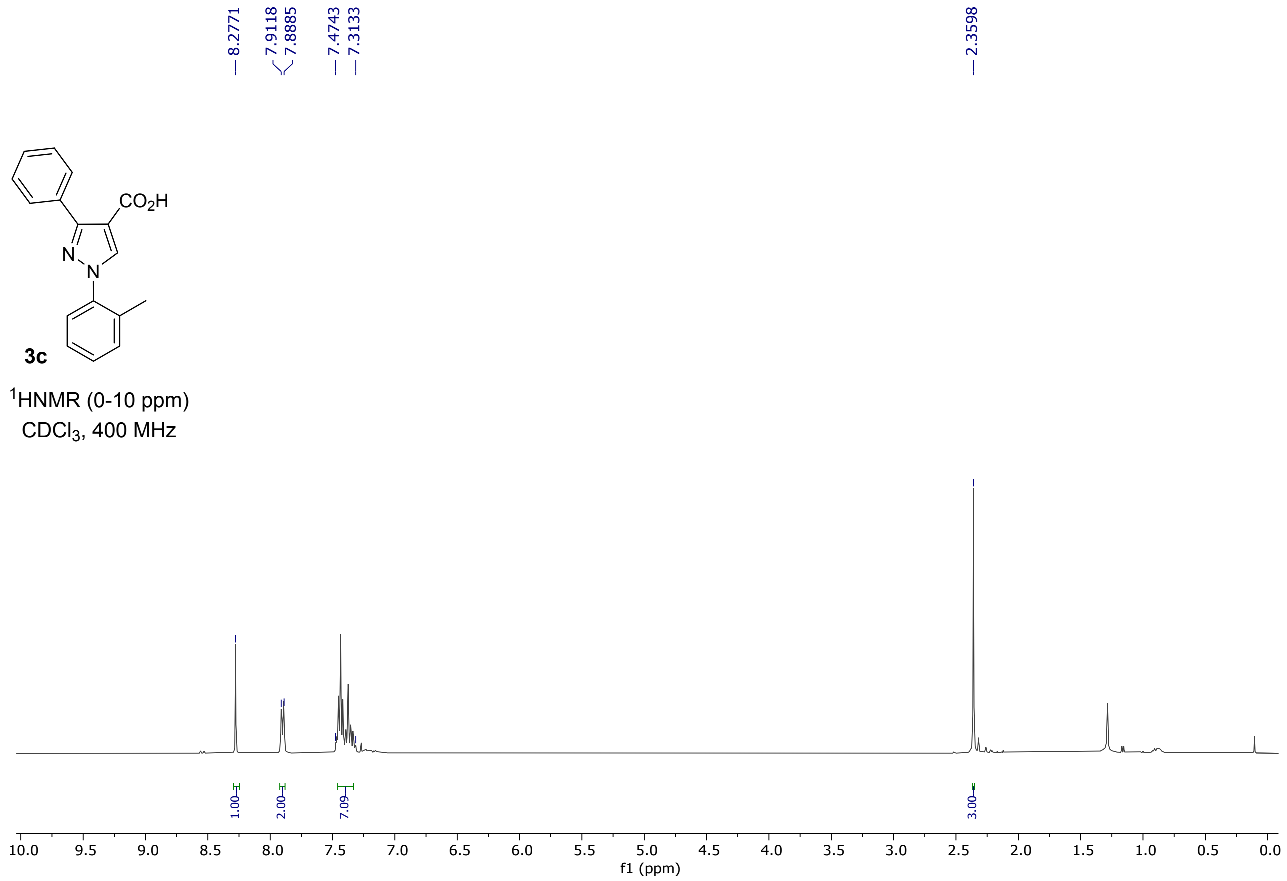

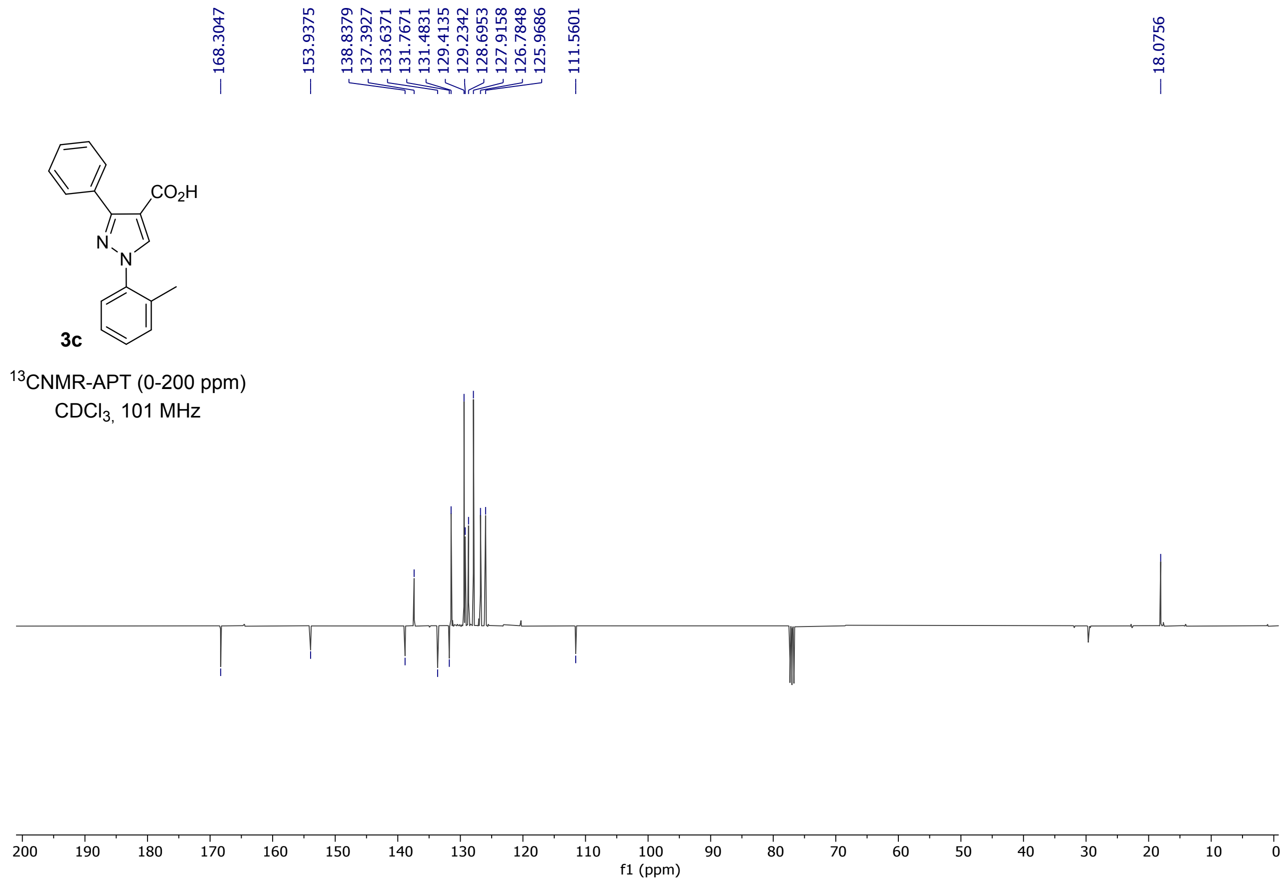

1-(2-Iodophenyl)-3-phenyl-1*H*-pyrazole-4-carboxylic acid (3d)

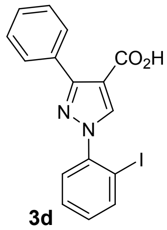

3d

^{1}HNMR (0-10 ppm)
$CDCl_3$, 400 MHz

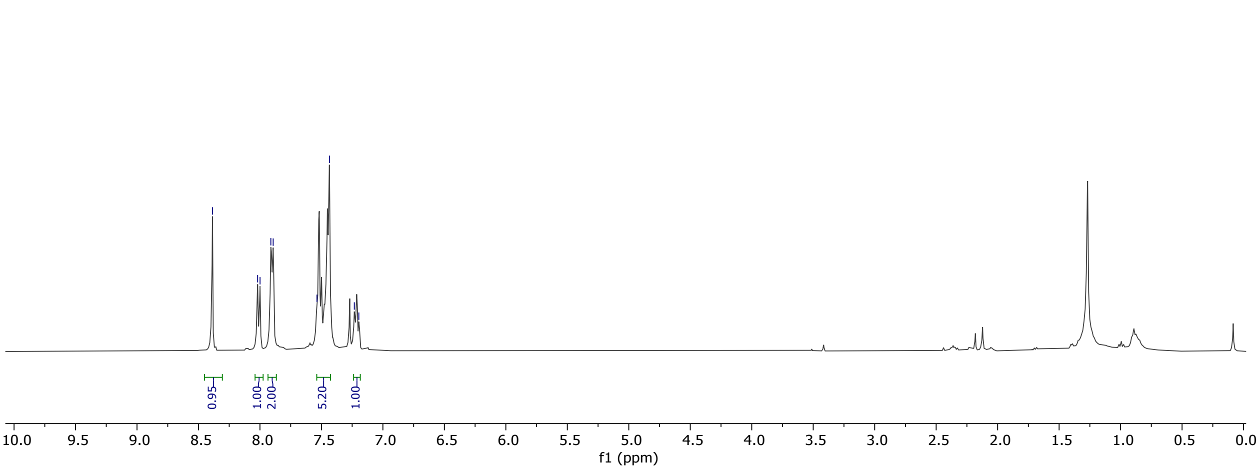

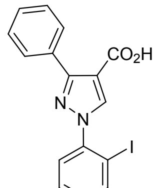

3d

^{13}CNMR-APT (0-200 ppm)
$CDCl_3$, 101 MHz

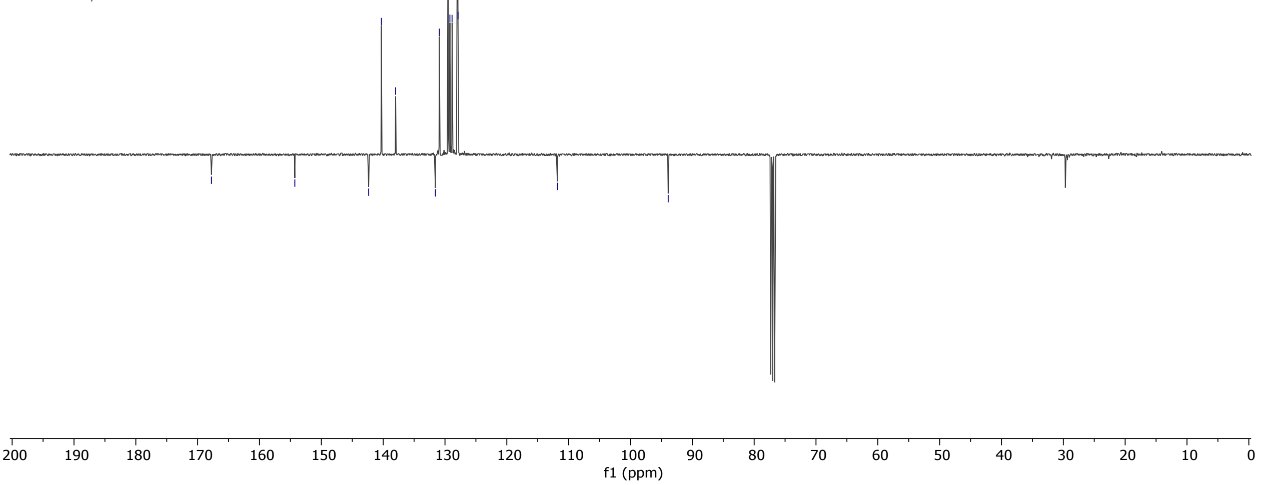

1-(4-Methoxyphenyl)-3-phenyl-1*H*-pyrazole-4-carboxylic acid (3e)

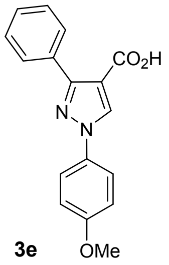

3e

^{1}HNMR (0-10 ppm)
$CDCl_3$, 400 MHz

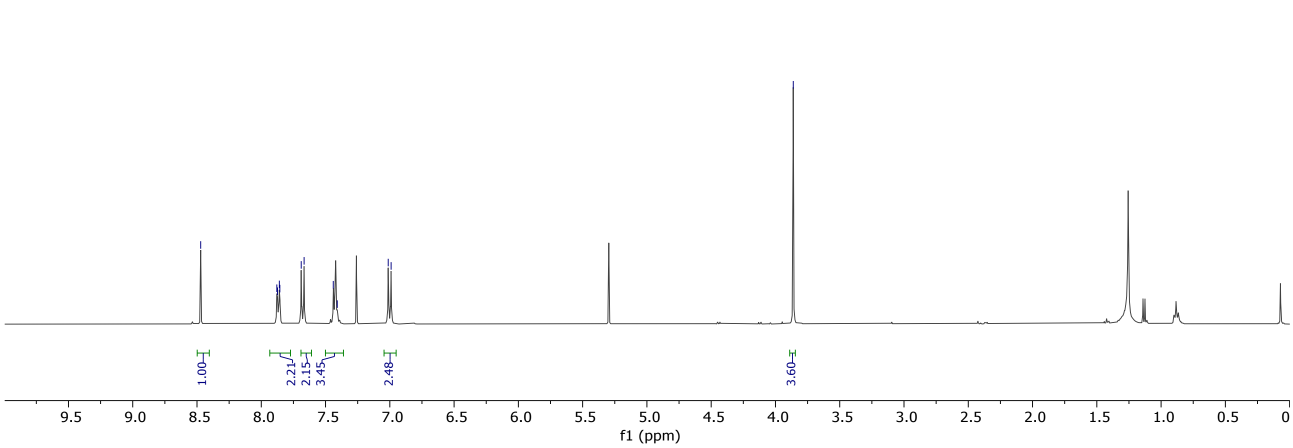

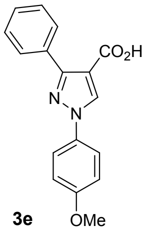

3e

^{13}CNMR-APT (0-200 ppm)
$CDCl_3$, 101 MHz

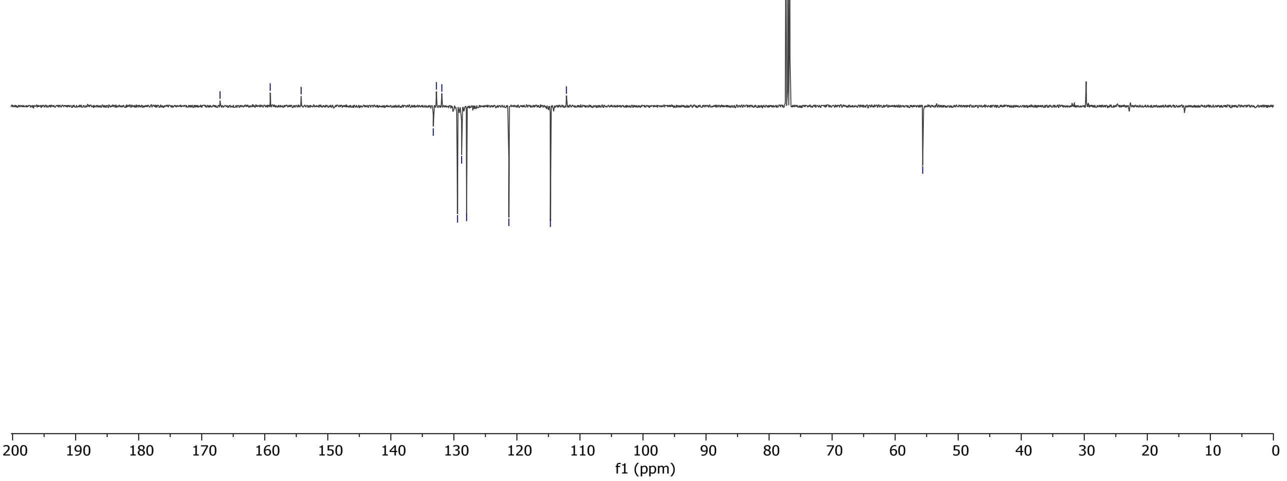

1-Phenyl-3-propyl-1*H*-pyrazole-4-carboxylic acid (3f)

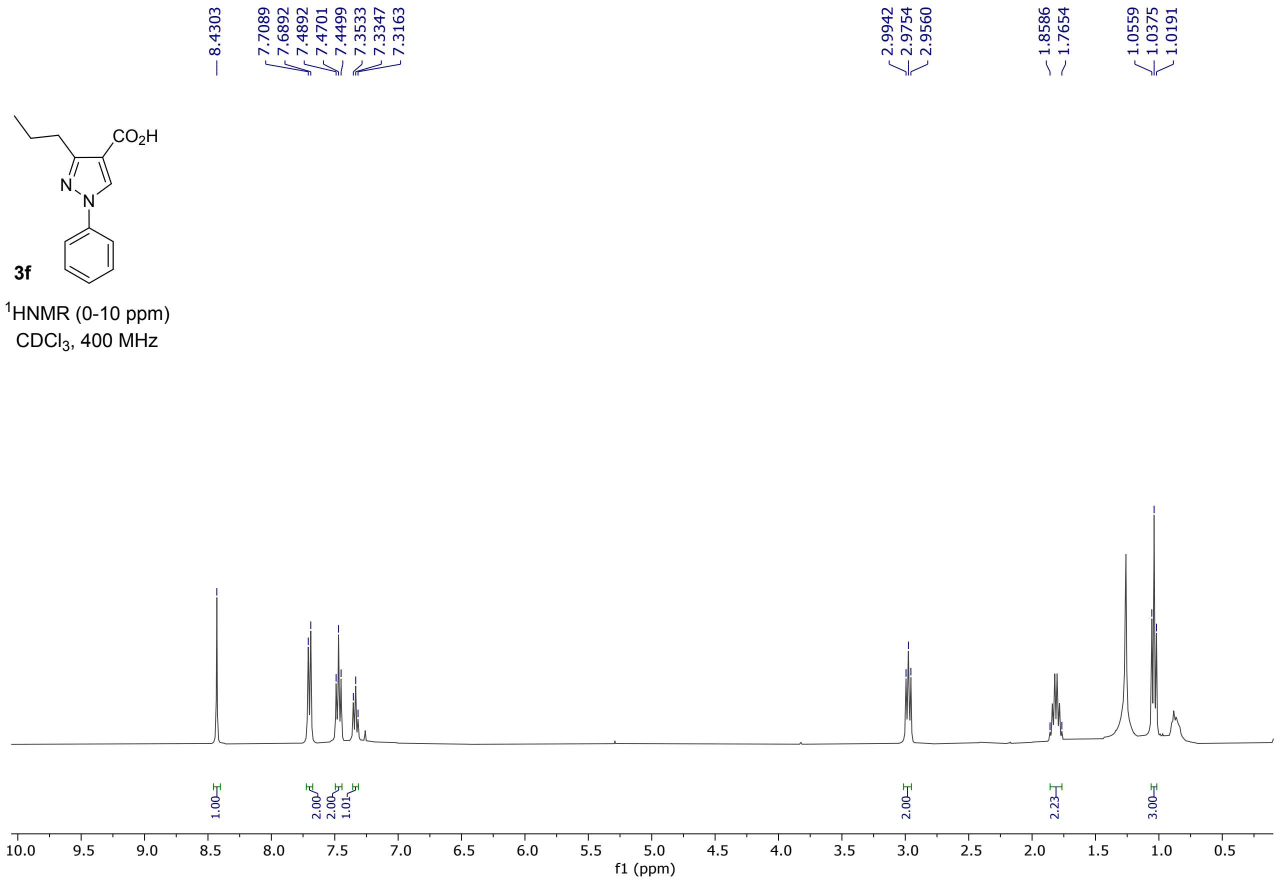

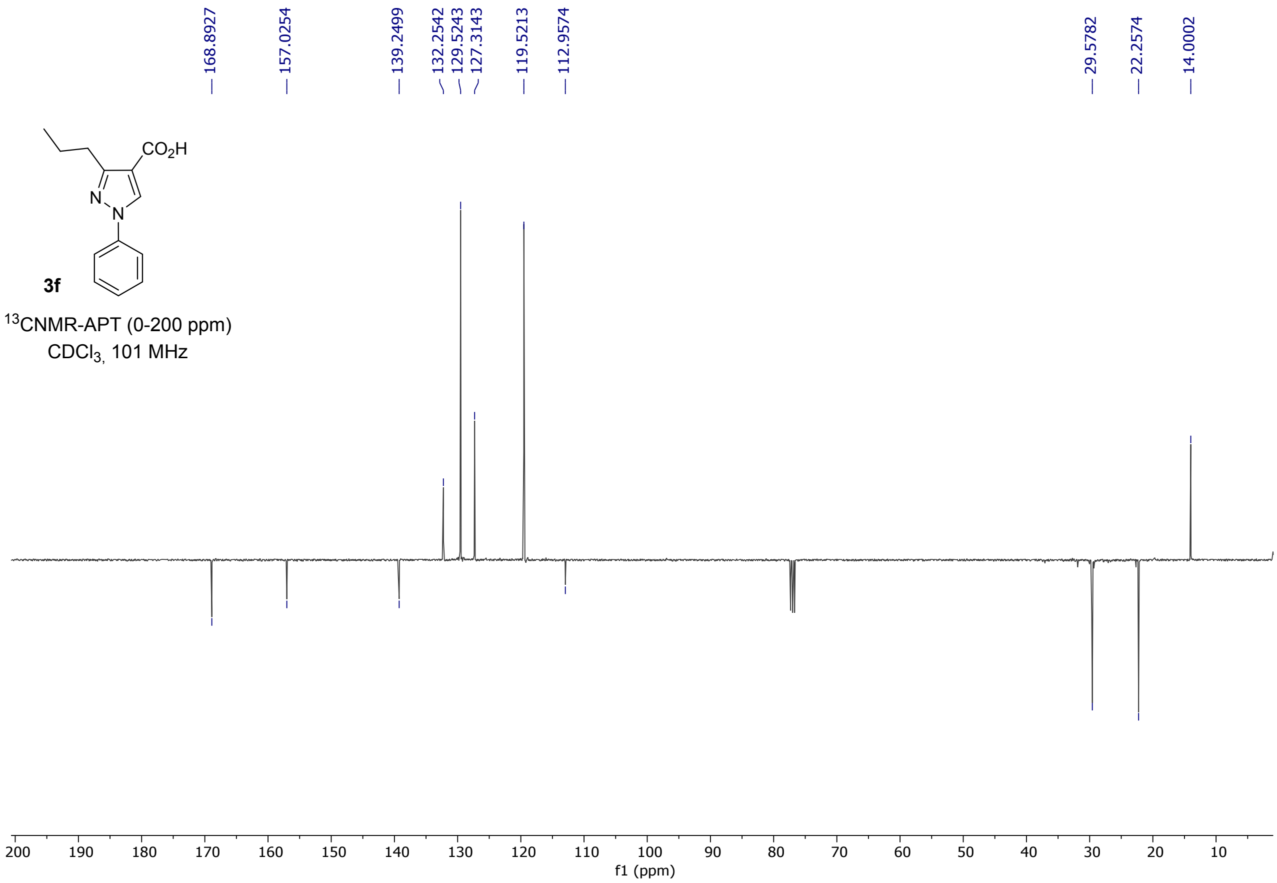

3-Propyl-1-(4-tolyl)-1*H*-pyrazole-4-carboxylic acid (3g)

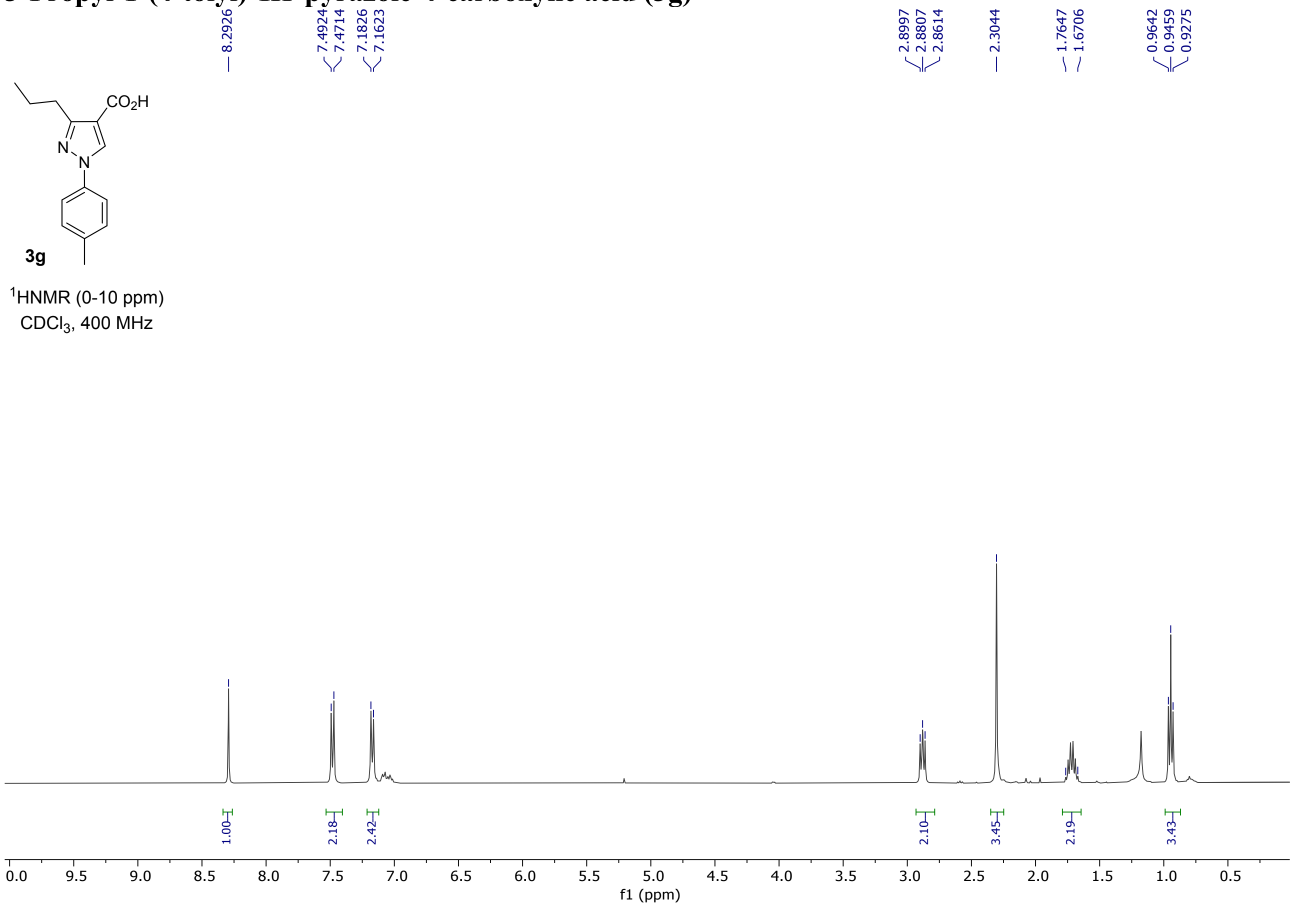

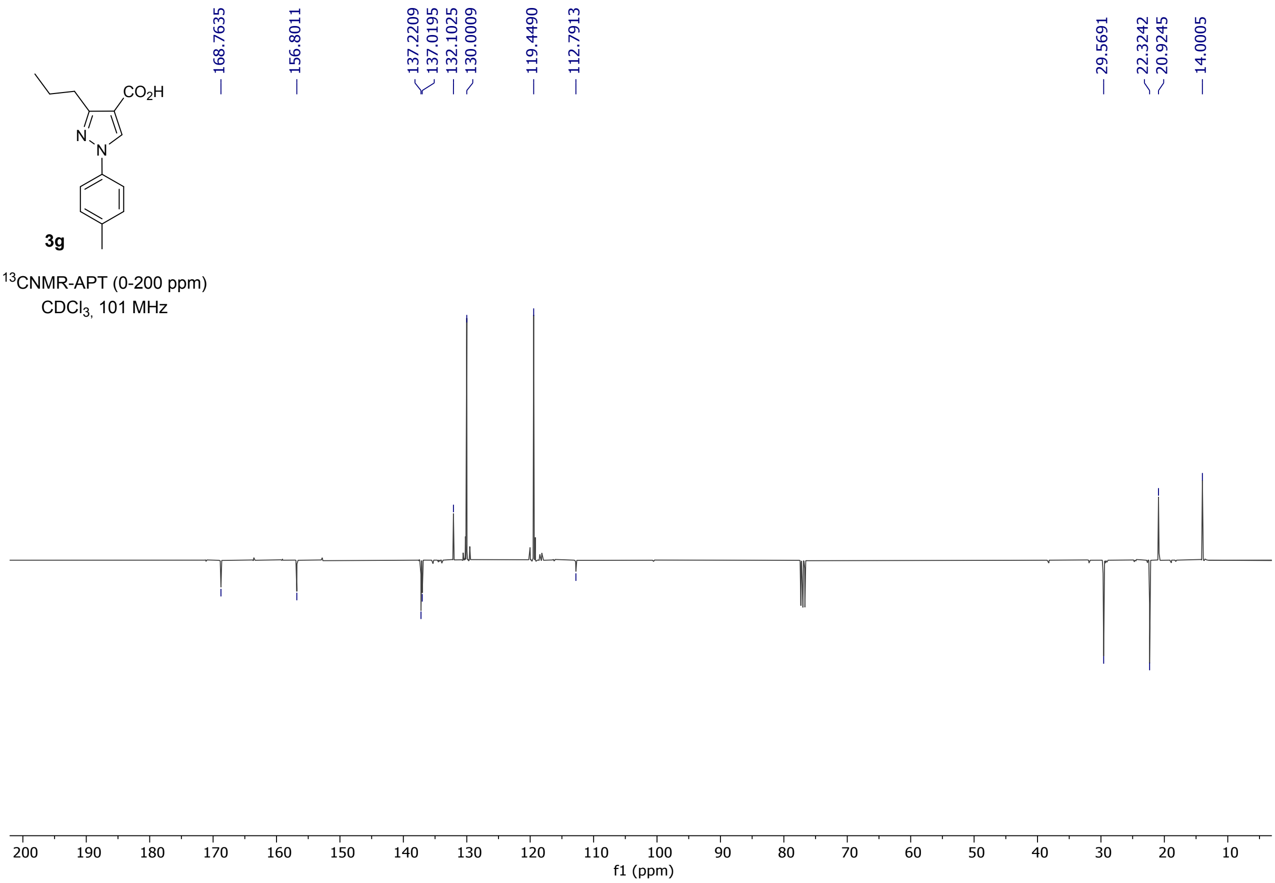

1-(4-Methoxyphenyl)-3-propyl-1*H*-pyrazole-4-carboxylic acid (3h)

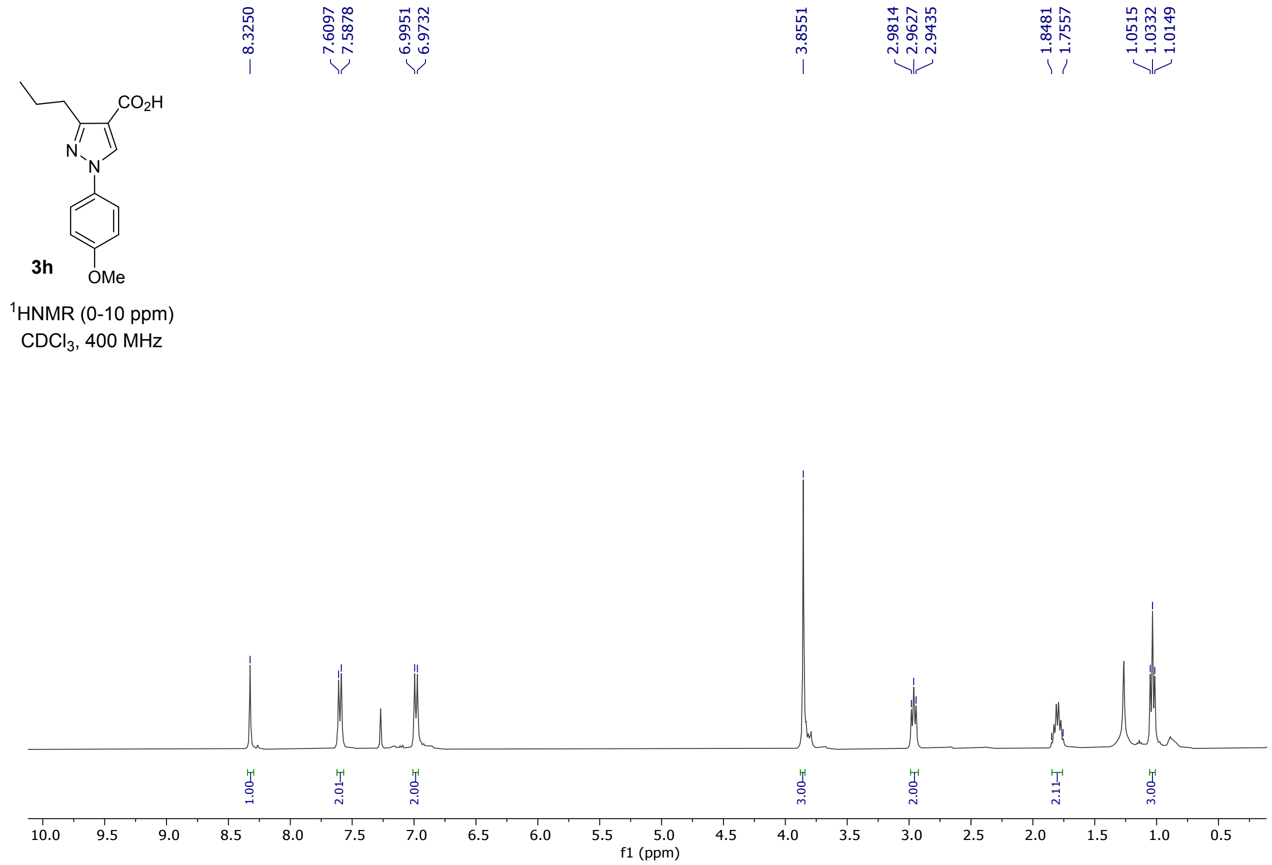

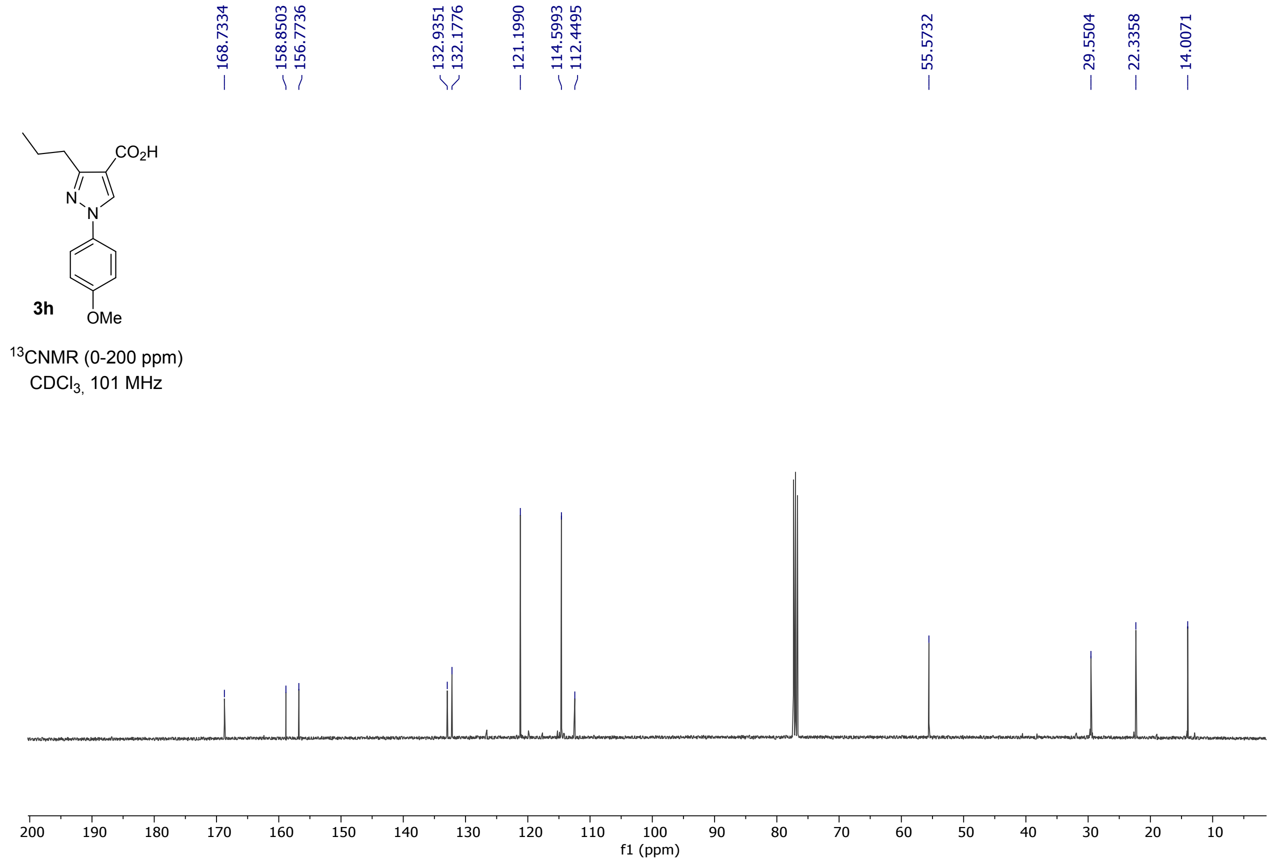

3-Propyl-1-(3-tolyl)-1*H*-pyrazole-4-carboxylic acid (3i)

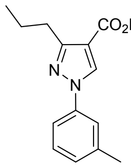

3i

^{1}HNMR (0-10 ppm)
$CDCl_3$, 400 MHz

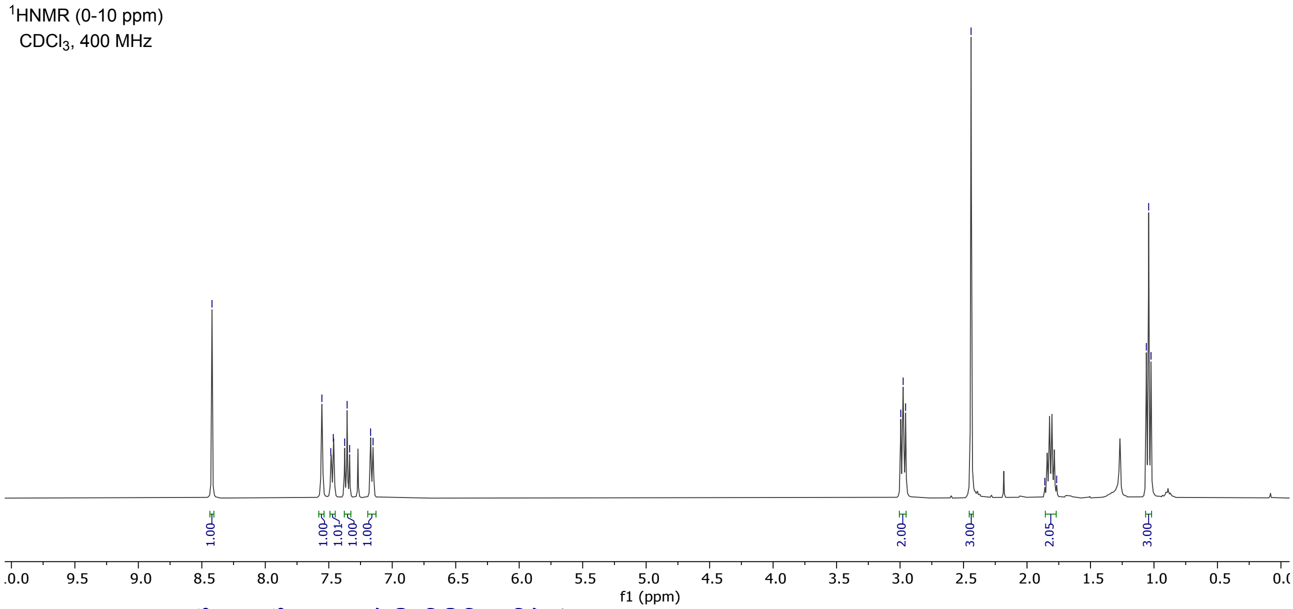

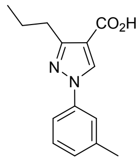

3i

^{13}CNMR-APT (0-200 ppm)
$CDCl_3$, 101 MHz

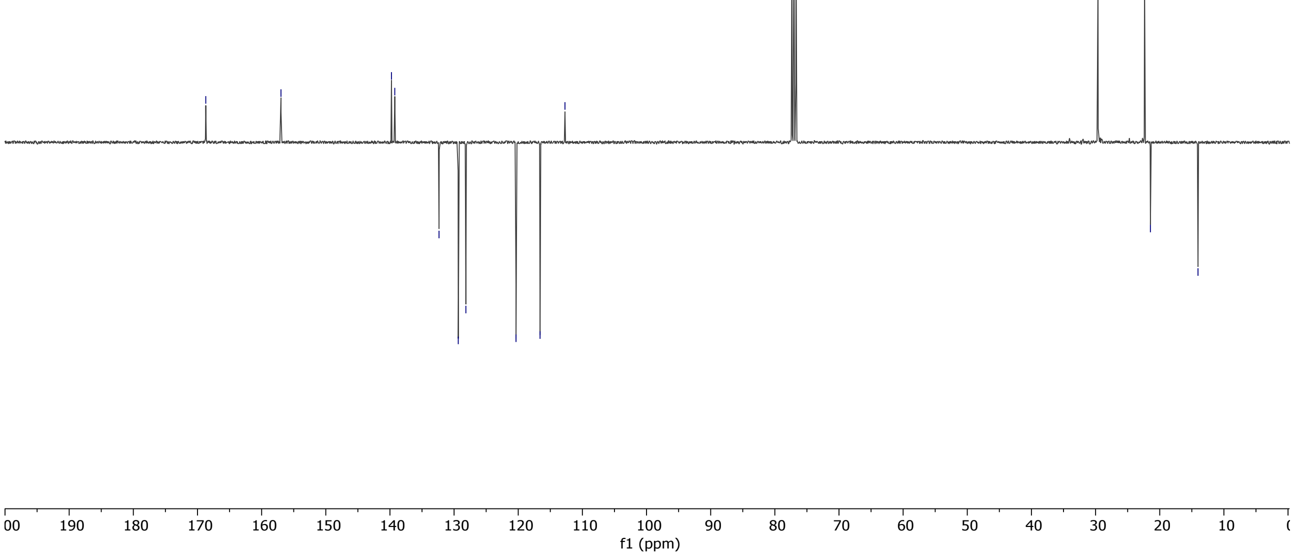

1-Phenethyl-3-propyl-1*H*-pyrazole-4-carboxylic acid (3j)

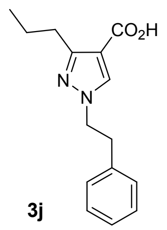

3j

^{1}HNMR (0-10 ppm)
$CDCl_3$, 400 MHz

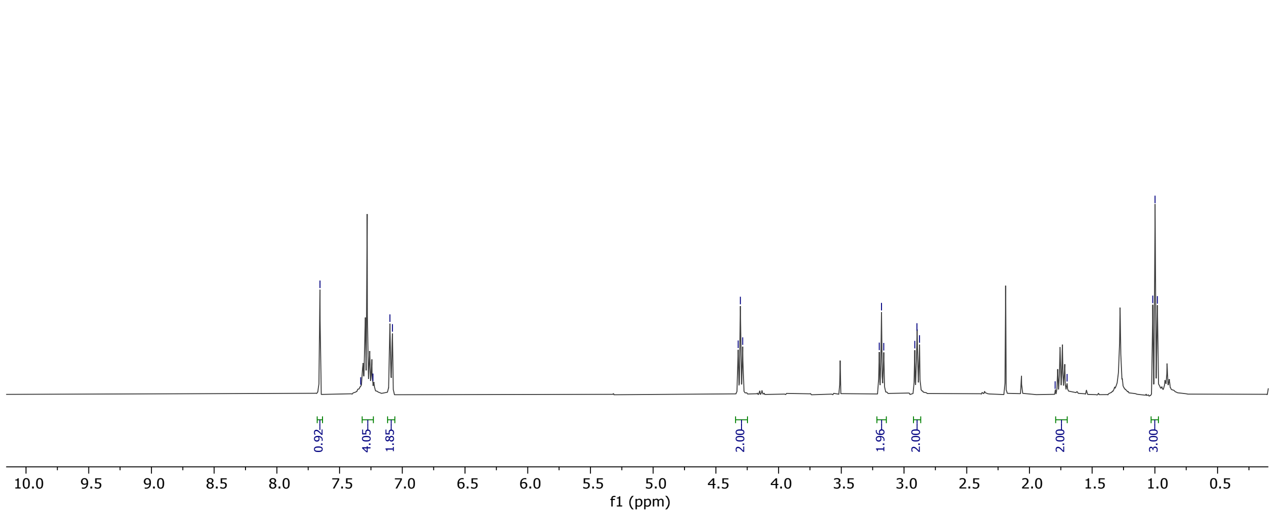

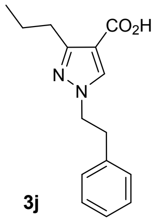

3j

^{13}CNMR-APT (0-200 ppm)
$CDCl_3$, 101 MHz

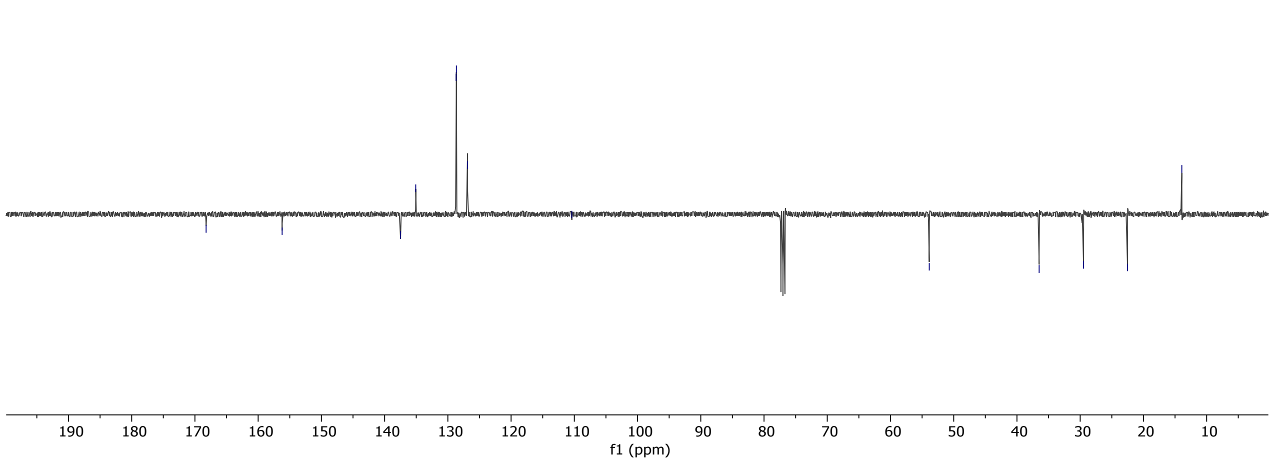

1-Butyl-3-methyl-1*H*-pyrazole-4-carboxylic acid (3k)

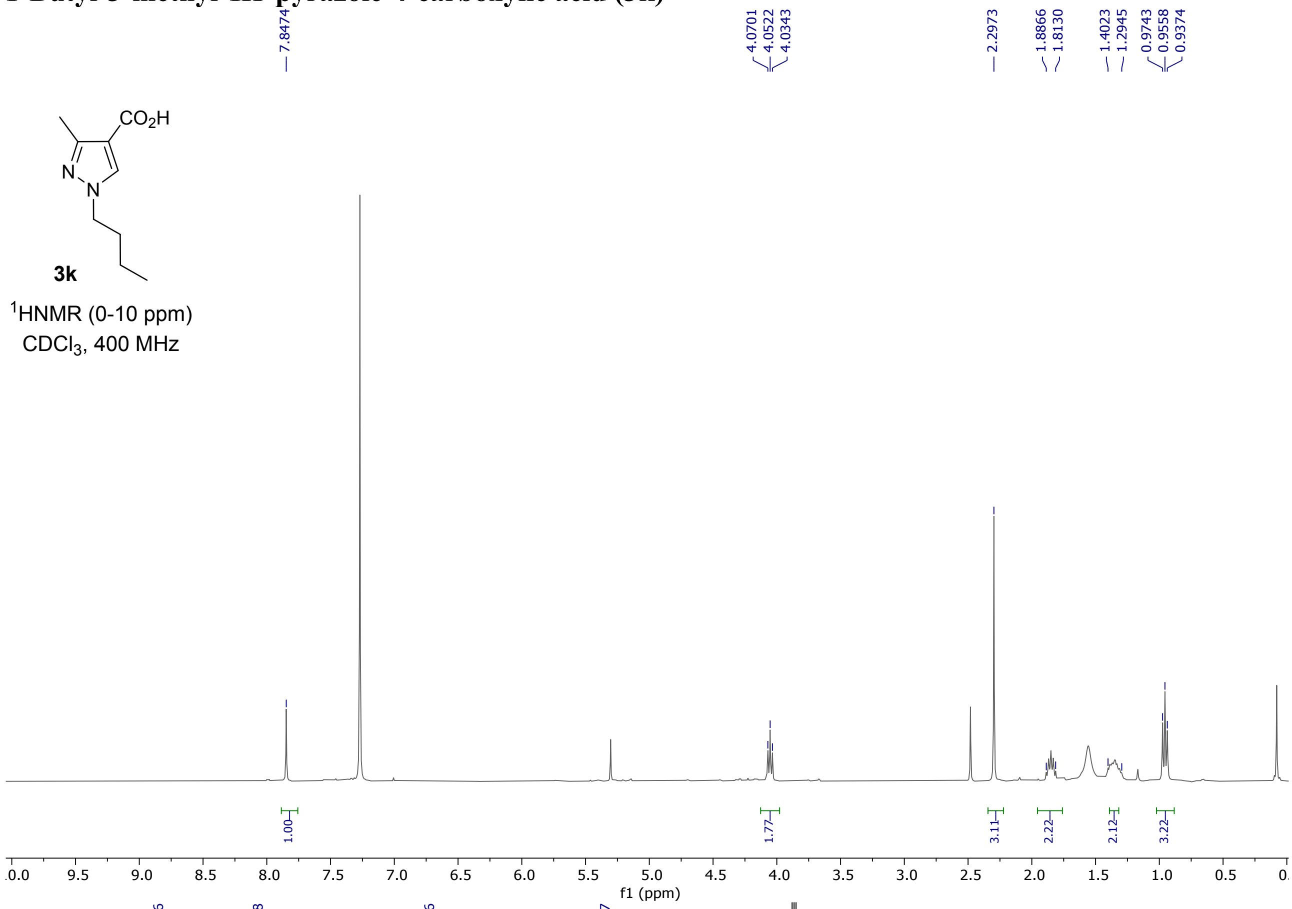

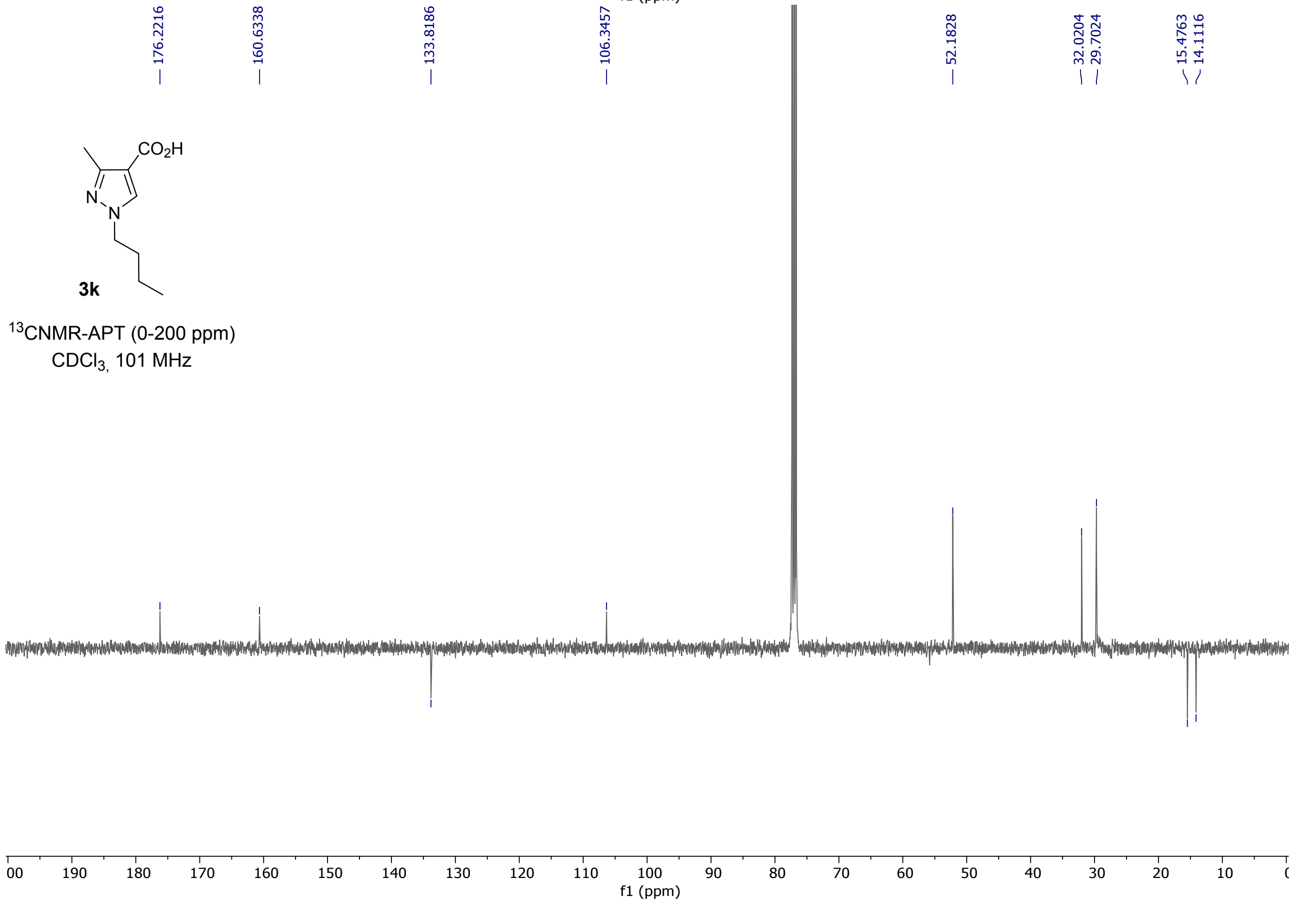

1,5-Diphenyl-3-propyl-1*H*-pyrazole-4-carboxylic acid (3l)

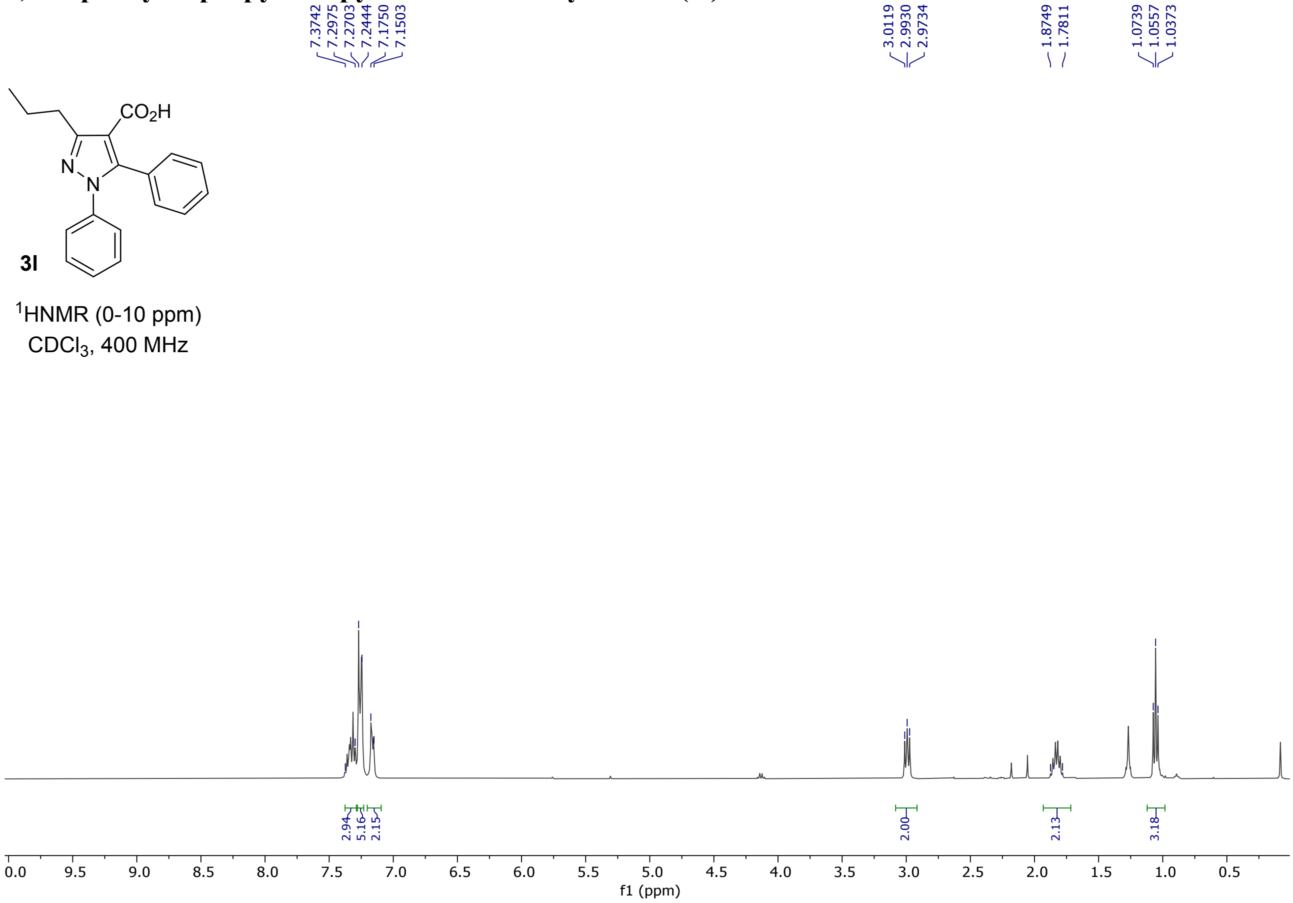

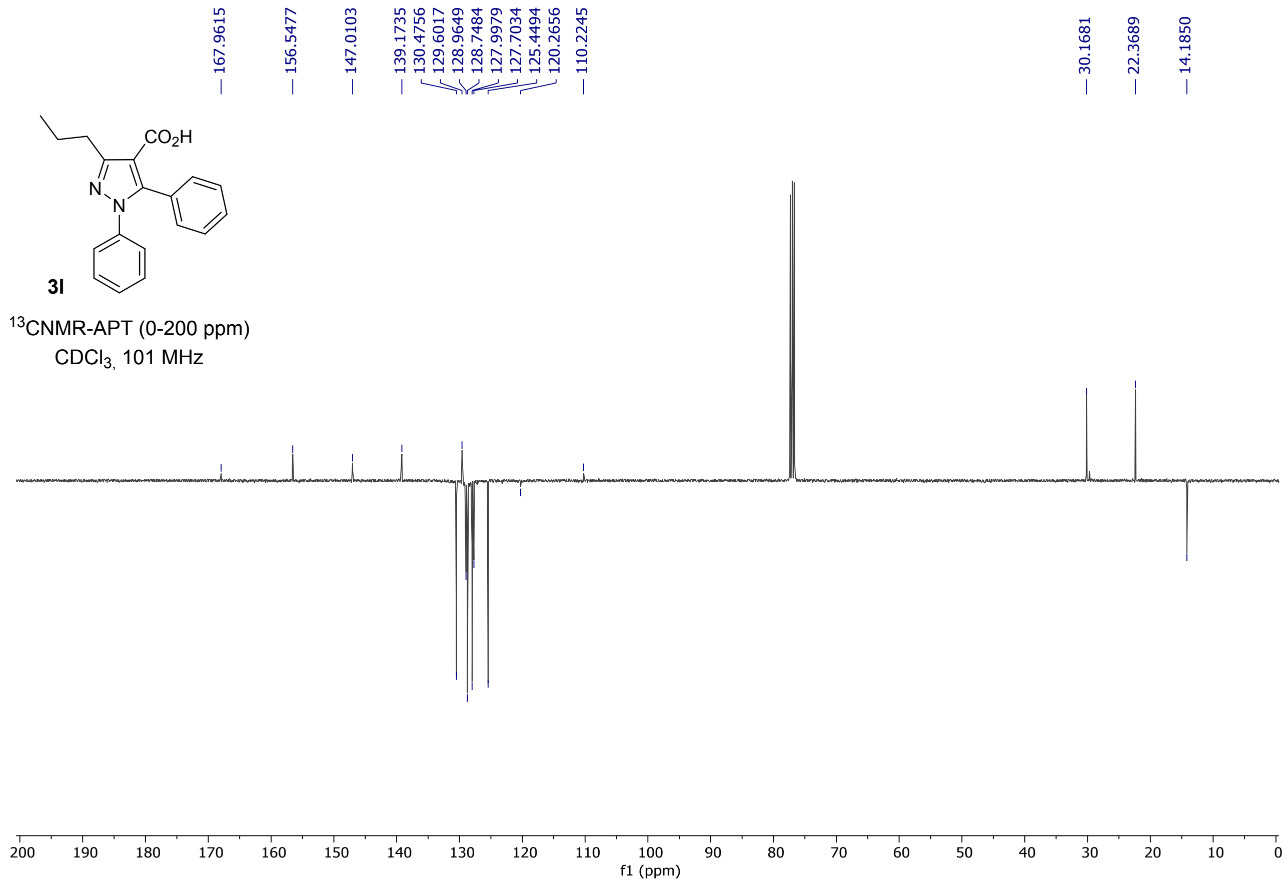

1-Benzyl-5-methyl-3-propyl-1*H*-pyrazole-4-carboxylic acid (3m)

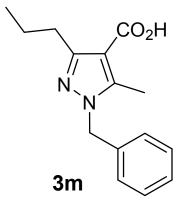

3m

^{1}HNMR (0-10 ppm)
$CDCl_3$, 400 MHz

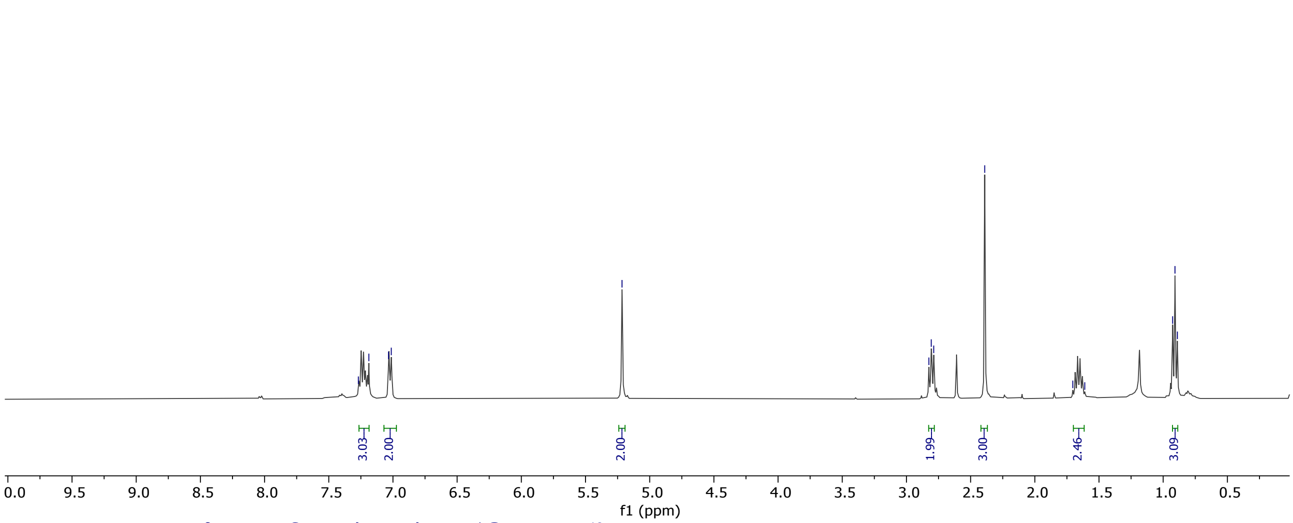

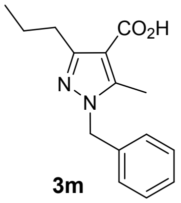

3m

^{13}CNMR-APT (0-200 ppm)
$CDCl_3$, 101 MHz

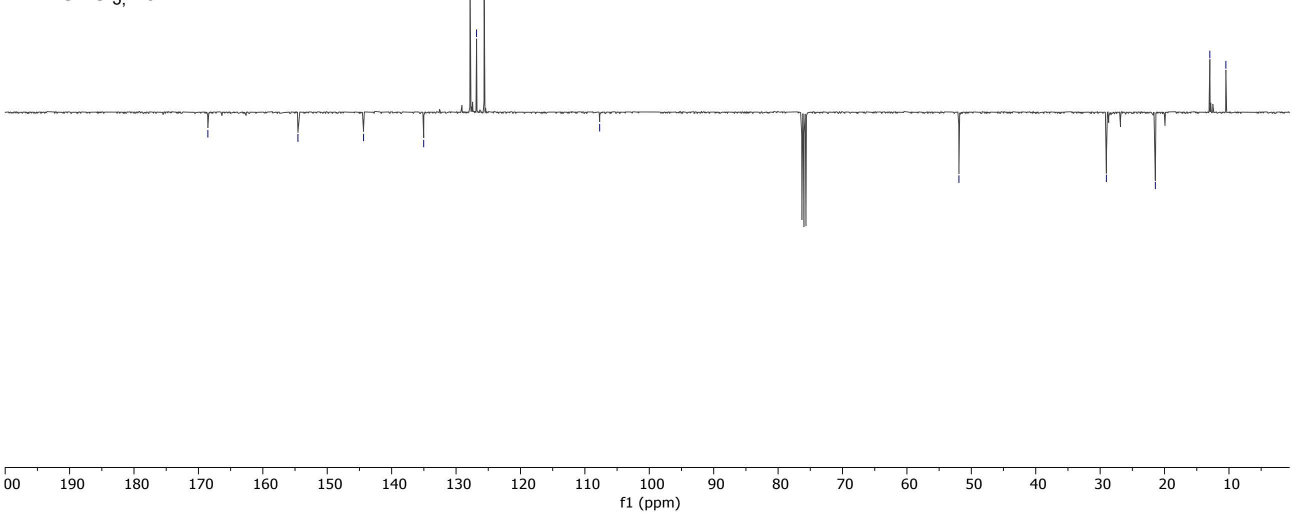

1-Benzyl-5-ethyl-3-methyl-1*H*-pyrazole-4-carboxylic acid (3n)

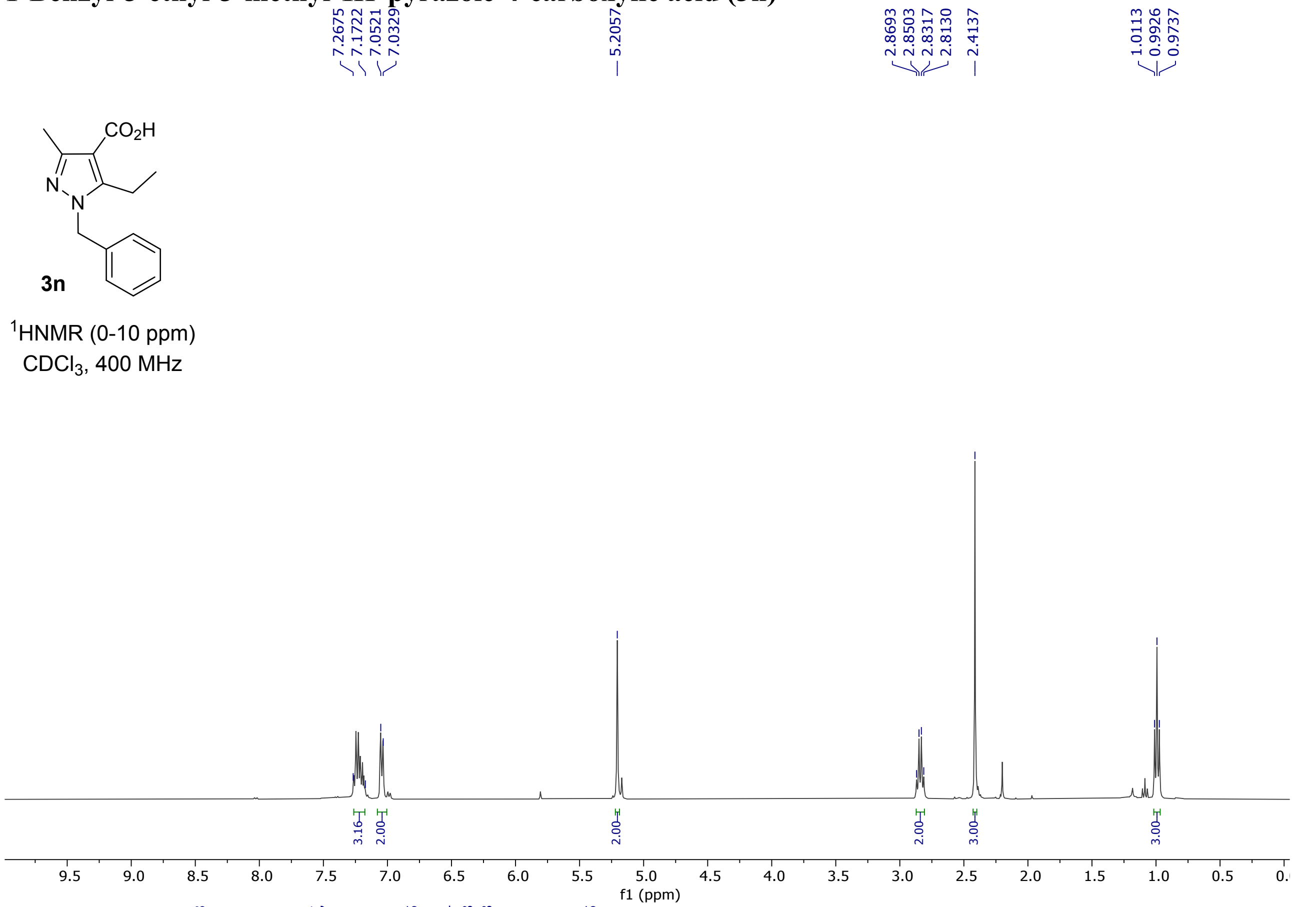

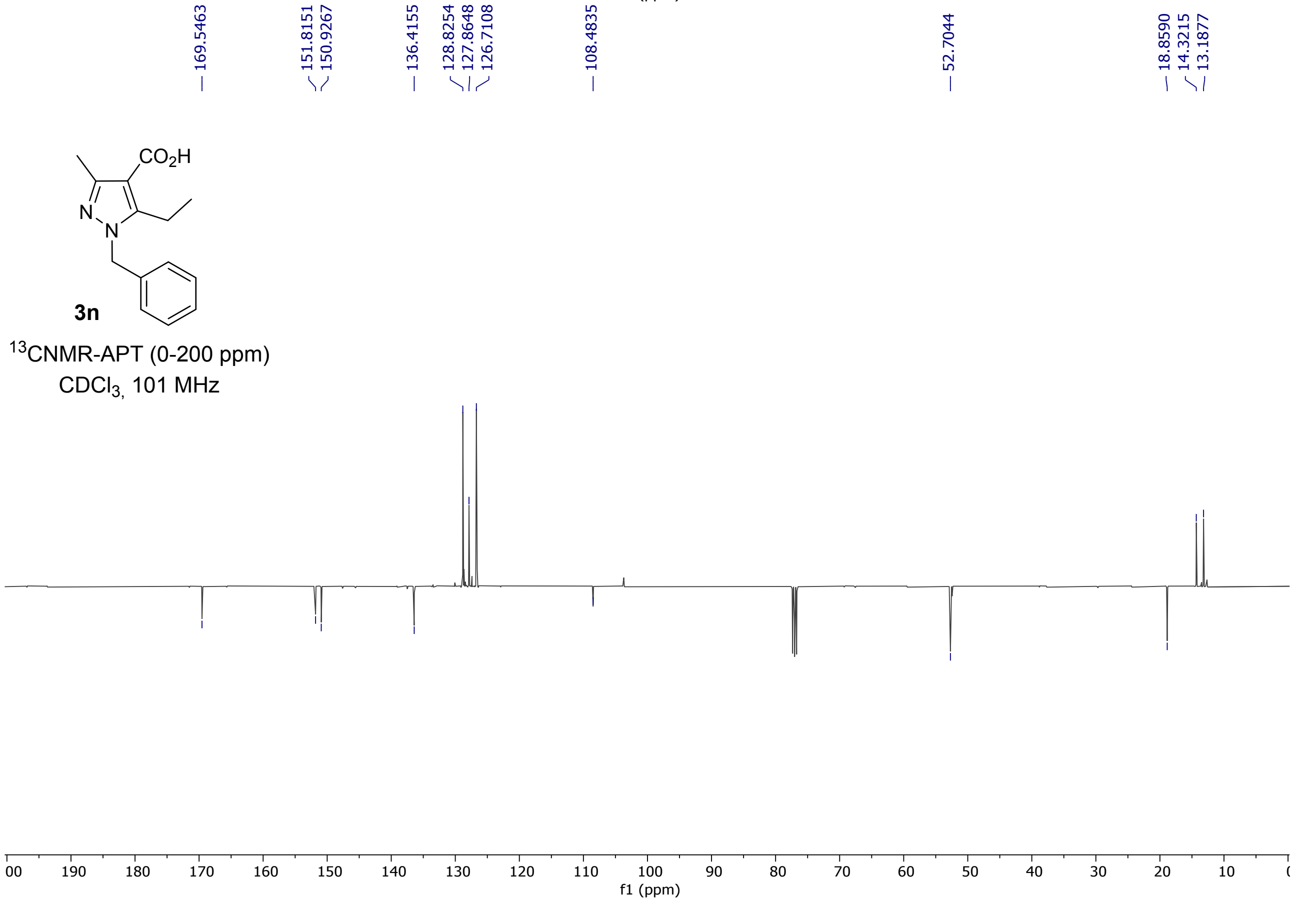

1-Benzyl-5-ethyl-3-propyl-1*H*-pyrazole-4-carboxylic acid (3o)

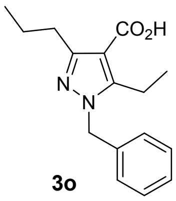

3o

^{1}HNMR (0-10 ppm)
$CDCl_3$, 400 MHz

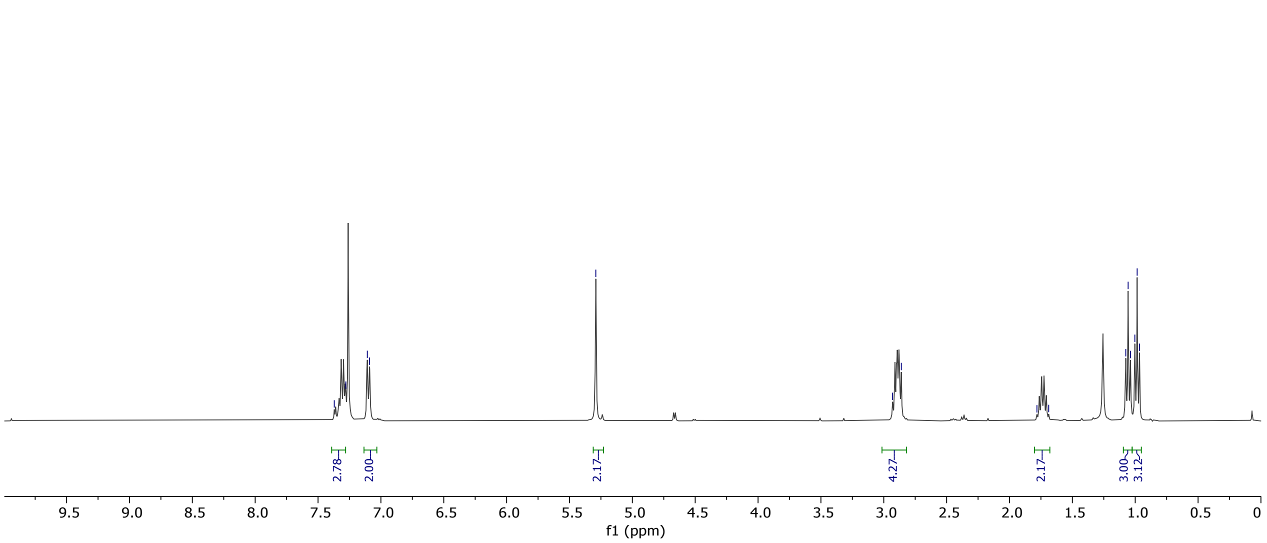

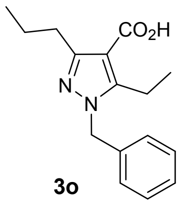

3o

^{13}CNMR-APT (0-200 ppm)
$CDCl_3$, 101 MHz

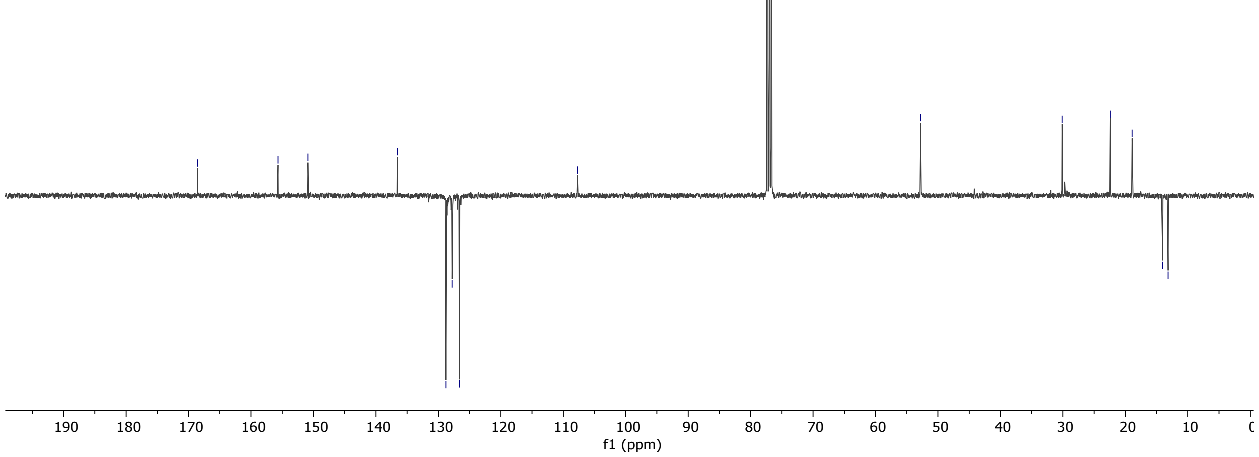

5-Ethyl-3-phenylisoxazole-4-carboxylic acid (6b)

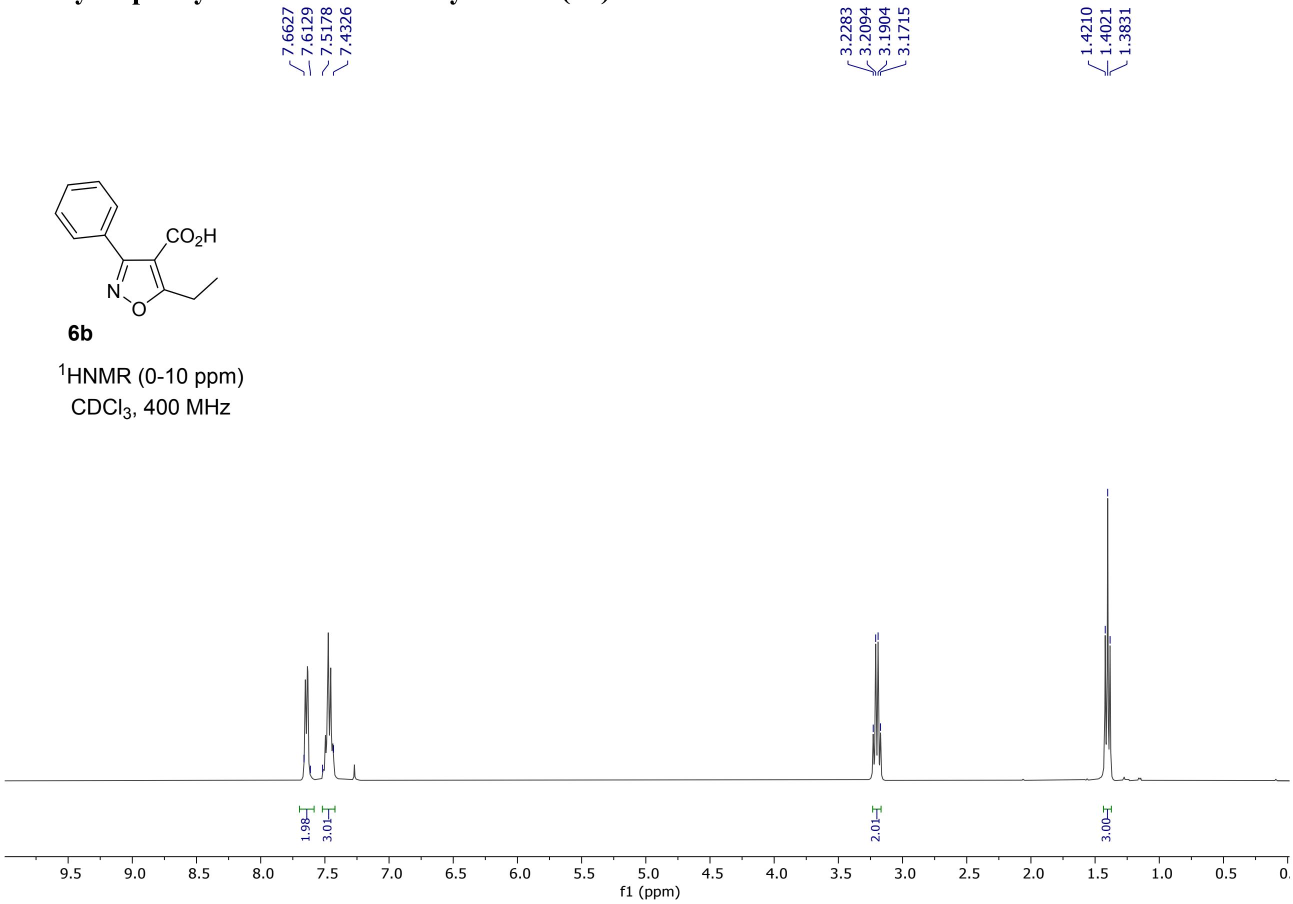

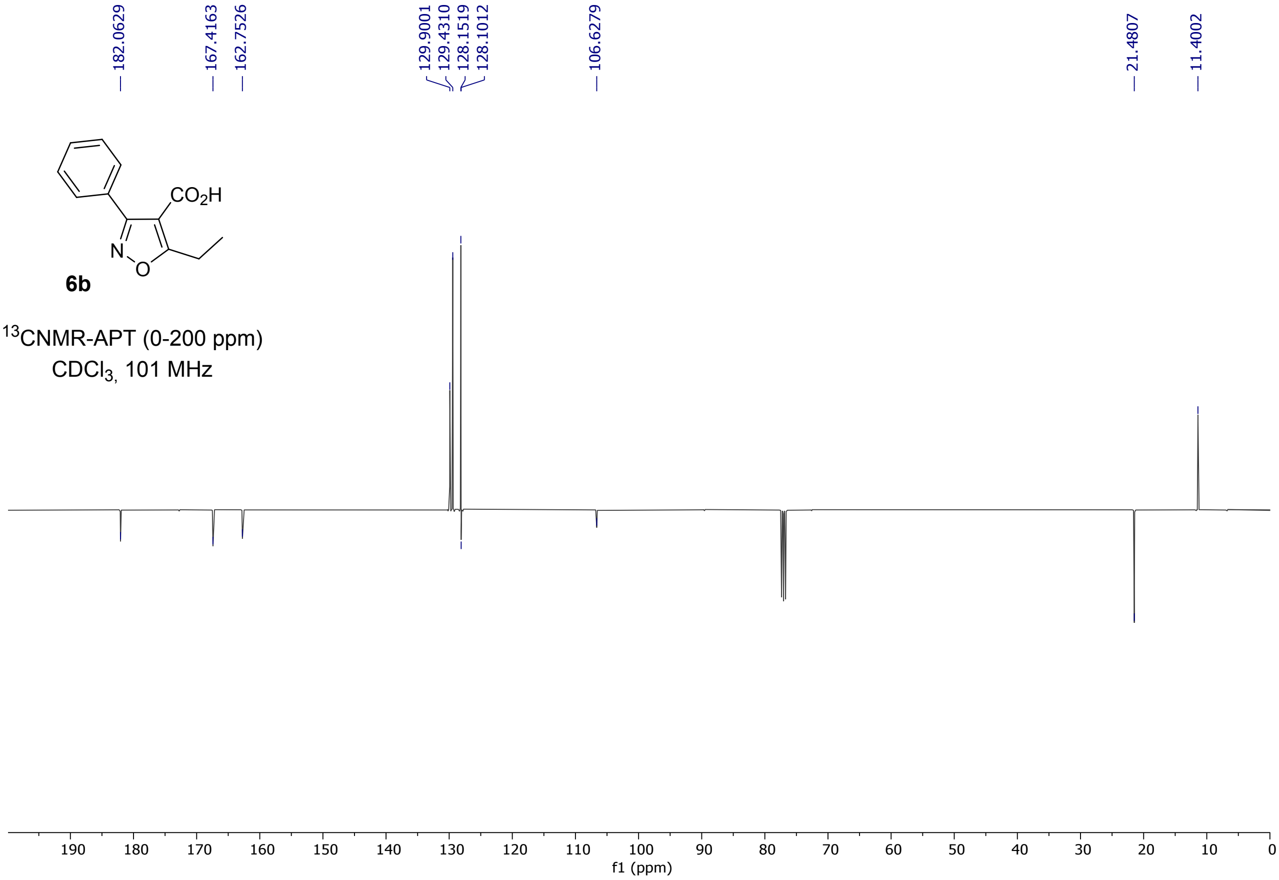

5-Pentyl-3-phenylisoxazole-4-carboxylic acid (6c)

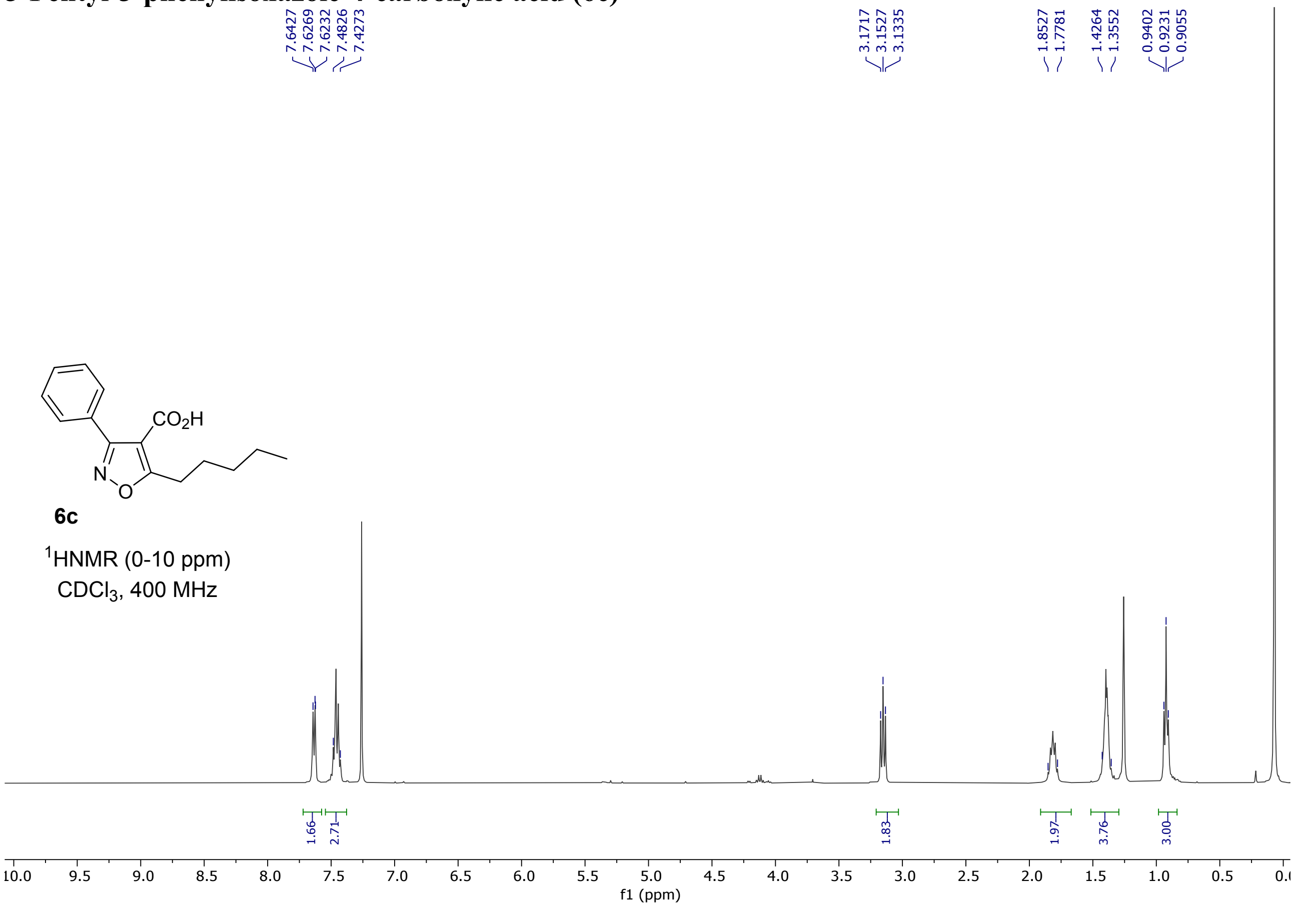

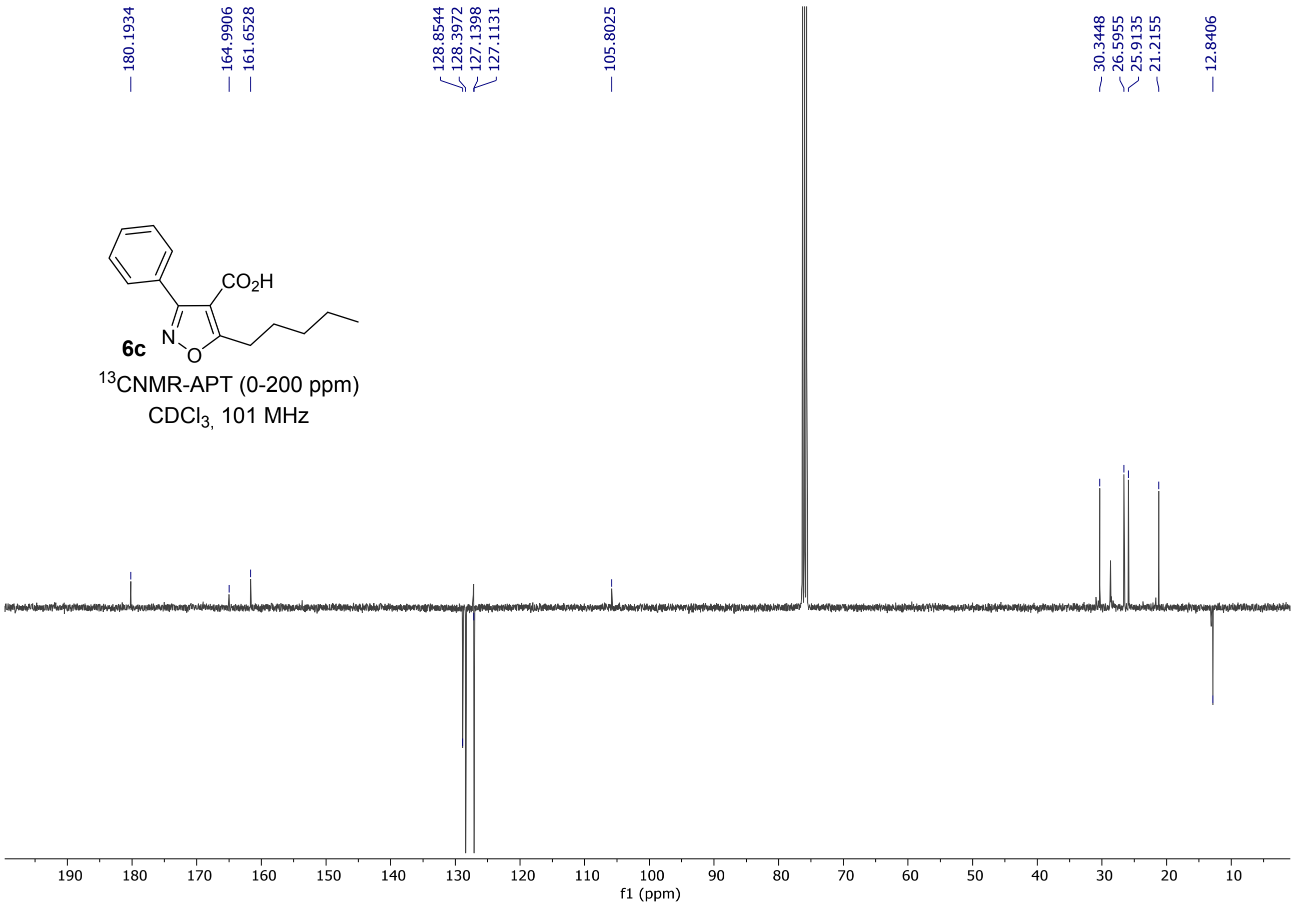

5-Ethyl-3-propylisoxazole-4-carboxylic acid (6e)

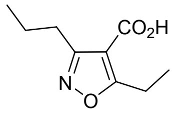

6e

^{1}HNMR (0-10 ppm)
$CDCl_3$, 400 MHz

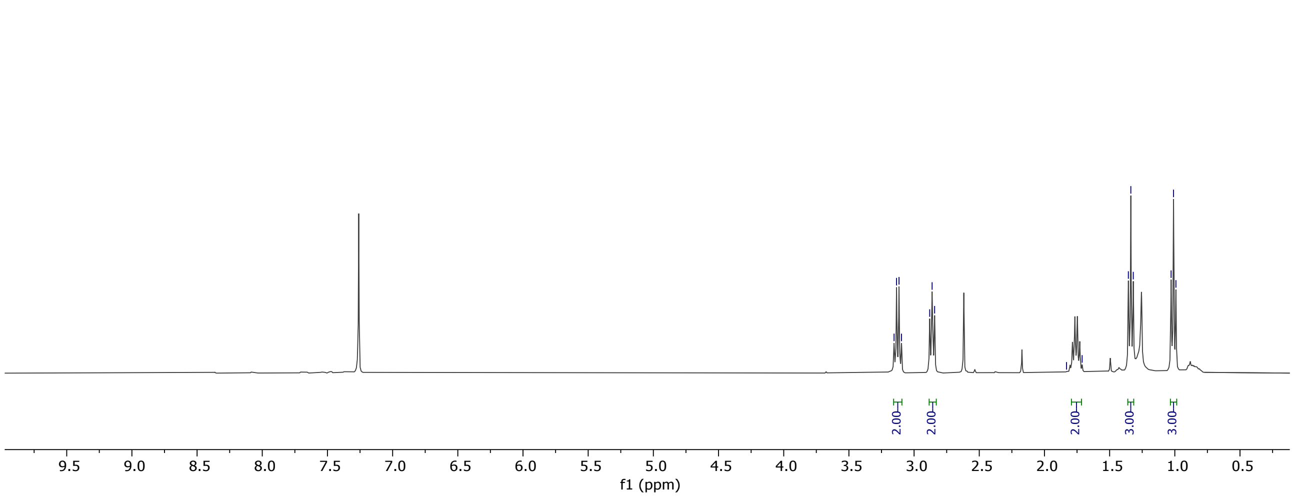

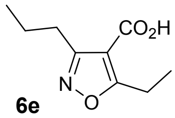

6e

^{13}CNMR-APT (0-200 ppm)
$CDCl_3$, 101 MHz

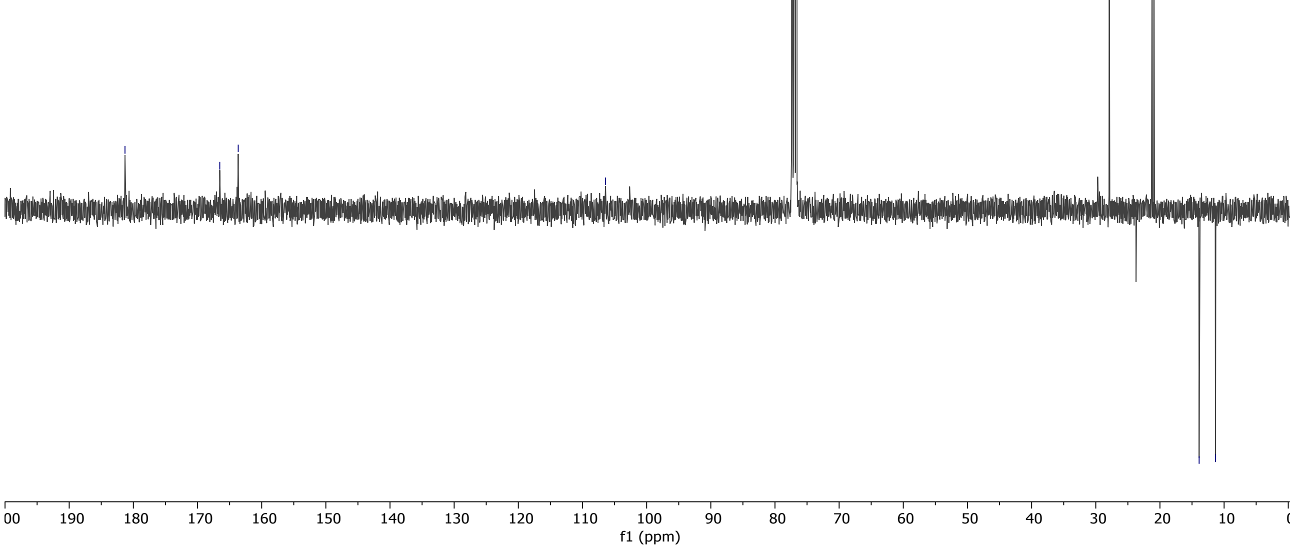

3-Methyl-5-phenylisoxazole-4-carboxylic acid (6f)

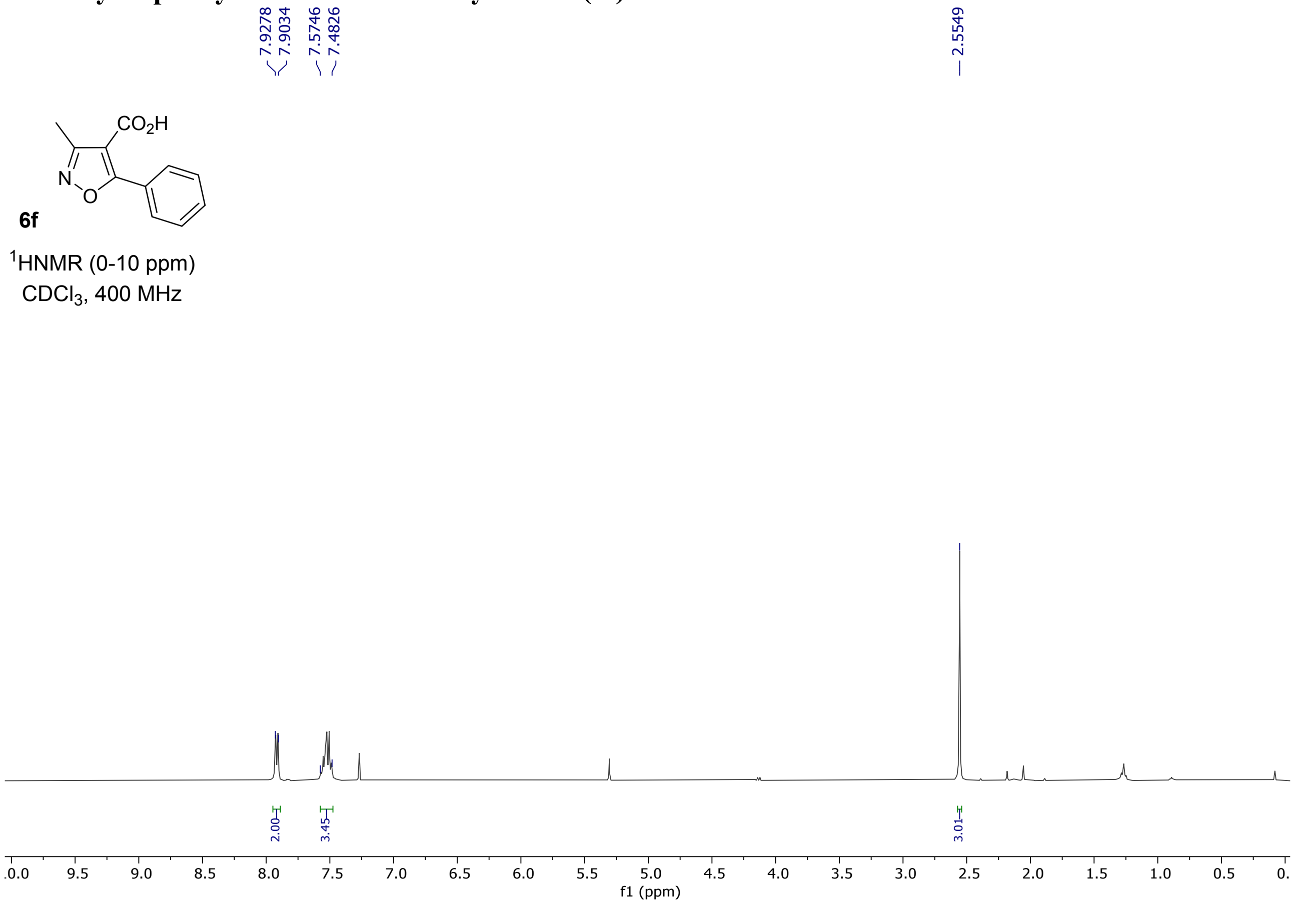

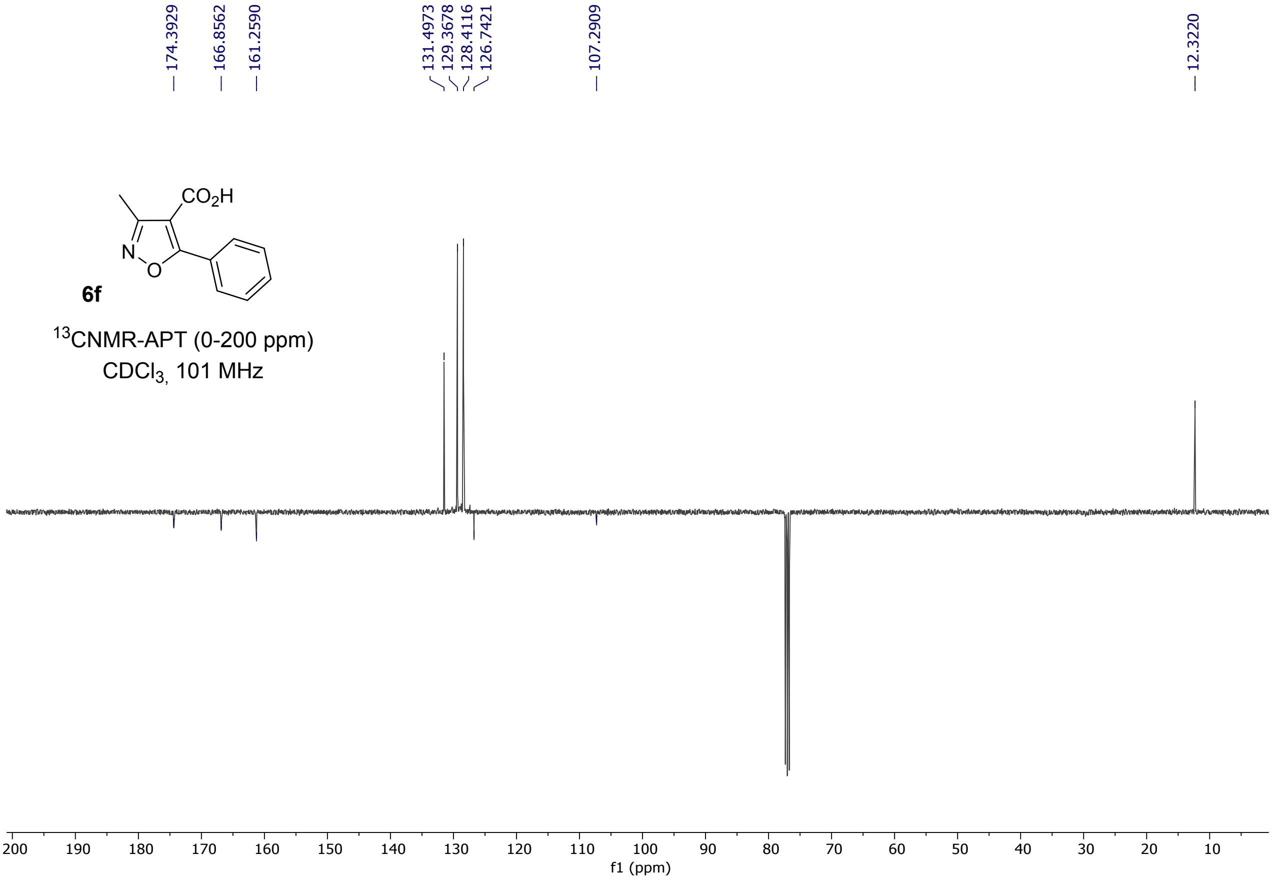

Ethyl (*E*)-3-(4-methyl-5-oxo-3-phenyl-4,5-dihydroisoxazol-4-yl) acrylate (7)

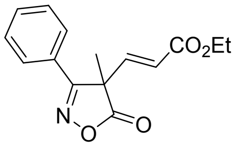

7

^{1}HNMR (0-10 ppm)
$CDCl_3$, 300 MHz

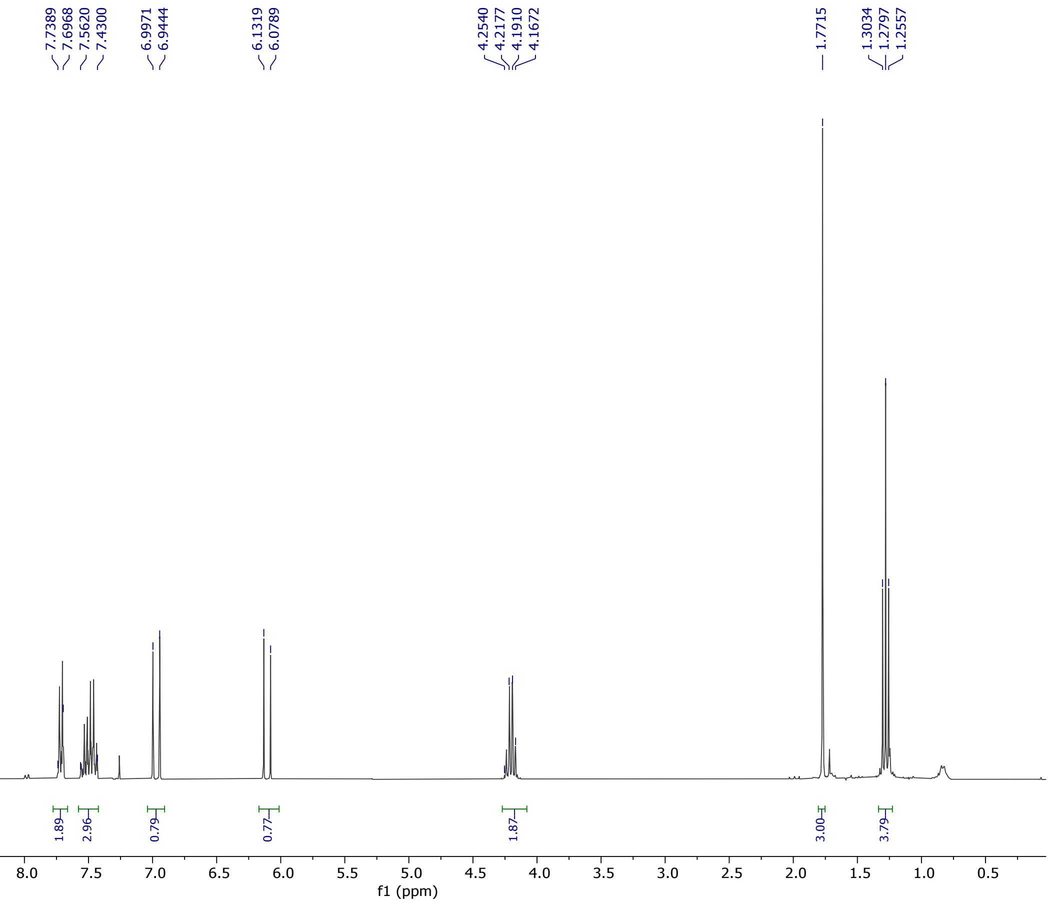

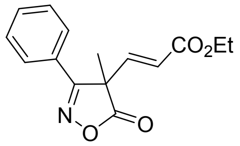

7

^{13}CNMR-APT (0-210 ppm)
$CDCl_3$, 75 MHz

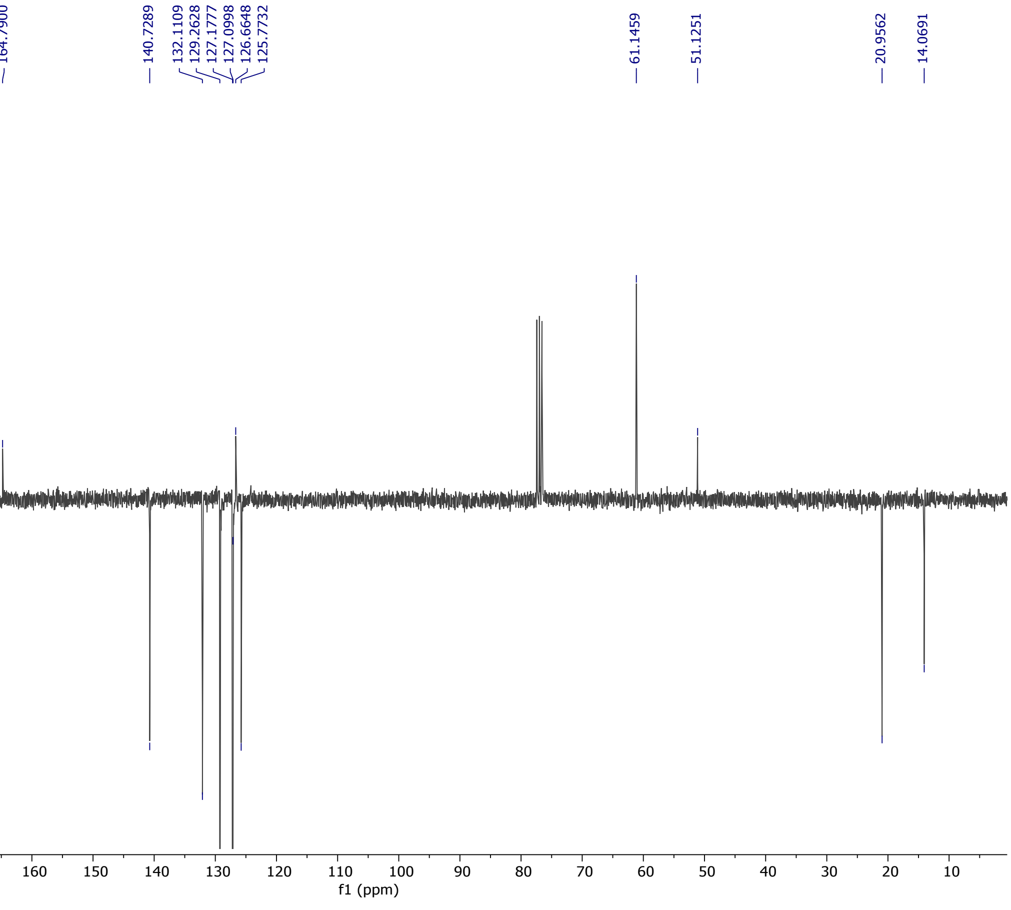

Supplement: Supplementary file 1 — ol2c01135_si_001.pdf [file ol2c01135_si_001.pdf]
